# Supplementary material for: Genome-wide association meta-analysis of human olfactory identification discovers sex-specific and sex-differential genetic variants
Source: Nat Commun. 2025 Jul 1;16:5434. doi: 10.1038/s41467-025-61330-y (PMC12219263; doi:10.1038/s41467-025-61330-y)
Supplement: Supplementary file 1 — Supplementary Information [file 41467_2025_61330_MOESM1_ESM.pdf]

Supplementary Figures

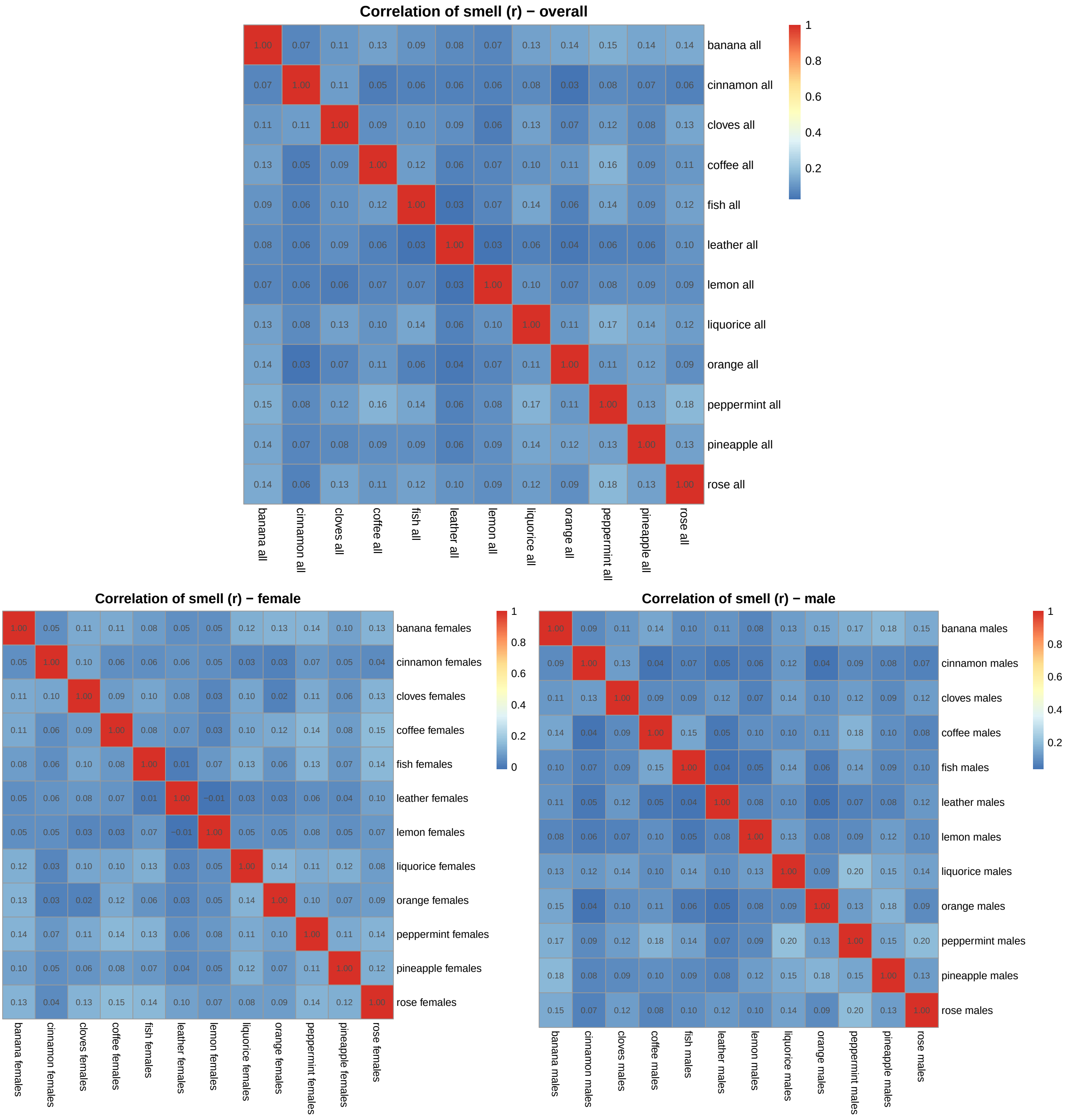

Supplementary Figure 1: Pairwise correlation of odour identification in LIFE-Adult. Shown is the respective Pearson correlation coefficient (r) between each pair of odours for the overall analysis (top), females (bottom left) and males (bottom right).

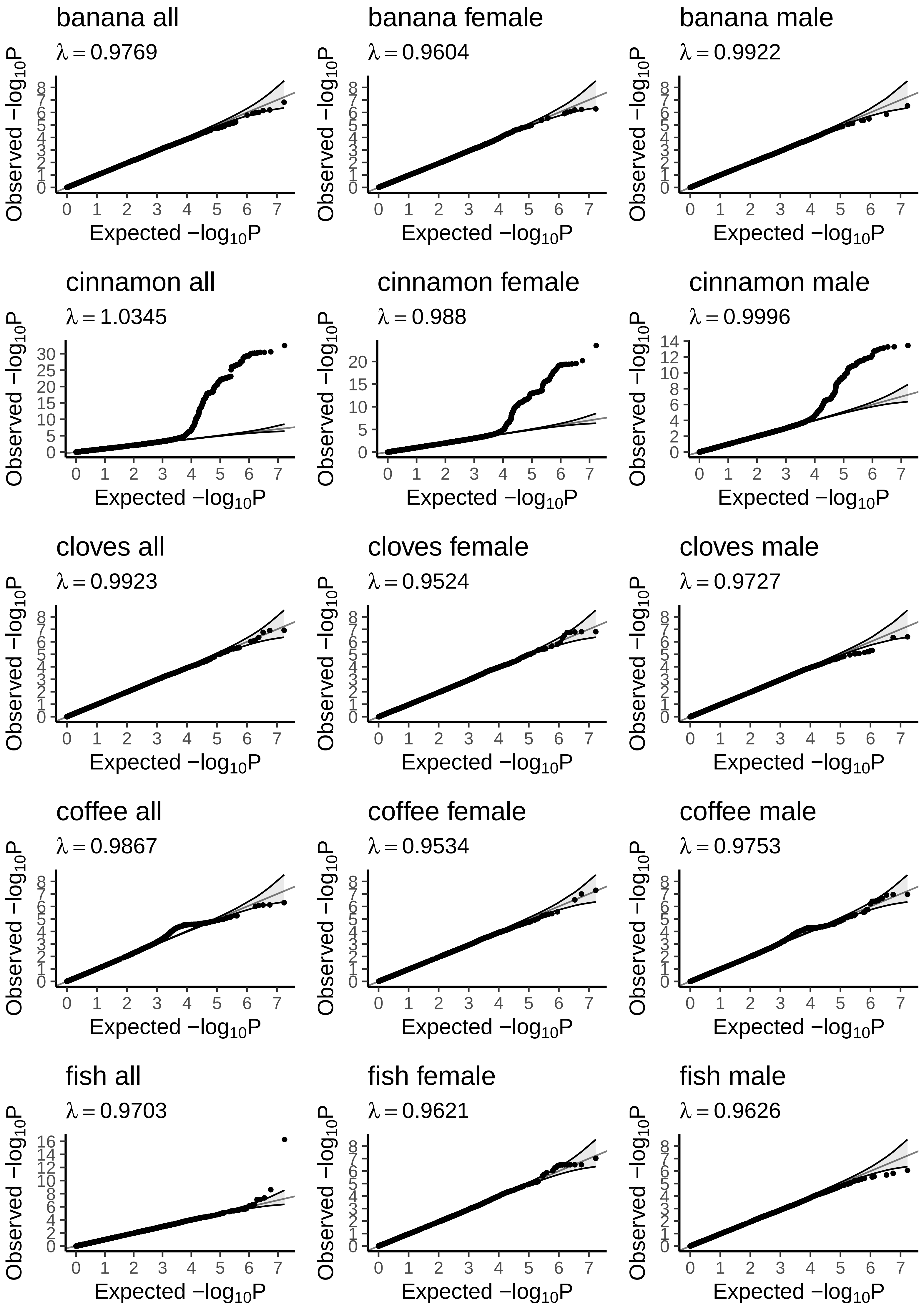
banana all
$\lambda = 0.9769$
banana female
$\lambda = 0.9604$
banana male
$\lambda = 0.9922$
cinnamon all
$\lambda = 1.0345$
cinnamon female
$\lambda = 0.988$
cinnamon male
$\lambda = 0.9996$
cloves all
$\lambda = 0.9923$
cloves female
$\lambda = 0.9524$
cloves male
$\lambda = 0.9727$
coffee all
$\lambda = 0.9867$
coffee female
$\lambda = 0.9534$
coffee male
$\lambda = 0.9753$
fish all
$\lambda = 0.9703$
fish female
$\lambda = 0.9621$
fish male
$\lambda = 0.9626$
Observed $-\log_{10}P$
Expected $-\log_{10}P$

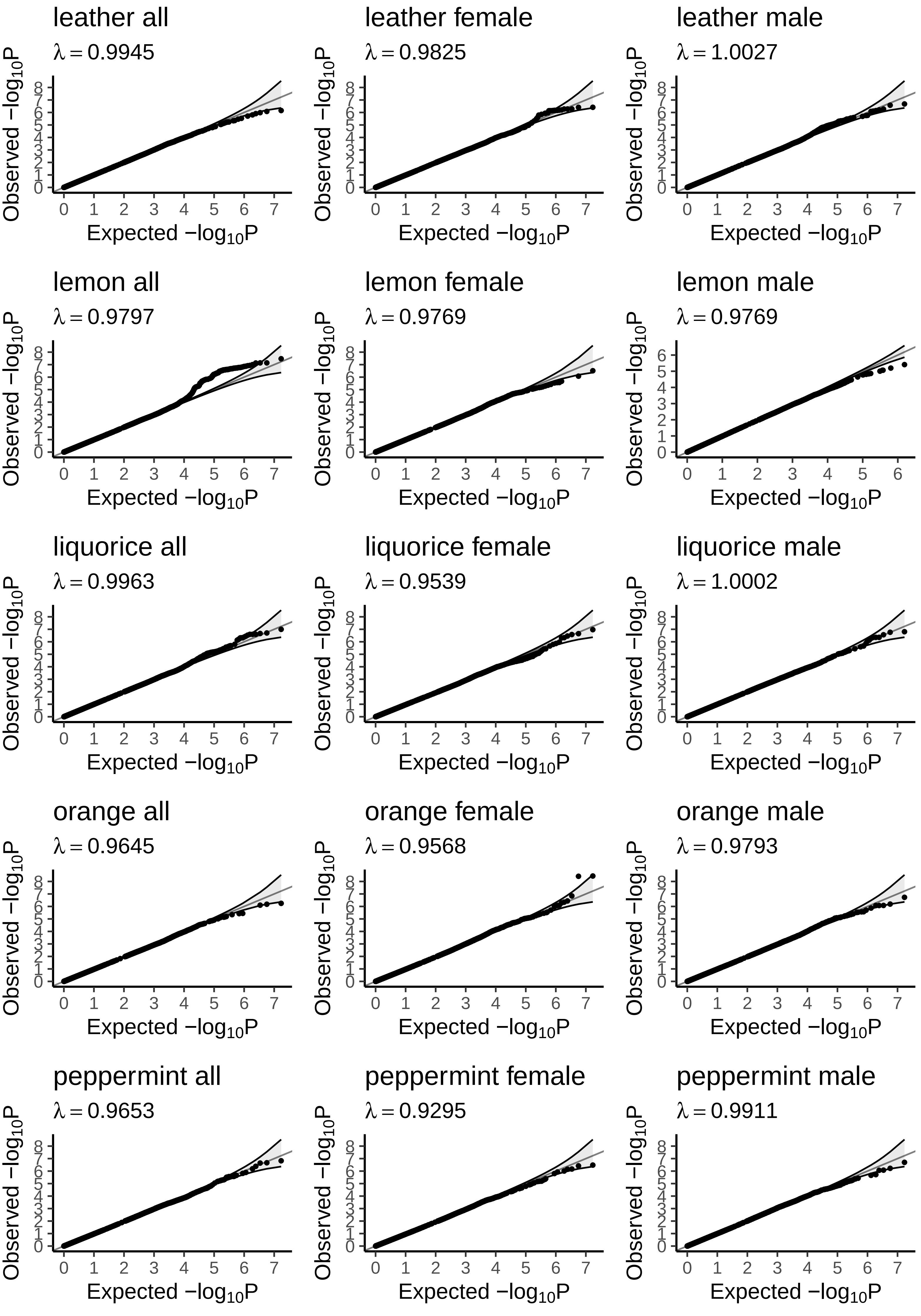
leather all
λ = 0.9945
leather female
λ = 0.9825
leather male
λ = 1.0027
lemon all
λ = 0.9797
lemon female
λ = 0.9769
lemon male
λ = 0.9769
liquorice all
λ = 0.9963
liquorice female
λ = 0.9539
liquorice male
λ = 1.0002
orange all
λ = 0.9645
orange female
λ = 0.9568
orange male
λ = 0.9793
peppermint all
λ = 0.9653
peppermint female
λ = 0.9295
peppermint male
λ = 0.9911
Observed −log10P
Expected −log10P

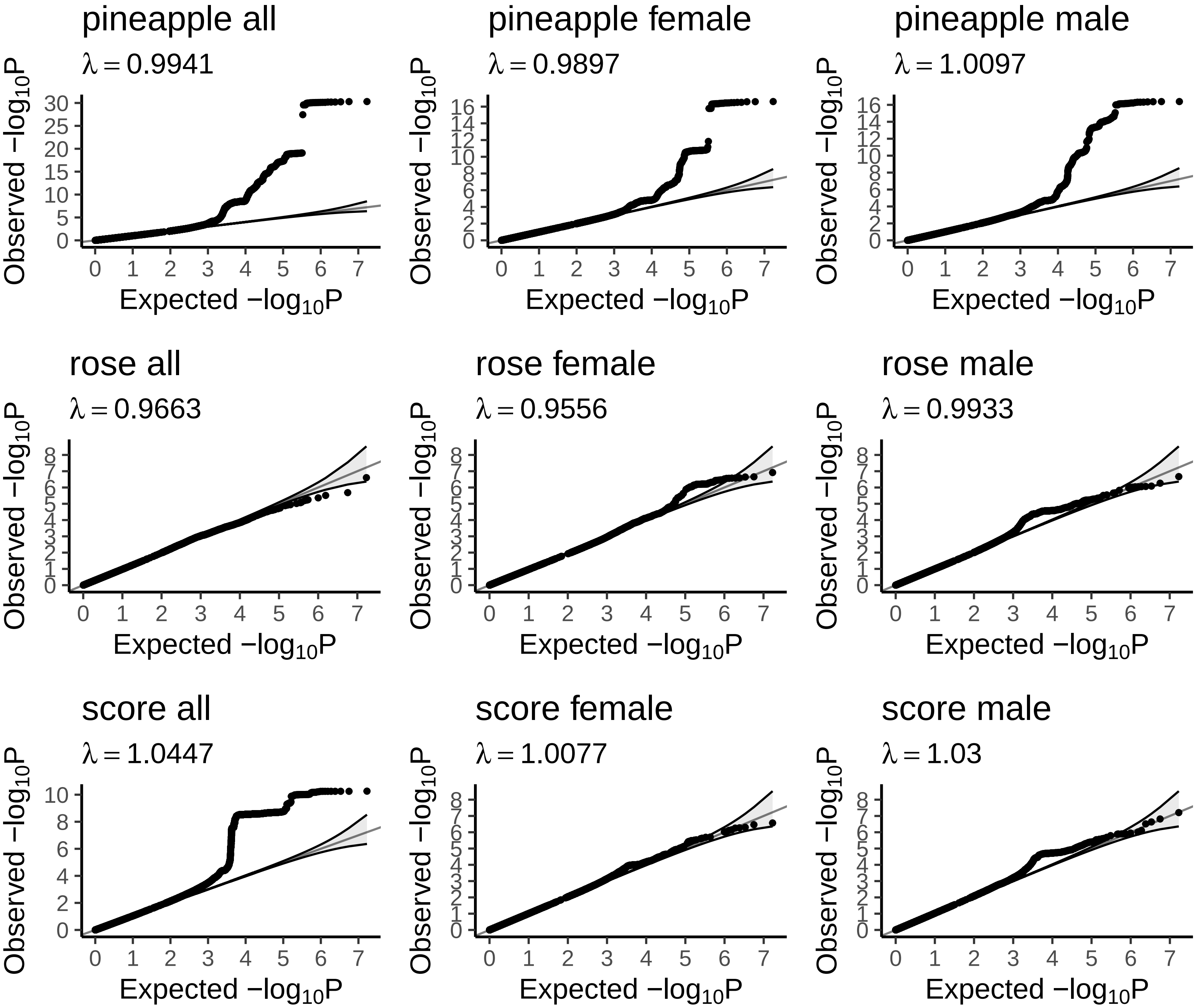

Supplementary Figure 2: Q-Q-Plots and λ-values for all combinations of phenotypes and subgroups. Plots and values correspond to data after QC-processing. 95% confidence interval is shown.

Region 1: rs73252922

trait: fish_all; rsq: 0.95; MAF: 0.03; gw. significance: trait−wise

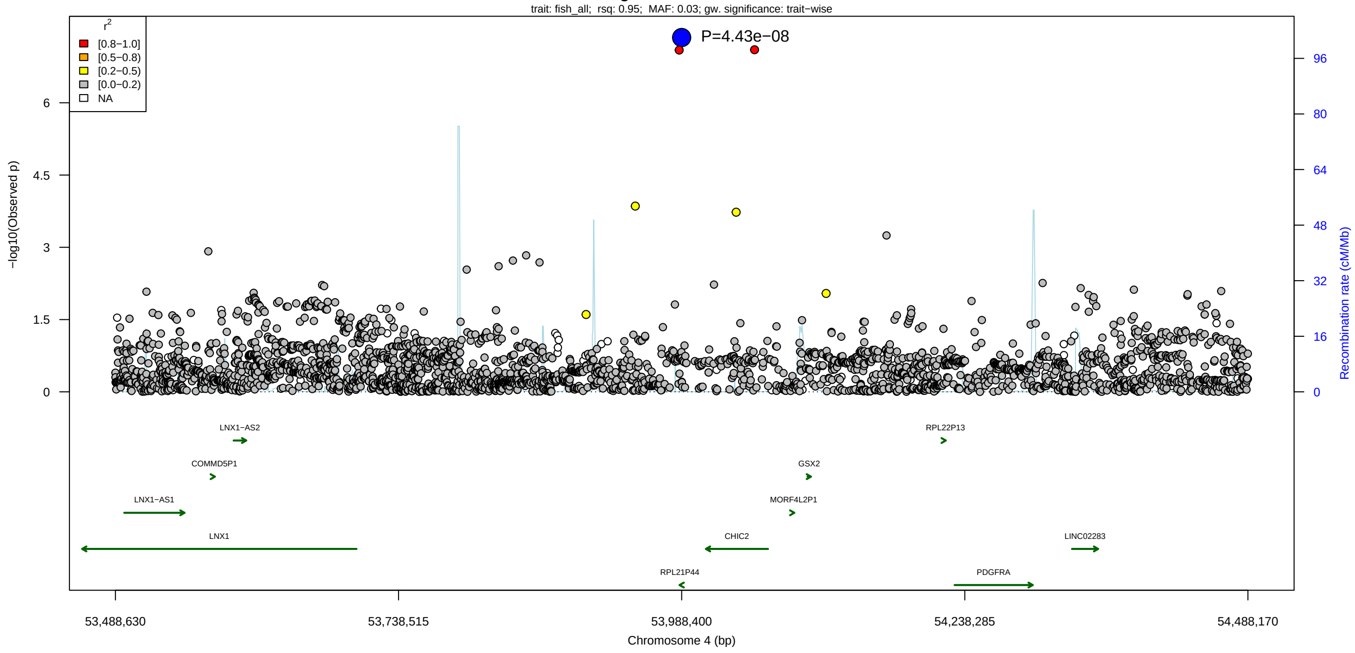

Region 2: rs116058752

trait: orange_female; rsq: 0.89; MAF: 0.03; gw. significance: study−wide

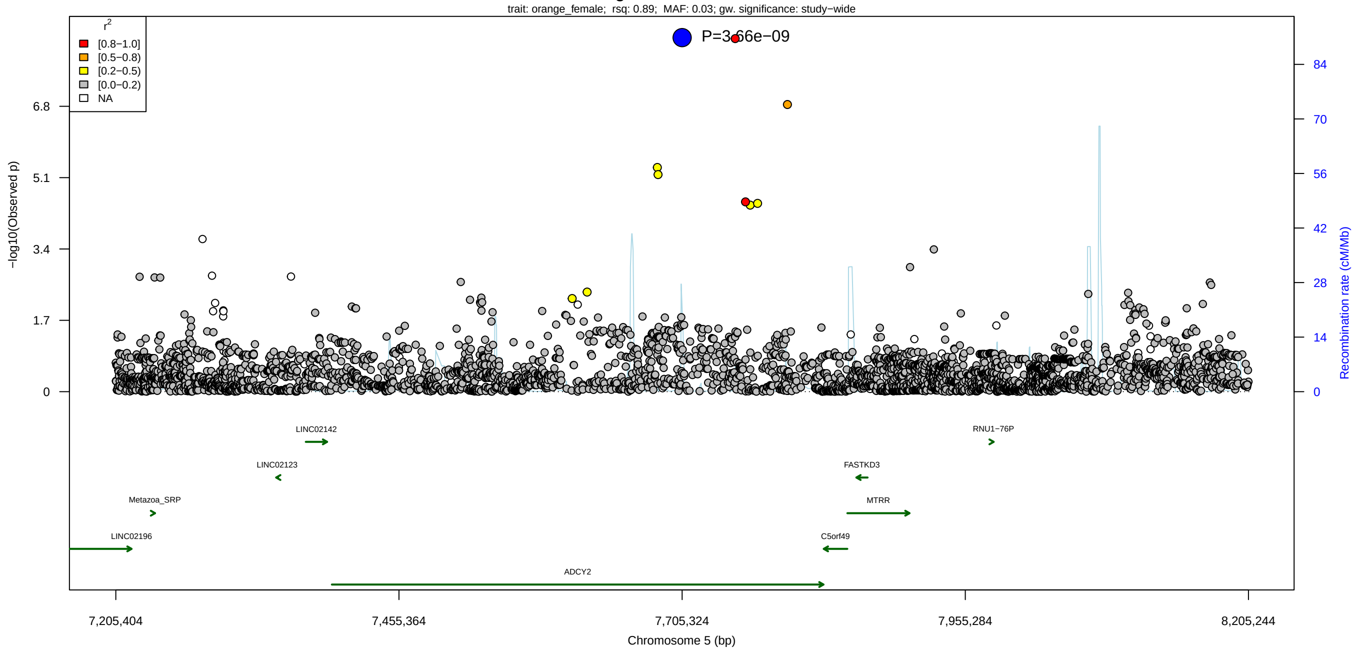

Region 3: rs17161232

trait: lemon_all; rsq: 1; MAF: 0.18; gw. significance: trait−wise

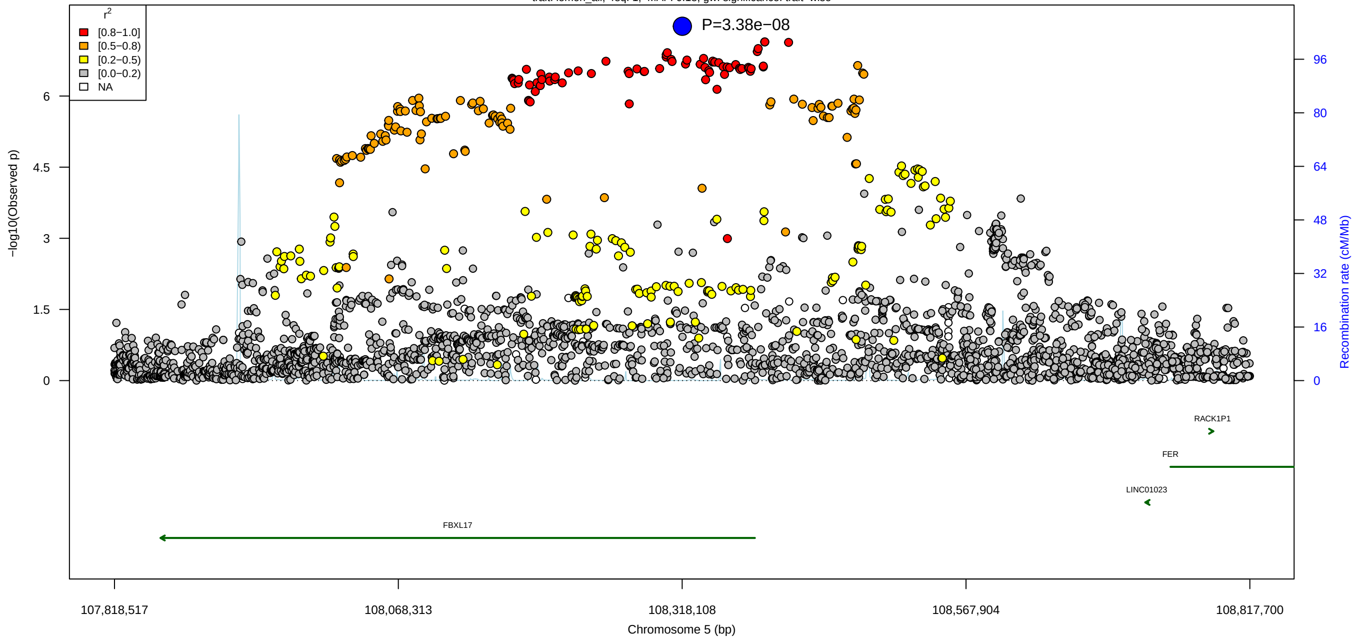

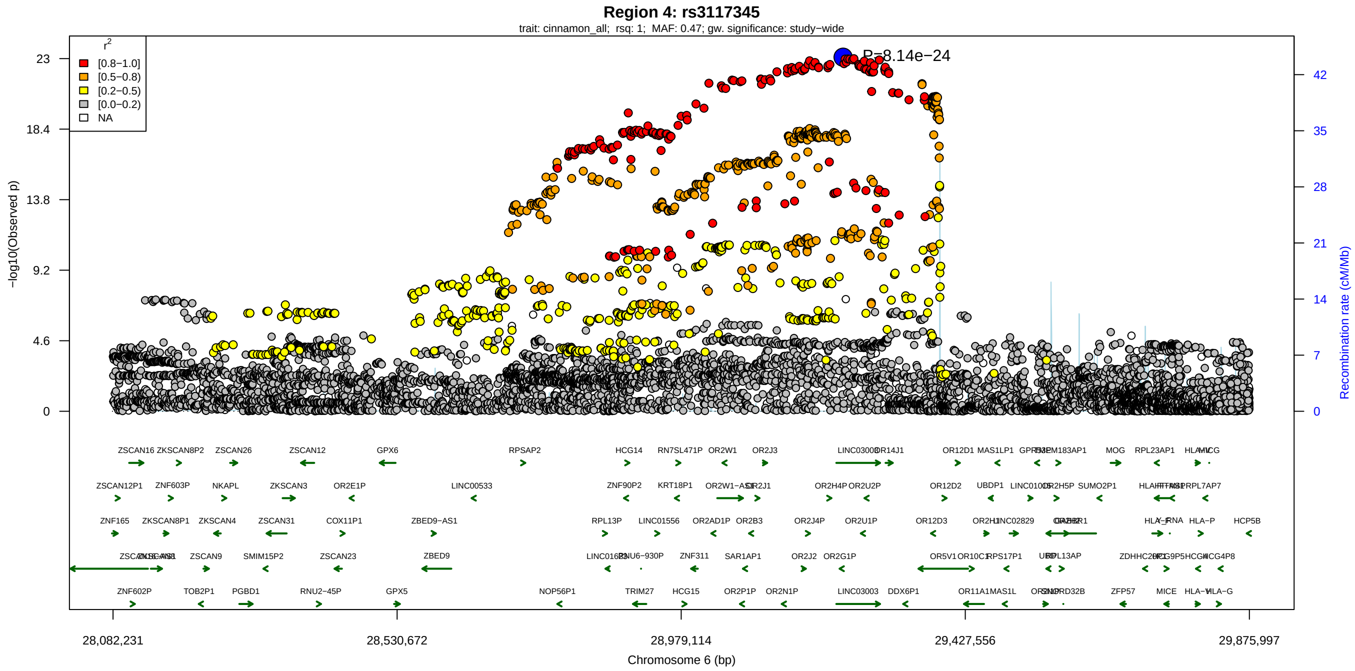
Region 4: rs3117345
trait: cinnamon_all; rsq: 1; MAF: 0.47; gw. significance: study−wide
r2
[0.8−1.0]
[0.5−0.8)
[0.2−0.5)
[0.0−0.2)
NA
P=8.14e−24
−log10(Observed p)
23
18.4
13.8
9.2
4.6
0
Recombination rate (cM/Mb)
42
35
28
21
14
7
0
ZSCAN16 ZKSCAN8P2 ZSCAN26 ZSCAN12 GPX6 RPSAP2 HCG14 RN7SL471P OR2W1 OR2J3 OR14J1 OR12D1 MAS1LP1 TMEM183AP1 MOG RPL23AP1 HLA-G
ZSCAN12P1 ZNF603P NKAPL ZKSCAN3 OR2E1P LINC00533 ZNF90P2 KRT18P1 OR2J1 OR2H4P OR2U2P OR12D2 UBDP1 OR2H5P SUMO2P1 RPL7AP7
ZNF165 ZKSCAN8P1 ZKSCAN4 ZSCAN31 COX11P1 ZBED9−AS1 RPL13P LINC01556 OR2AD1P OR2B3 OR2J4P OR2U1P OR12D3 LINC02829 HLA-P HCP5B
ZSCAN9 SMIM15P2 ZSCAN23 ZBED9 ZNF311 SAR1AP1 OR2J2 OR2G1P RPS17P1 RPL13AP ZDHHC20P1 HCG4P8
ZNF602P TOB2P1 PGBD1 RNU2−45P GPX5 NOP56P1 TRIM27 HCG15 OR2P1P OR2N1P LINC03003 DDX6P1 OR11A1 MAS1L SNORD32B ZFP57 MICE HLA-V HLA-G
28,082,231
28,530,672
28,979,114
29,427,556
29,875,997
Chromosome 6 (bp)

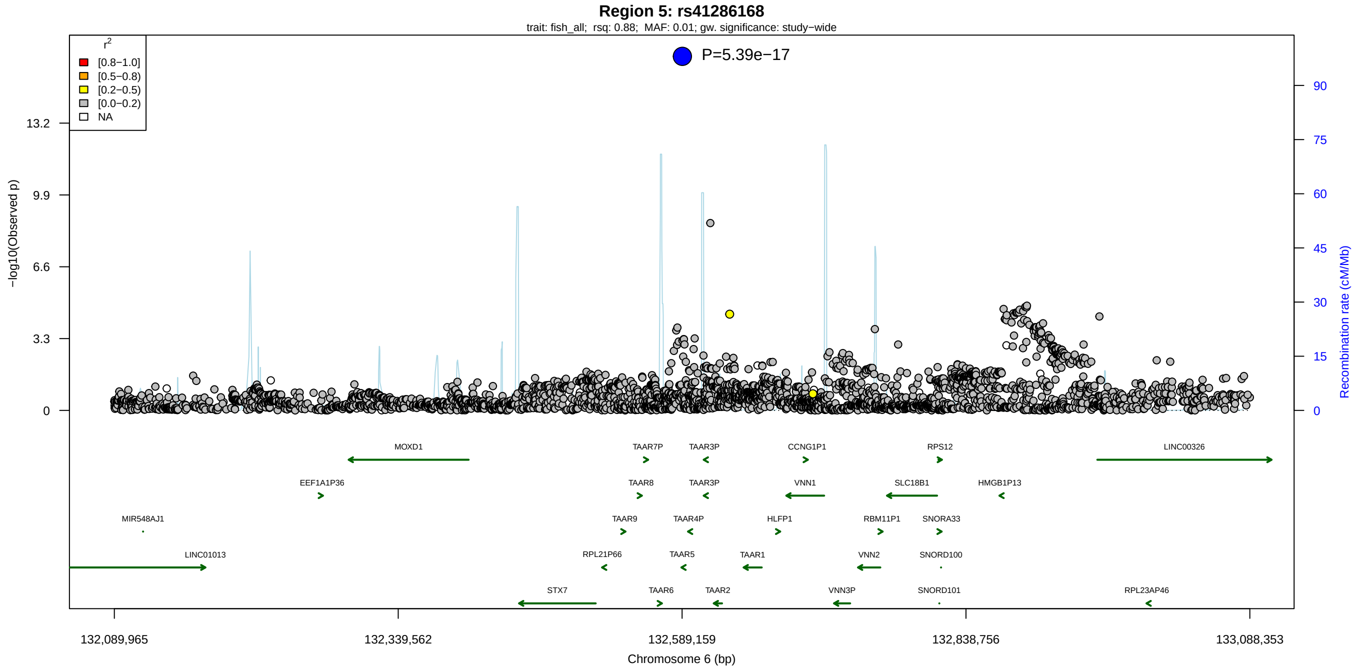
Region 5: rs41286168
trait: fish_all; rsq: 0.88; MAF: 0.01; gw. significance: study−wide
r2
[0.8−1.0]
[0.5−0.8)
[0.2−0.5)
[0.0−0.2)
NA
P=5.39e−17
−log10(Observed p)
13.2
9.9
6.6
3.3
0
Recombination rate (cM/Mb)
90
75
60
45
30
15
0
MOXD1 TAAR7P TAAR3P CCNG1P1 RPS12 LINC00326
EEF1A1P36 TAAR8 TAAR3P VNN1 SLC18B1 HMGB1P13
MIR548AJ1 TAAR9 TAAR4P HLFP1 RBM11P1 SNORA33
LINC01013 RPL21P66 TAAR5 TAAR1 VNN2 SNORD100
STX7 TAAR6 TAAR2 VNN3P SNORD101 RPL23AP46
132,089,965
132,339,562
132,589,159
132,838,756
133,088,353
Chromosome 6 (bp)

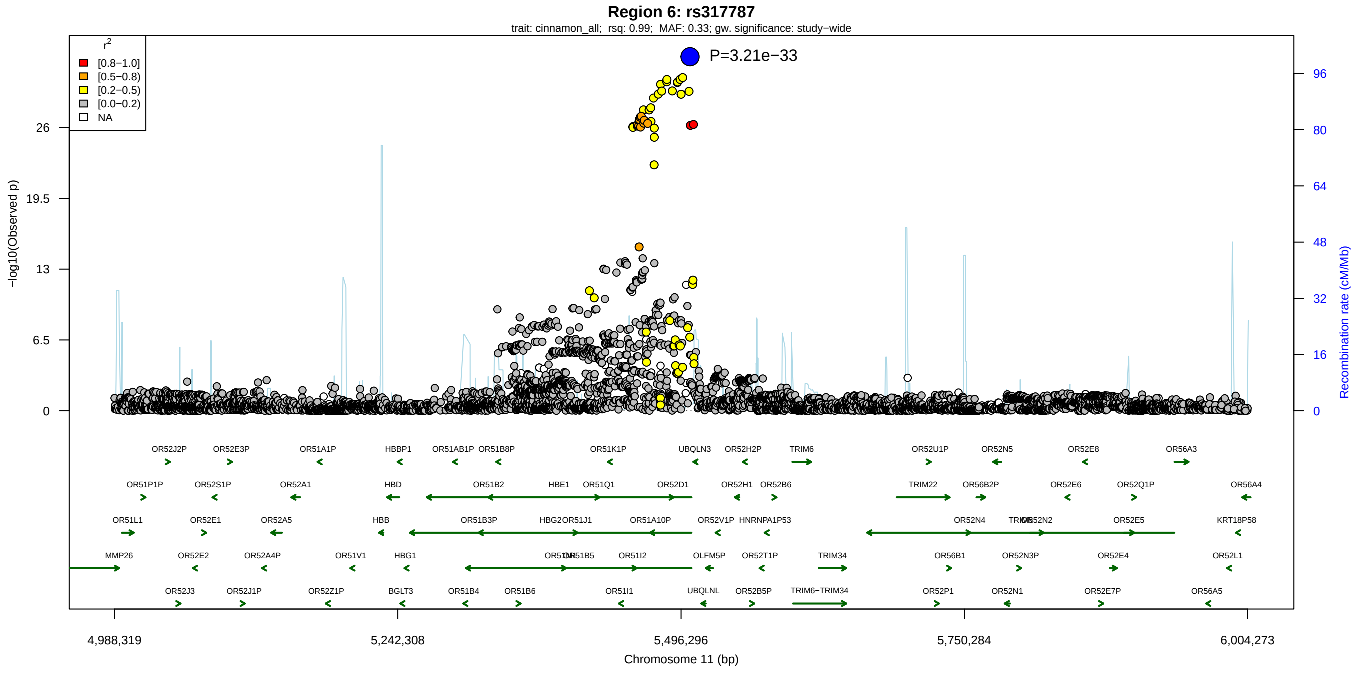
Region 6: rs317787
trait: cinnamon_all; rsq: 0.99; MAF: 0.33; gw. significance: study−wide
r2
[0.8−1.0]
[0.5−0.8)
[0.2−0.5)
[0.0−0.2)
NA
P=3.21e−33
−log10(Observed p)
26
19.5
13
6.5
0
Recombination rate (cM/Mb)
96
80
64
48
32
16
0
OR52J2P OR52E3P OR51A1P HBBP1 OR51AB1P OR51B8P OR51K1P UBQLN3 OR52H2P TRIM6 OR52U1P OR52N5 OR52E8 OR56A3
OR51P1P OR52S1P OR52A1 HBD OR51B2 HBE1 OR51Q1 OR52D1 OR52H1 OR52B6 TRIM22 OR56B2P OR52E6 OR52Q1P OR56A4
OR51L1 OR52E1 OR52A5 HBB OR51B3P HBG2 OR51J1 OR51A10P OR52V1P HNRNPA1P53 OR52N4 OR52N2 OR52E5 KRT18P58
MMP26 OR52E2 OR52A4P OR51V1 HBG1 OR51B5 OR51I2 OLFM5P OR52T1P TRIM34 OR56B1 OR52N3P OR52E4 OR52L1
OR52J3 OR52J1P OR52Z1P BGLT3 OR51B4 OR51B6 OR51I1 UBQLNL OR52B5P TRIM6−TRIM34 OR52P1 OR52N1 OR52E7P OR56A5
4,988,319
5,242,308
5,496,296
5,750,284
6,004,273
Chromosome 11 (bp)

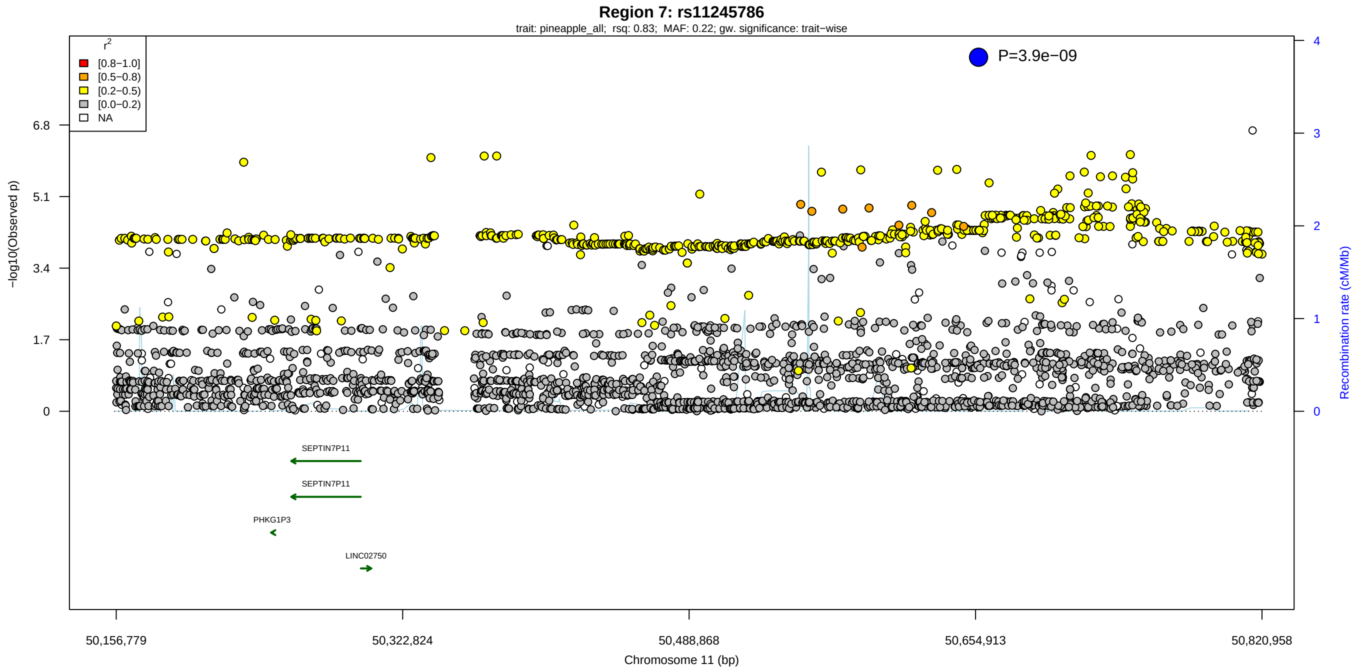
Region 7: rs11245786
trait: pineapple_all; rsq: 0.83; MAF: 0.22; gw. significance: trait−wise
r2
[0.8−1.0]
[0.5−0.8]
[0.2−0.5]
[0.0−0.2]
NA
P=3.9e−09
−log10(Observed p)
Recombination rate (cM/Mb)
SEPTIN7P11
SEPTIN7P11
PHKG1P3
LINC02750
50,156,779
50,322,824
50,488,868
50,654,913
50,820,958
Chromosome 11 (bp)

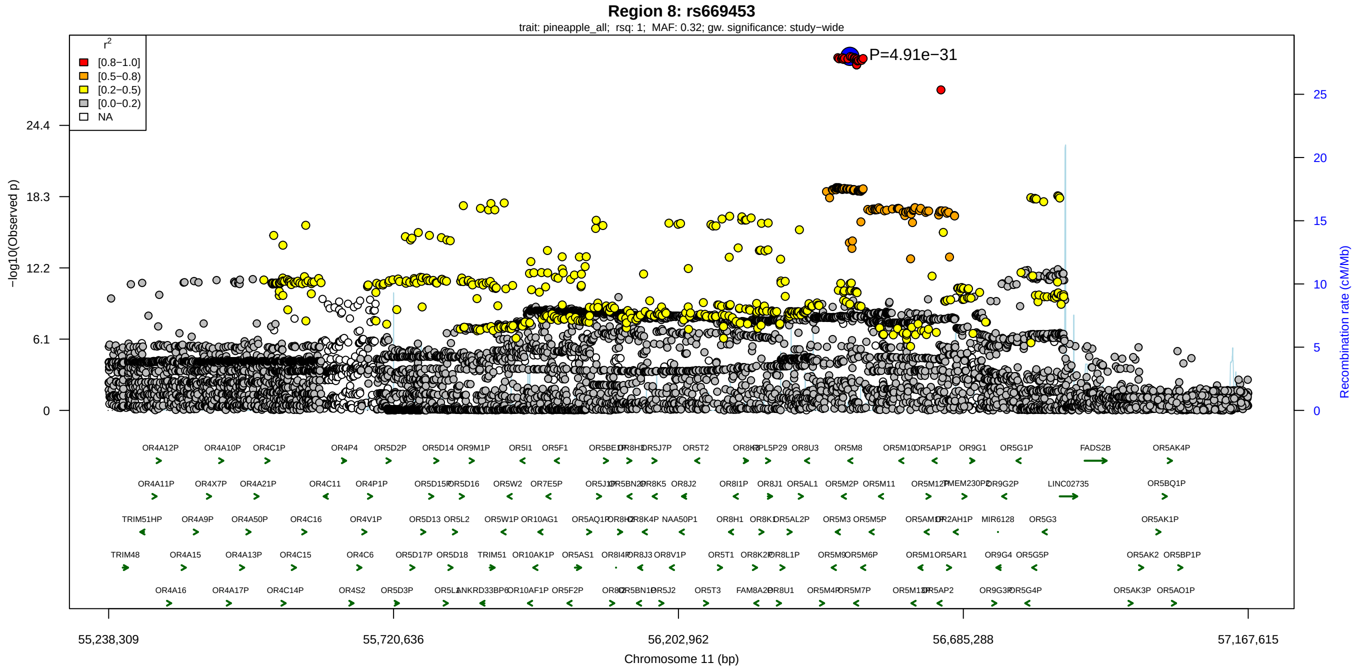
Region 8: rs669453
trait: pineapple_all; rsq: 1; MAF: 0.32; gw. significance: study−wide
r2
[0.8−1.0]
[0.5−0.8]
[0.2−0.5]
[0.0−0.2]
NA
P=4.91e−31
−log10(Observed p)
Recombination rate (cM/Mb)
OR4A12P OR4A10P OR4C1P OR4P4 OR5D2P OR5D14 OR9M1P OR5I1 OR5F1 OR5T2 OR8U3 OR5M8 OR5AP1P OR9G1 OR5G1P FADS2B OR5AK4P
OR4A11P OR4X7P OR4A21P OR4C11 OR4P1P OR5D16 OR5W2 OR7E5P OR8K5 OR8J2 OR8I1P OR8J1 OR5AL1 OR5M2P OR5M11 OR9G2P LINC02735 OR5BQ1P
TRIM51HP OR4A9P OR4A50P OR4C16 OR4V1P OR5D13 OR5L2 OR10AG1 OR8K4P NAA50P1 OR8H1 OR5AL2P OR5M3 OR5M5P MIR6128 OR5G3 OR5AK1P
TRIM48 OR4A15 OR4A13P OR4C15 OR4C6 OR5D17P OR5D18 TRIM51 OR10AK1P OR5AS1 OR8V1P OR5T1 OR8L1P OR5M6P OR5AR1 OR9G4 OR5G5P OR5AK2 OR5BP1P
OR4A16 OR4A17P OR4C14P OR4S2 OR5D3P OR10AF1P OR5F2P OR5J2 OR5T3 OR8U1 OR5M7P OR5AP2 OR5G4P OR5AK3P OR5AO1P
55,238,309
55,720,636
56,202,962
56,685,288
57,167,615
Chromosome 11 (bp)

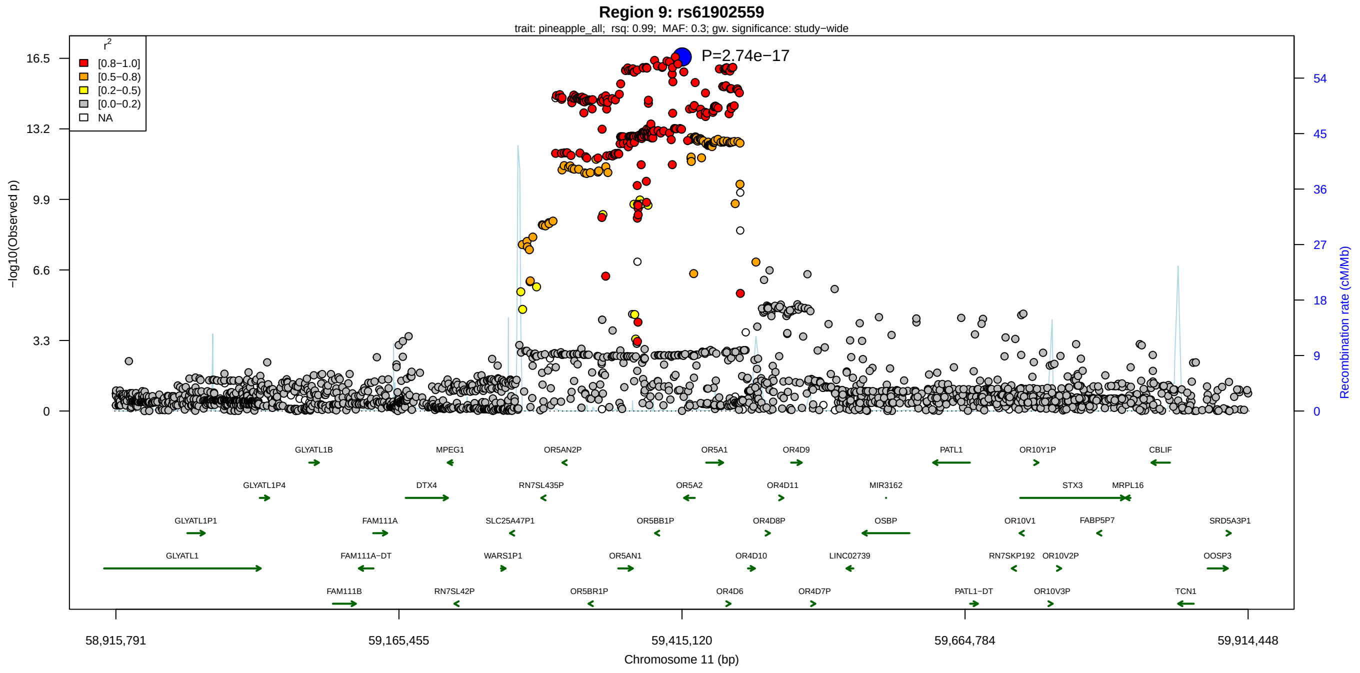
Region 9: rs61902559
trait: pineapple_all; rsq: 0.99; MAF: 0.3; gw. significance: study−wide
r2
[0.8−1.0]
[0.5−0.8]
[0.2−0.5]
[0.0−0.2]
NA
P=2.74e−17
−log10(Observed p)
Recombination rate (cM/Mb)
GLYATL1B MPEG1 OR5AN2P OR5A1 OR4D9 PATL1 OR10Y1P CBLIF
GLYATL1P4 DTX4 RN7SL435P OR5A2 OR4D11 MIR3162 STX3 MRPL16
GLYATL1P1 FAM111A SLC25A47P1 OR5B3P OR4D8P OSBP OR10V1 FABP5P7 SRD5A3P1
GLYATL1 FAM111A−DT WARS1P1 OR5AN1 OR4D10 LINC02739 RN7SKP192 OR10V2P OOSP3
FAM111B RN7SL42P OR5BR1P OR4D6 OR4D7P PATL1−DT OR10V3P TCN1
58,915,791
59,165,455
59,415,120
59,664,784
59,914,448
Chromosome 11 (bp)

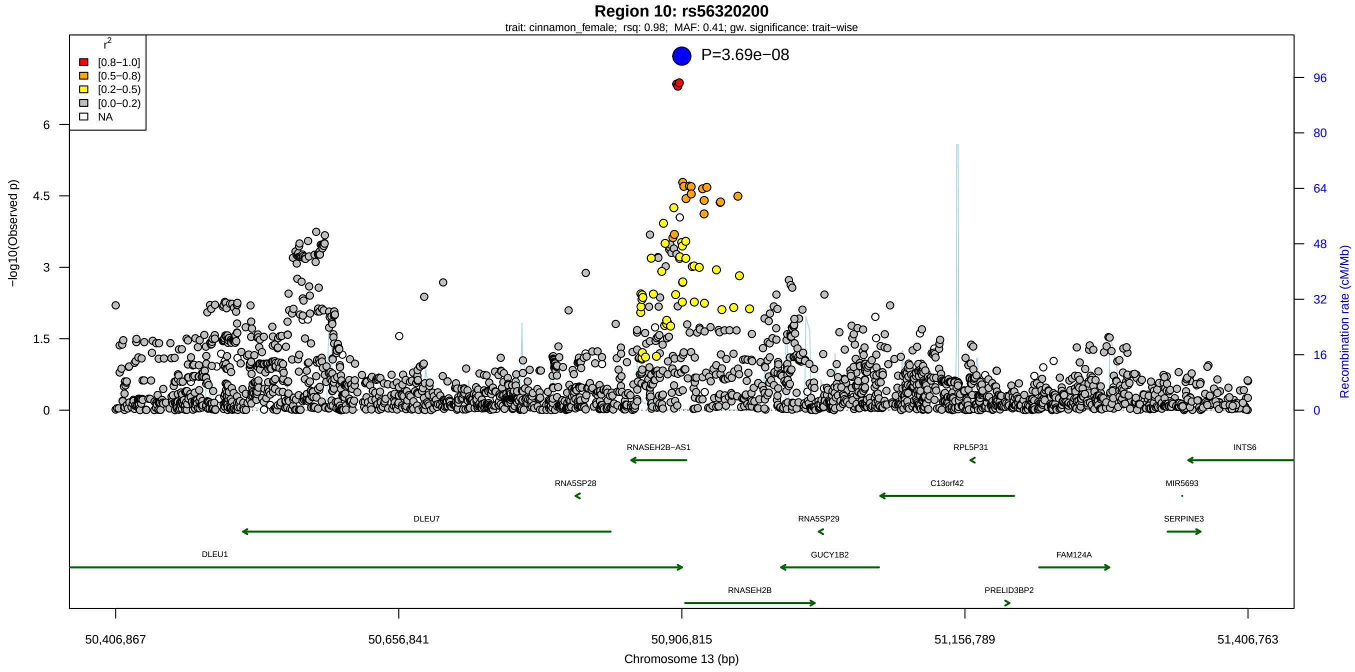

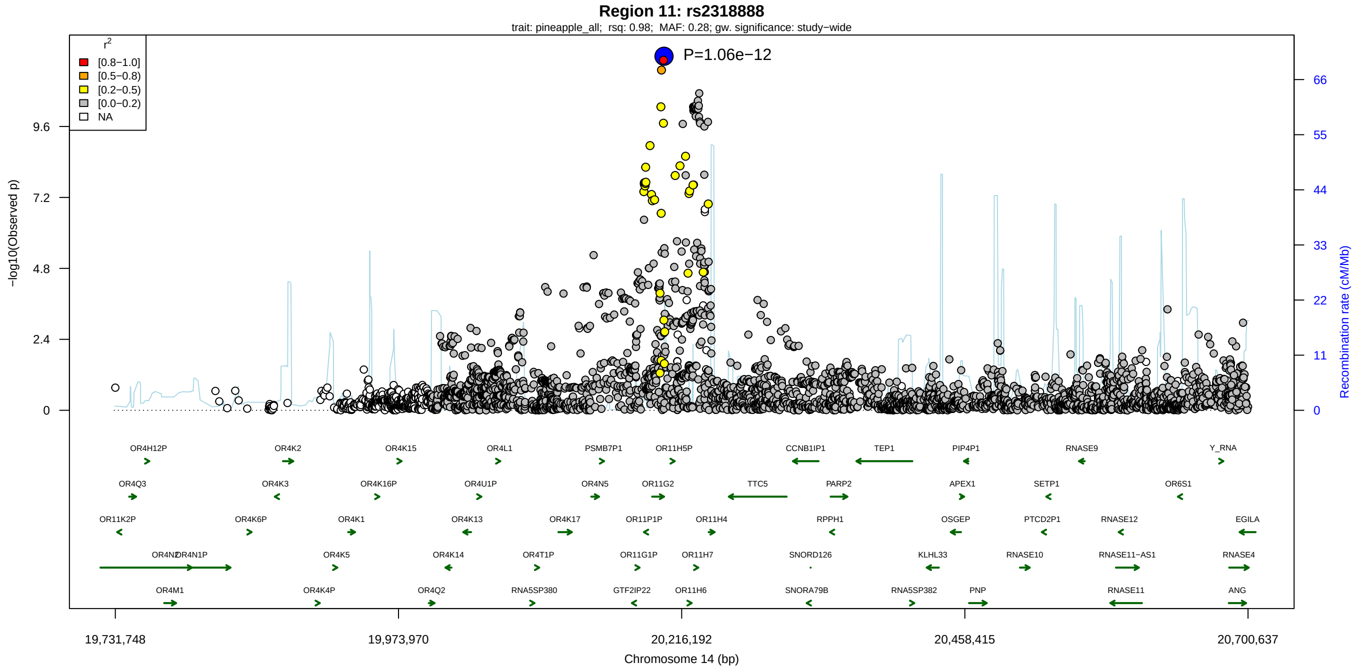

Supplementary Figure 3: Regional association plots of identified loci with genome-wide significance. The header entry 'gw. significance' represents the significance level of the index variant in a two-sided test for association: study-wide - $p < 3.85 \times 10^{-9}$, trait-wise - $p < 5 \times 10^{-8}$. Strength of LD with the index variant is represented by colour. Data is shown for the best associated phenotype for each locus. For region 7, LD was calculated by manually specifying all pairs containing the index variant to improve accuracy.

rs73252922

Locus: 1, phenotype: fish all, I2: 45.3, gw. sig.: trait−wise

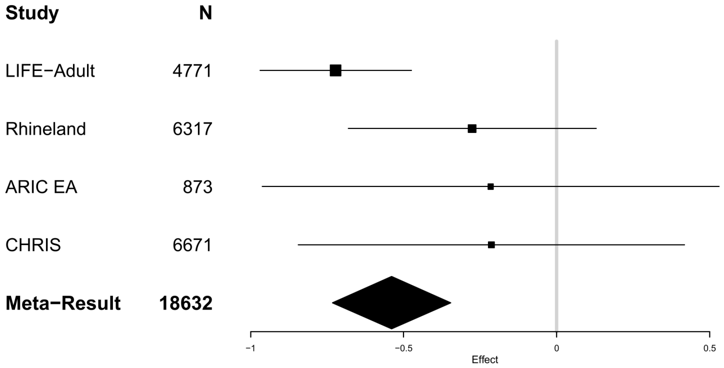

rs116058752

Locus: 2, phenotype: orange female, I2: 0.1, gw. sig.: study−wide

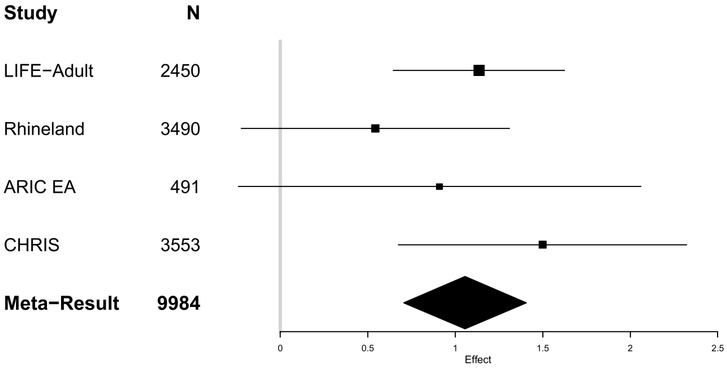

rs17161232

Locus: 3, phenotype: lemon all, I2: 0, gw. sig.: trait−wise

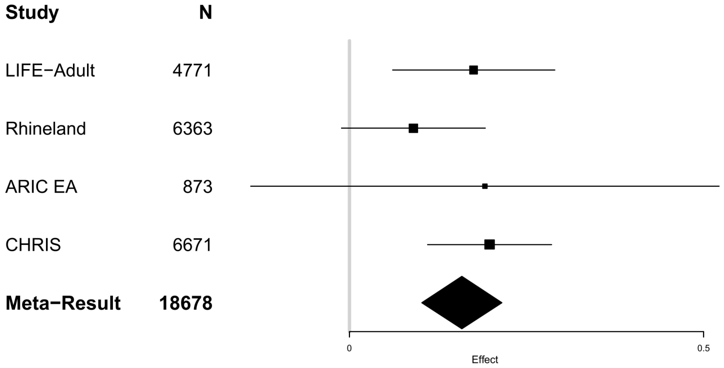

rs3117345

Locus: 4, phenotype: cinnamon all, I2: 18.9, gw. sig.: study−wide

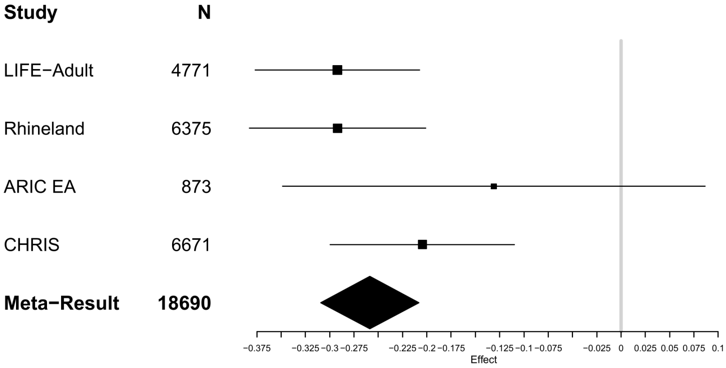

rs3117345

Locus: 4, phenotype: cinnamon female, I2: 72.4, gw. sig.: study−wide

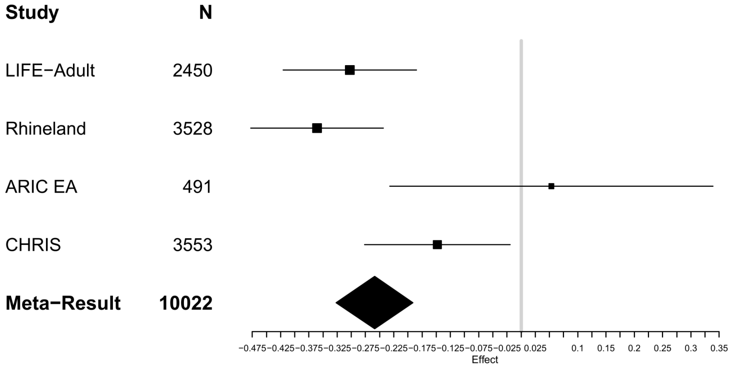

rs3117345

Locus: 4, phenotype: cinnamon male, I2: 3.2, gw. sig.: study−wide

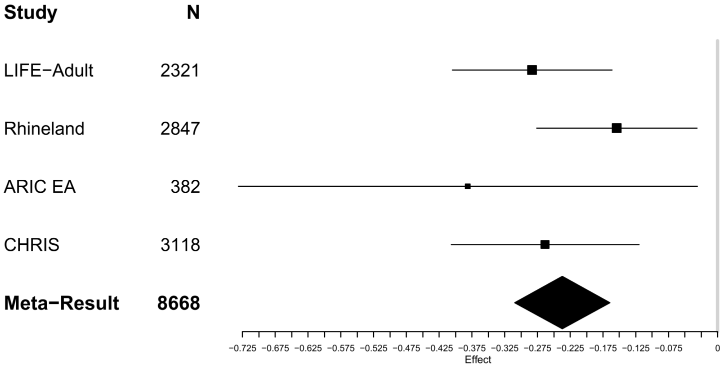

rs41286168

Locus: 5, phenotype: fish all, I2: 77.8, gw. sig.: study−wide

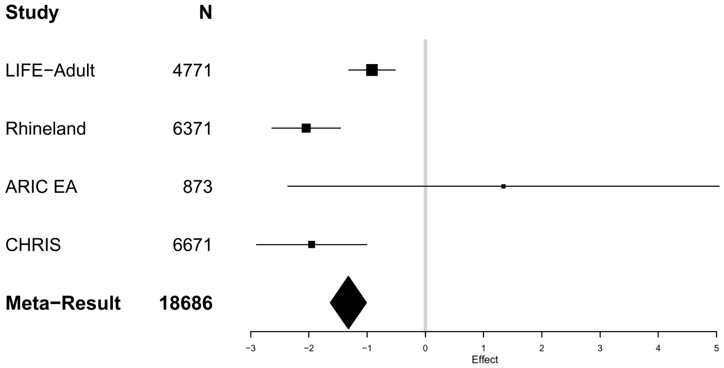

rs317787

Locus: 6, phenotype: cinnamon all, I2: 33.3, gw. sig.: study−wide

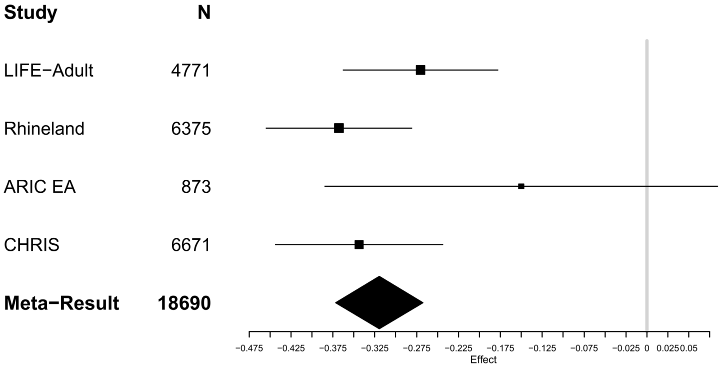

rs317787

Locus: 6, phenotype: cinnamon female, I2: 0, gw. sig.: study−wide

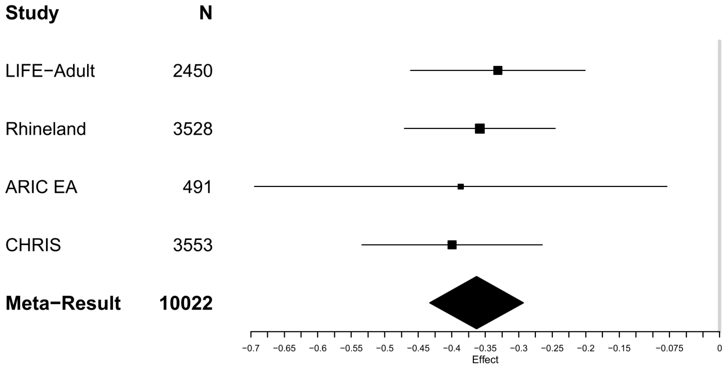

rs317787

Locus: 6, phenotype: cinnamon male, I2: 62.3, gw. sig.: study−wide

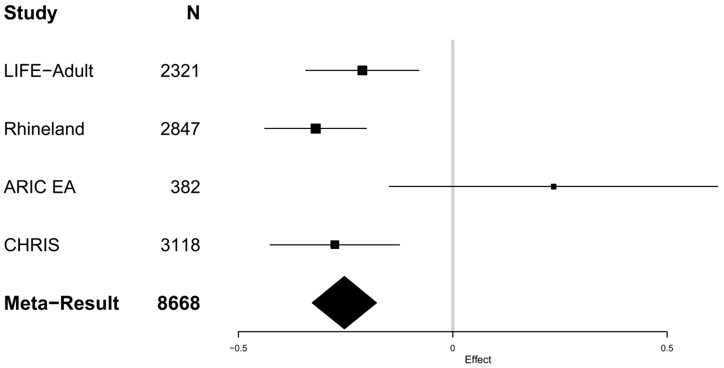

rs11245786

Locus: 7, phenotype: pineapple all, I2: 44, gw. sig.: trait−wise

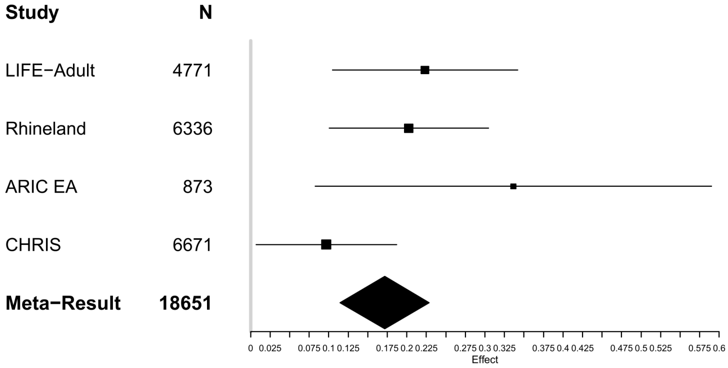

rs669453

Locus: 8, phenotype: pineapple all, I2: 72.2, gw. sig.: study−wide

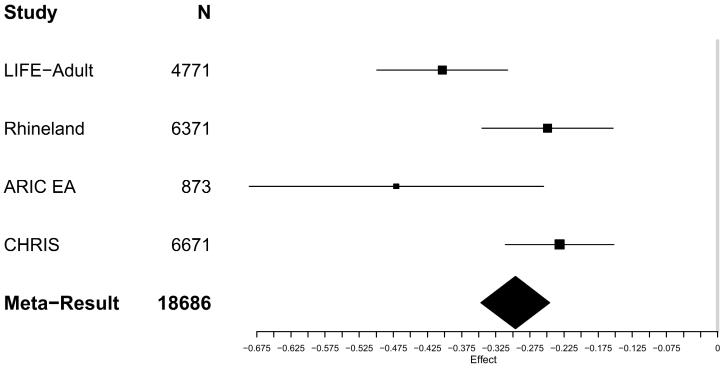

rs669453

Locus: 8, phenotype: pineapple female, I2: 51.3, gw. sig.: study−wide

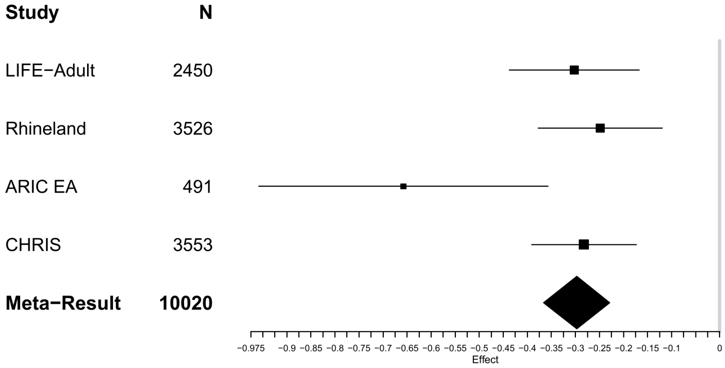

rs669453

Locus: 8, phenotype: pineapple male, I2: 74.9, gw. sig.: study−wide

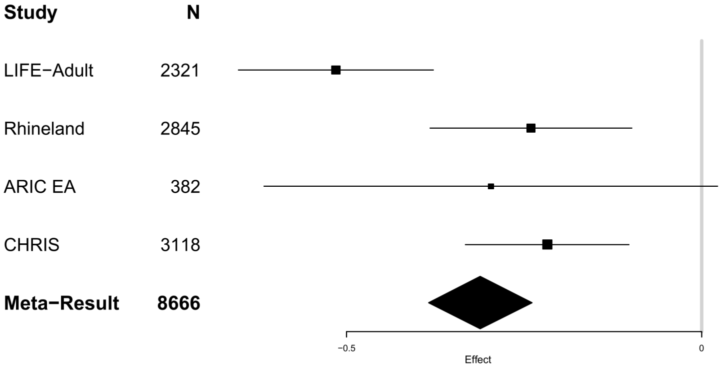

rs669453

Locus: 8, phenotype: score all, I2: 39.1, gw. sig.: trait−wise

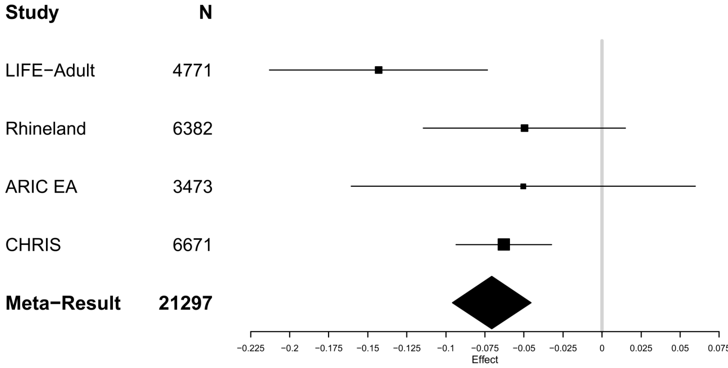

rs61902559

Locus: 9, phenotype: pineapple all, I2: 56.1, gw. sig.: study−wide

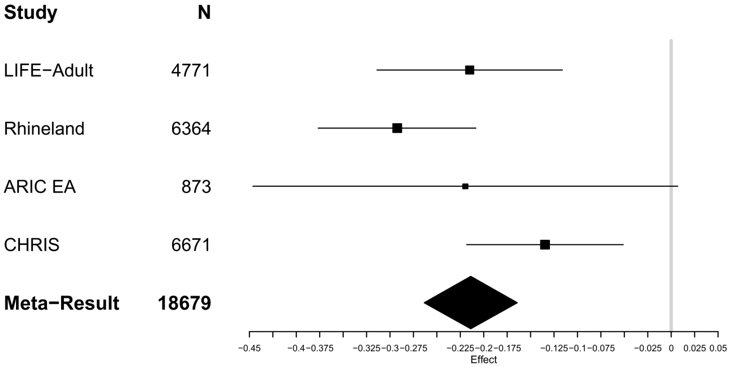

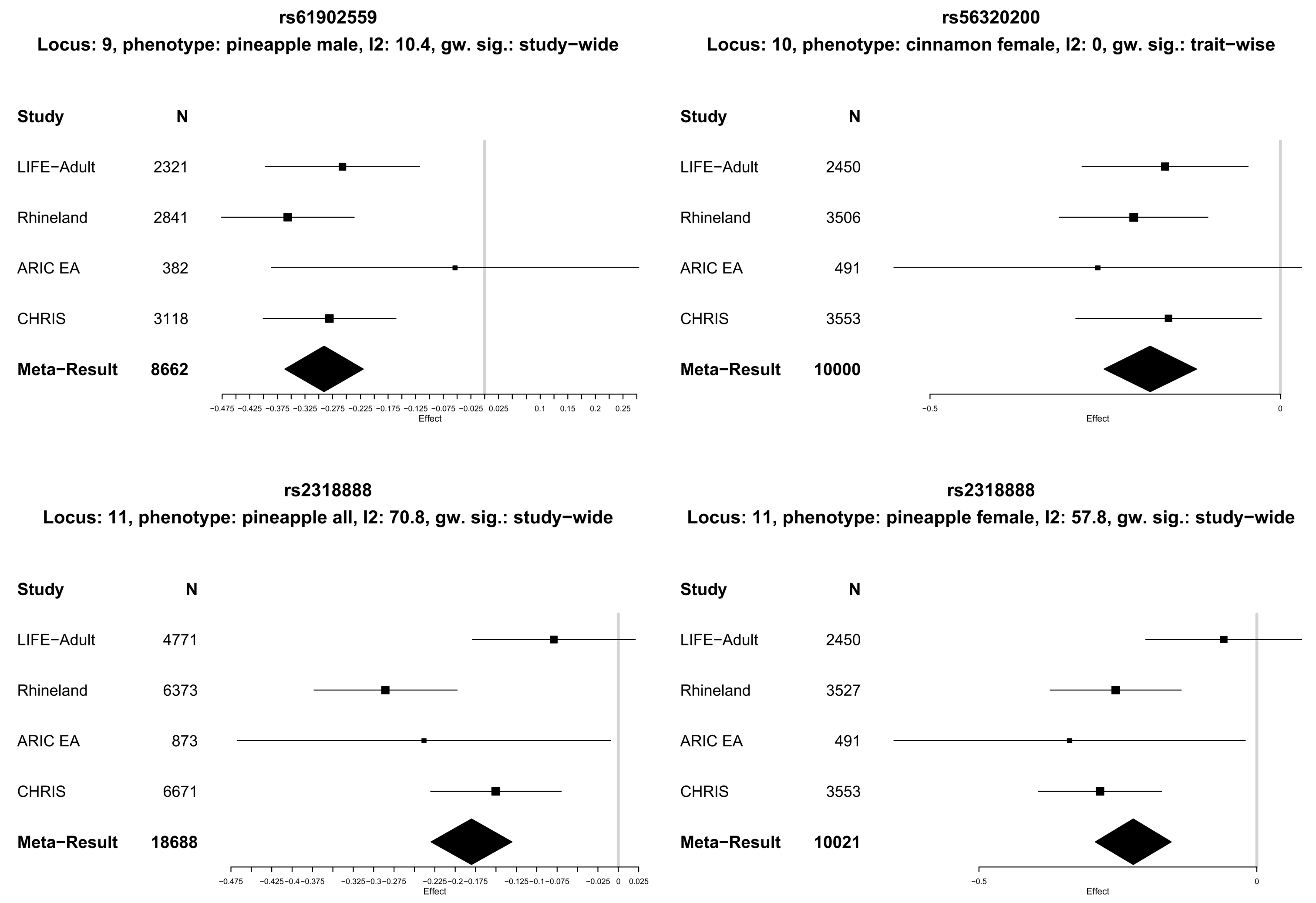

Supplementary Figure 4: Forest plots for index variants of identified loci with genome-wide significance. The header entry 'gw. sig.' represents the significance level of the index variant: study-wide - $p < 3.85 \times 10^{-9}$, trait-wise - $p < 5 \times 10^{-8}$. Data is shown for each trait and analysis group where the locus includes variants with genome-wide significance. Error bars show the 95% confidence interval.

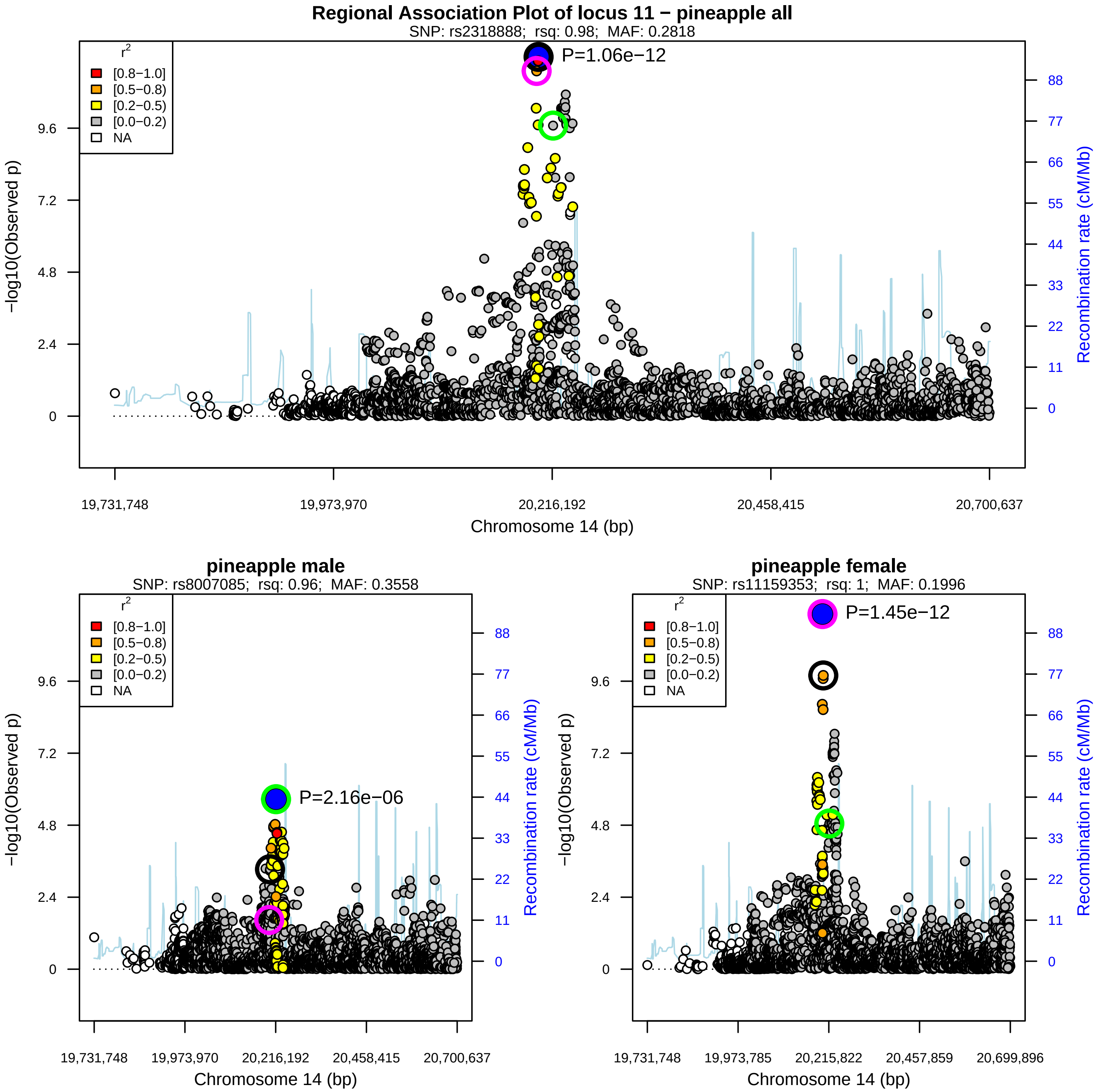

Supplementary Figure 5: Comparison of regional association plots of pineapple at locus 11 between analysis groups overall, males and females. The same genomic region is displayed for all analysis groups. The y-axes report the (negative) log10(p-values) (two-sided) of the associations (β coefficient of additive genetic effect in logistic regression analysis). The primarily identified index variant rs2318888 showed no sex interaction and is in LD with the best associated SNP in females (rs11159353, LD = 0.65 in LDlink). Colocalization analysis supported independent hits for the sexes (PP(H3) = 67%). Subgroup-specific top hits are depicted as circles (black = overall, green = male, magenta = female). Strength of LD with the respective index variant is indicated by colour shading.

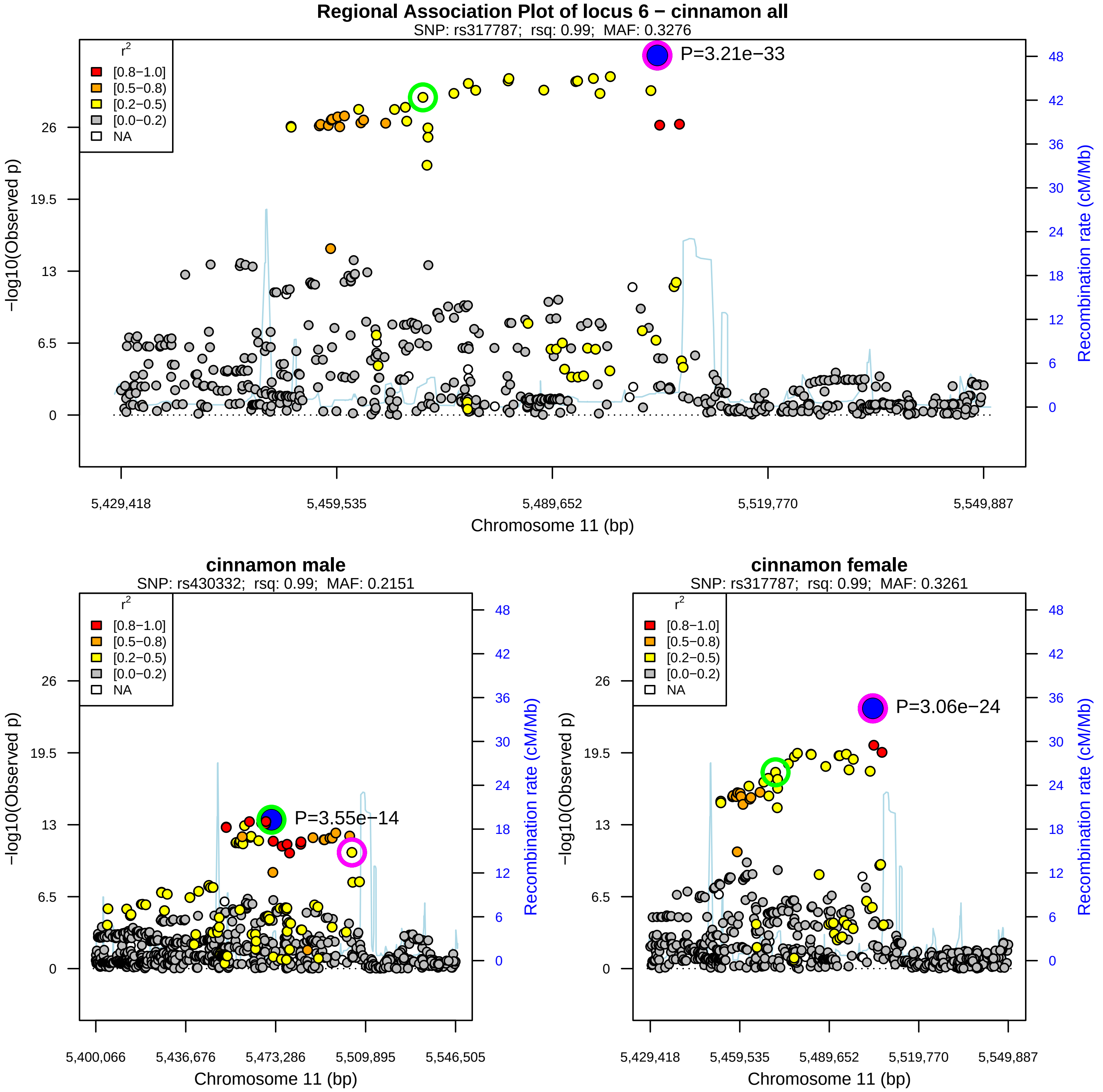

Supplementary Figure 6: Comparison of regional association plots of cinnamon at locus 6 between analysis groups overall, males and females. The same genomic region is displayed for overall, female, and male analysis, respectively. The y-axes report the (negative) log10(p-values) (two-sided) of the associations (β coefficient of additive genetic effect in linear regression analysis). Subgroup-specific top hits are depicted as circles (green = male, magenta = overall/female). Strength of LD with the respective index variant is indicated by colour shading.

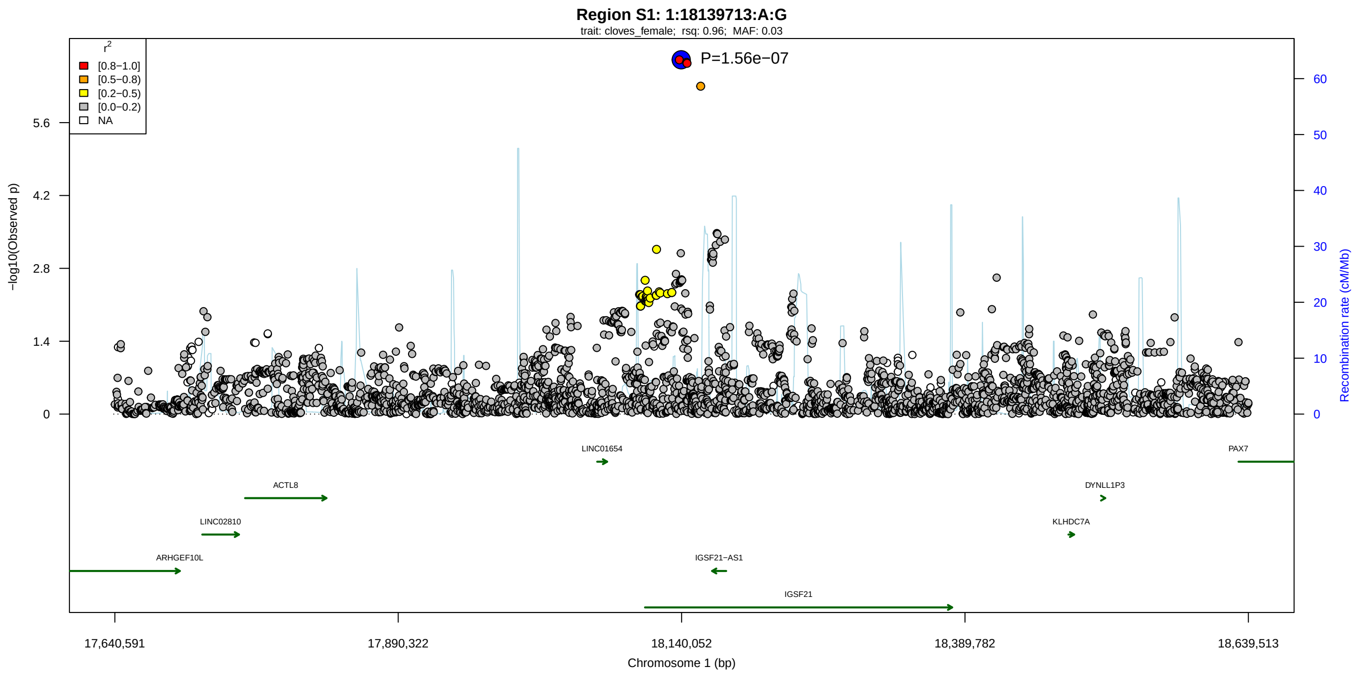
Region S1: 1:18139713:A:G
trait: cloves_female; rsq: 0.96; MAF: 0.03
r2
[0.8−1.0]
[0.5−0.8]
[0.2−0.5]
[0.0−0.2]
NA
P=1.56e−07
−log10(Observed p)
5.6
4.2
2.8
1.4
0
60
50
40
30
20
10
0
Recombination rate (cM/Mb)
LINC01654
PAX7
ACTL8
DYNLL1P3
LINC02810
KLHDC7A
ARHGEF10L
IGSF21−AS1
IGSF21
17,640,591
17,890,322
18,140,052
18,389,782
18,639,513
Chromosome 1 (bp)

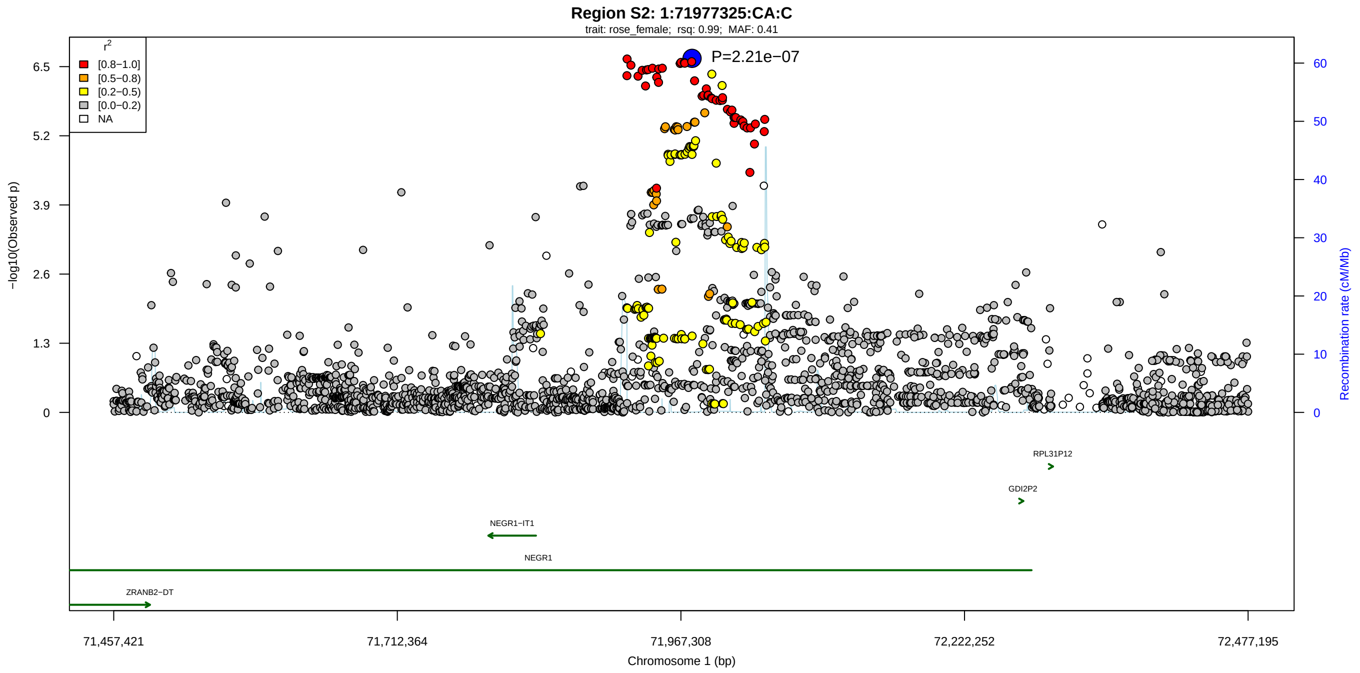
Region S2: 1:71977325:CA:C
trait: rose_female; rsq: 0.99; MAF: 0.41
r2
[0.8−1.0]
[0.5−0.8]
[0.2−0.5]
[0.0−0.2]
NA
P=2.21e−07
−log10(Observed p)
6.5
5.2
3.9
2.6
1.3
0
60
50
40
30
20
10
0
Recombination rate (cM/Mb)
RPL31P12
GDI2P2
NEGR1−IT1
NEGR1
ZRANB2−DT
71,457,421
71,712,364
71,967,308
72,222,252
72,477,195
Chromosome 1 (bp)

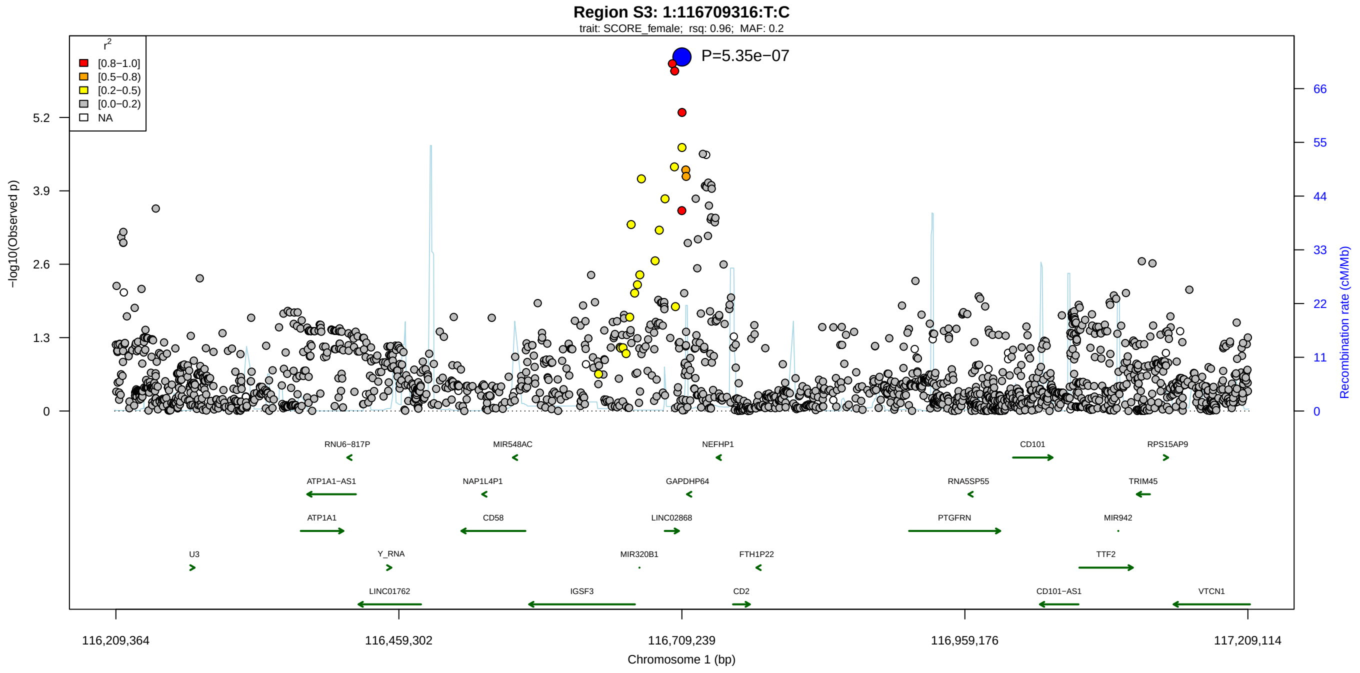
Region S3: 1:116709316:T:C
trait: SCORE_female; rsq: 0.96; MAF: 0.2
r2
[0.8−1.0]
[0.5−0.8]
[0.2−0.5]
[0.0−0.2]
NA
P=5.35e−07
−log10(Observed p)
5.2
3.9
2.6
1.3
0
66
55
44
33
22
11
0
Recombination rate (cM/Mb)
RNU6−817P
MIR548AC
NEFHP1
CD101
RPS15AP9
ATP1A1−AS1
NAP1L4P1
GAPDHP64
RNA5SP55
TRIM45
ATP1A1
CD58
LINC02868
PTGFRN
MIR942
U3
Y_RNA
MIR320B1
FTH1P22
TTF2
LINC01762
IGSF3
CD2
CD101−AS1
VTCN1
116,209,364
116,459,302
116,709,239
116,959,176
117,209,114
Chromosome 1 (bp)

Region S4: 1:232111616:A:G

trait: coffee_male; rsq: 0.98; MAF: 0.2

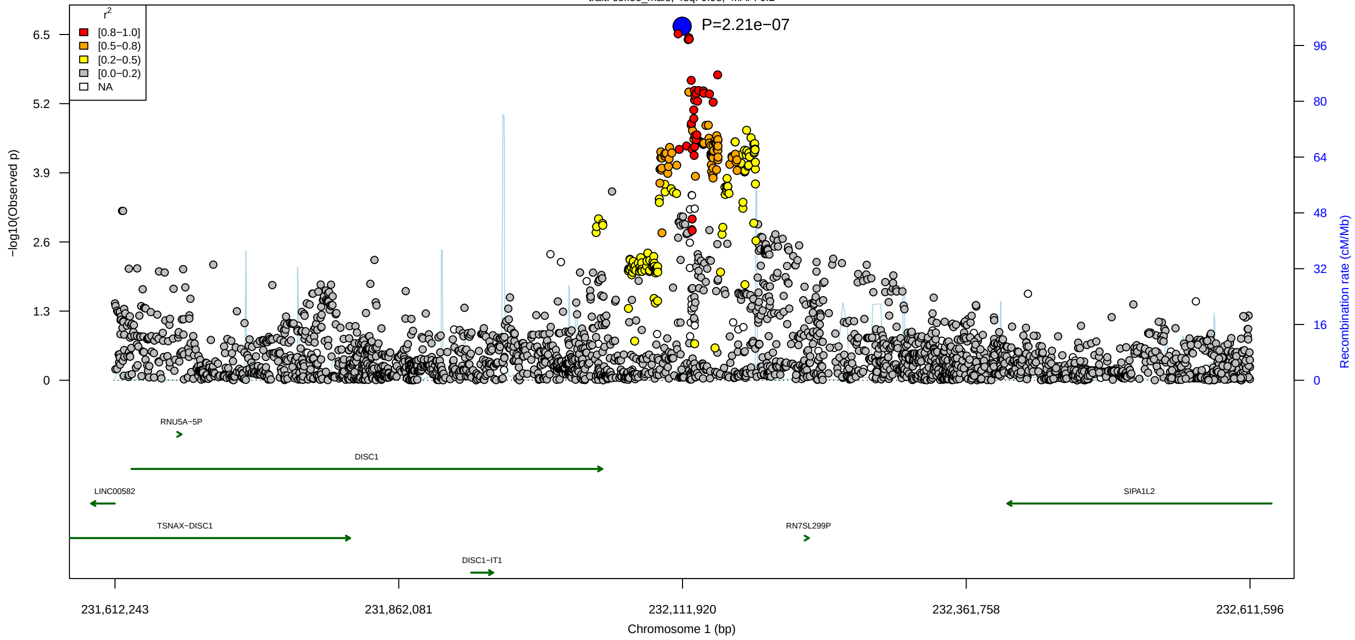

Region S5: 2:24959578:TCCA:T

trait: SCORE_male; rsq: 0.84; MAF: 0.03

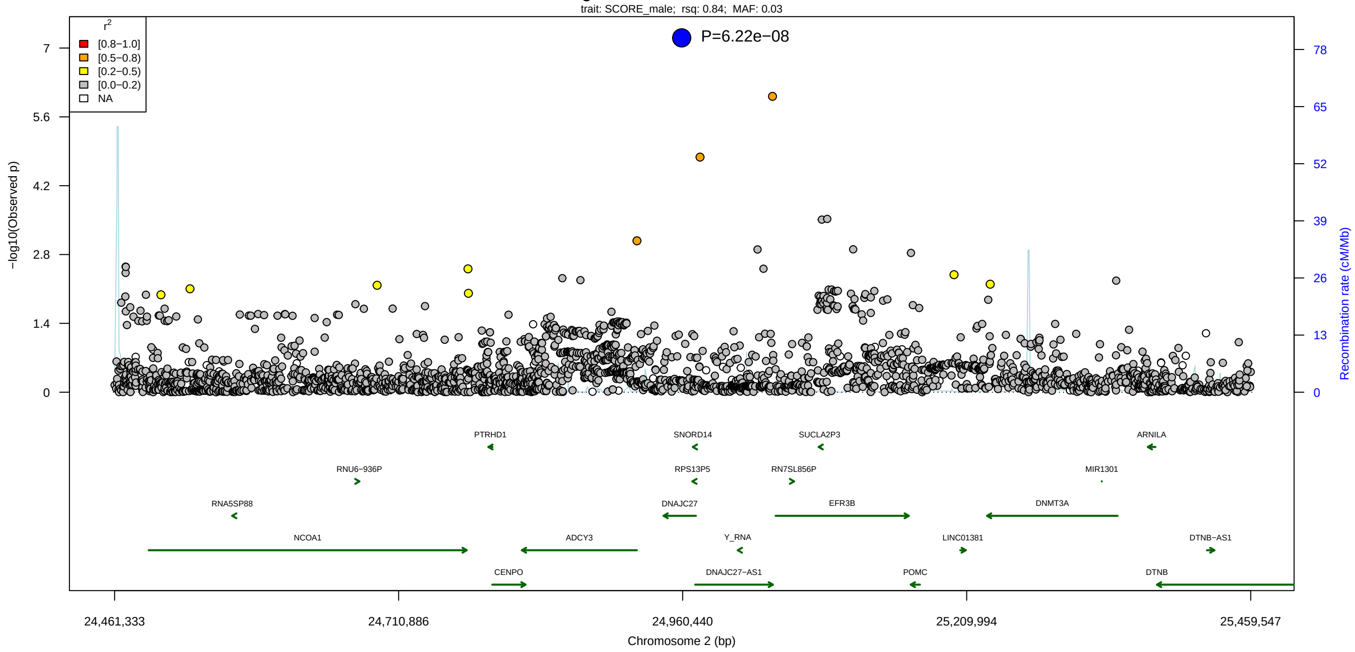

Region S6: 2:40409012:C:T

trait: liquorice_female; rsq: 0.99; MAF: 0.48

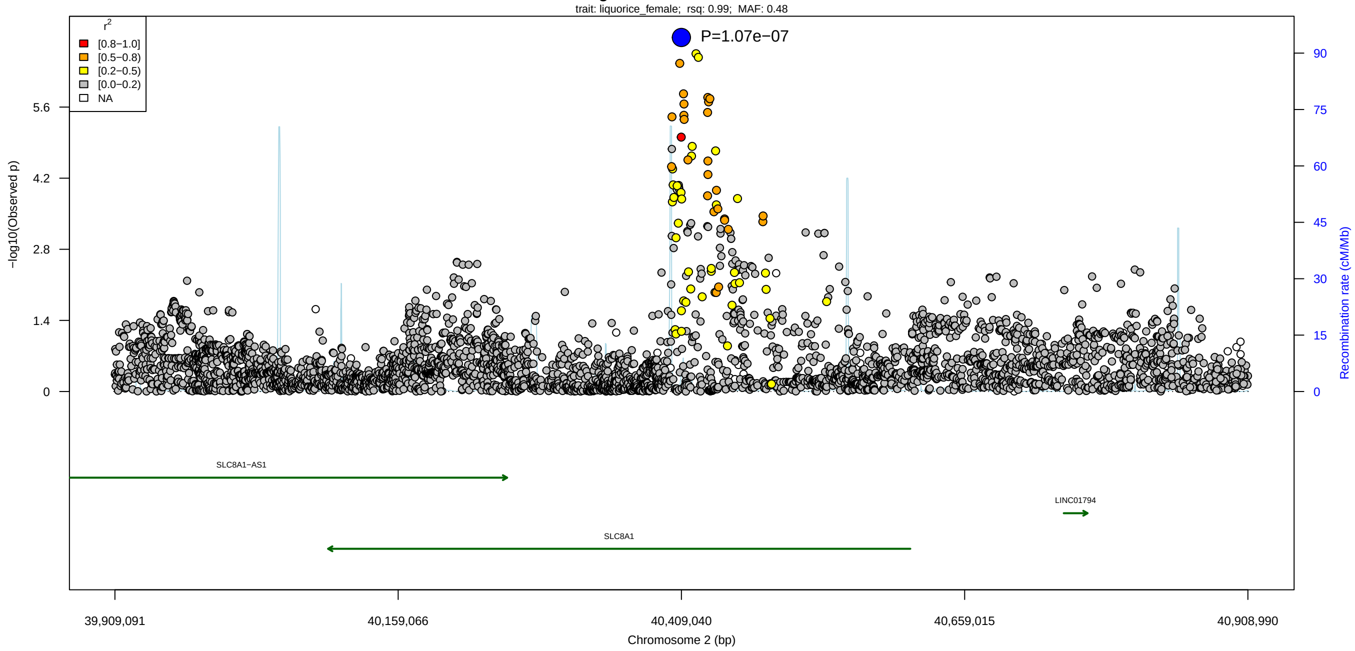

Region S7: 2:187830982:G:A

trait: cinnamon_all; rsq: 0.99; MAF: 0.38

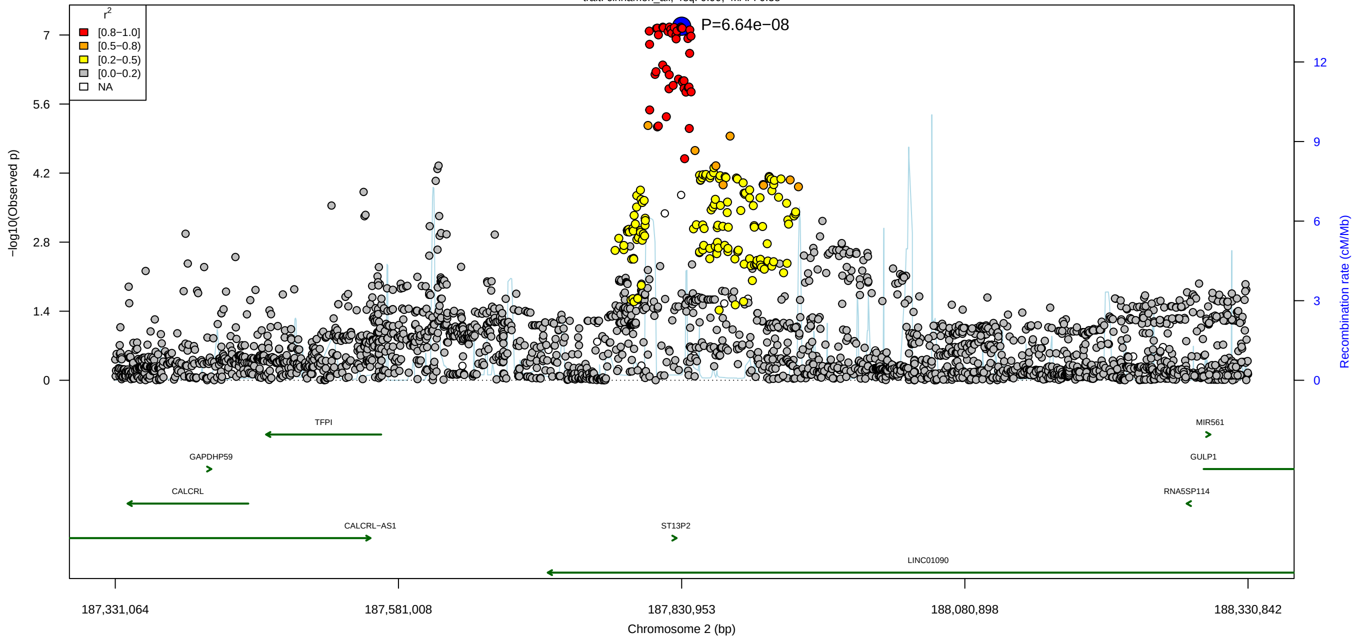

Region S8: 3:159541502:T:C

trait: cinnamon_male; rsq: 0.97; MAF: 0.17

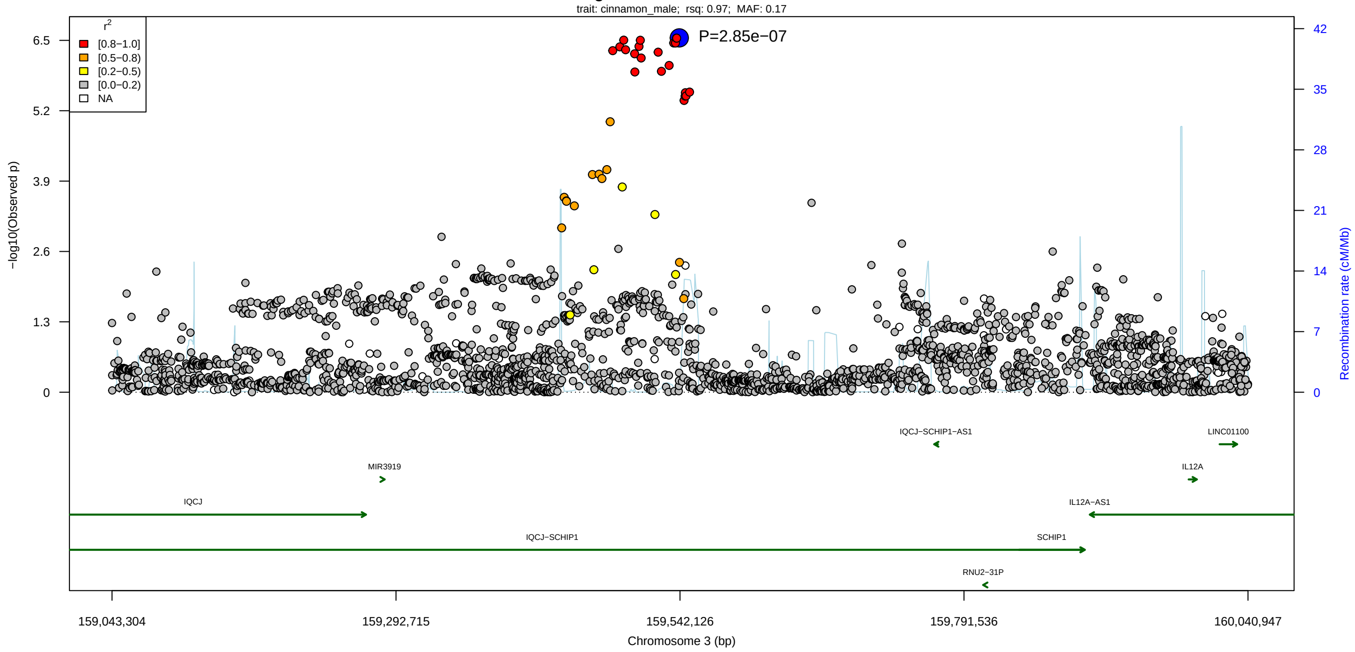

Region S9: 4:104197978:T:A

trait: cinnamon_all; rsq: 0.96; MAF: 0.07

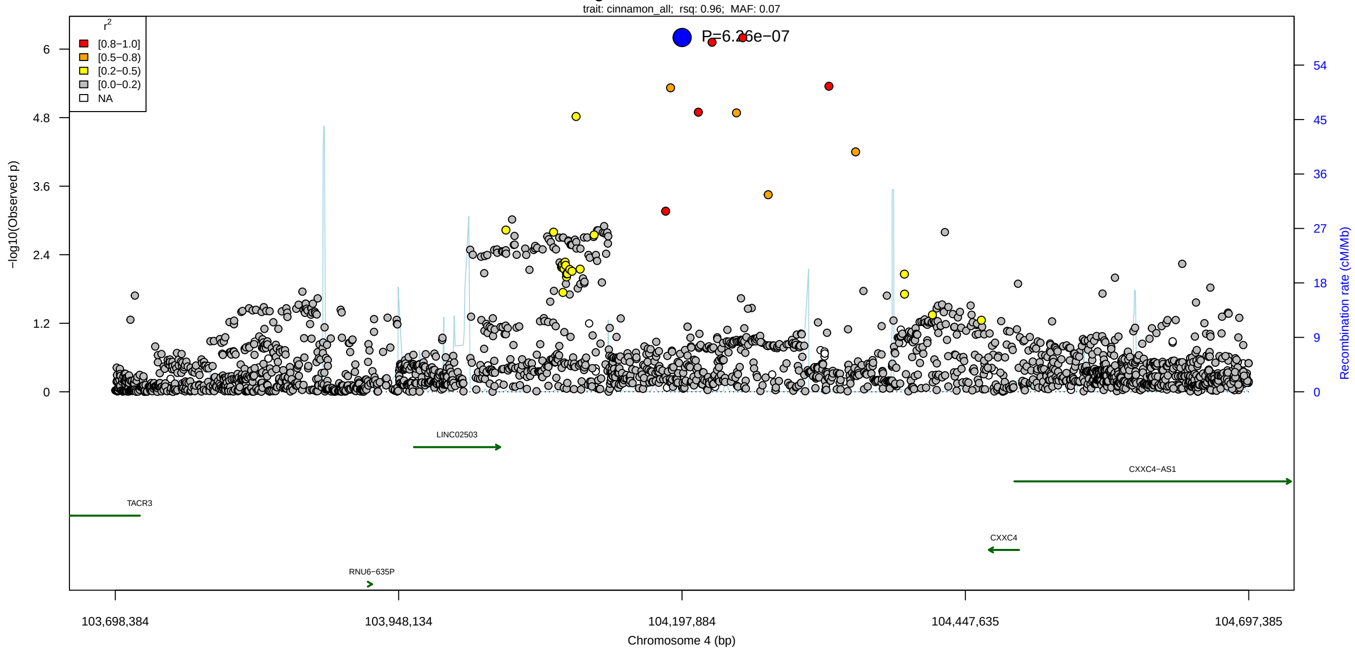

Region S10: 5:11484493:G:A

trait: coffee_male; rsq: 0.97; MAF: 0.01

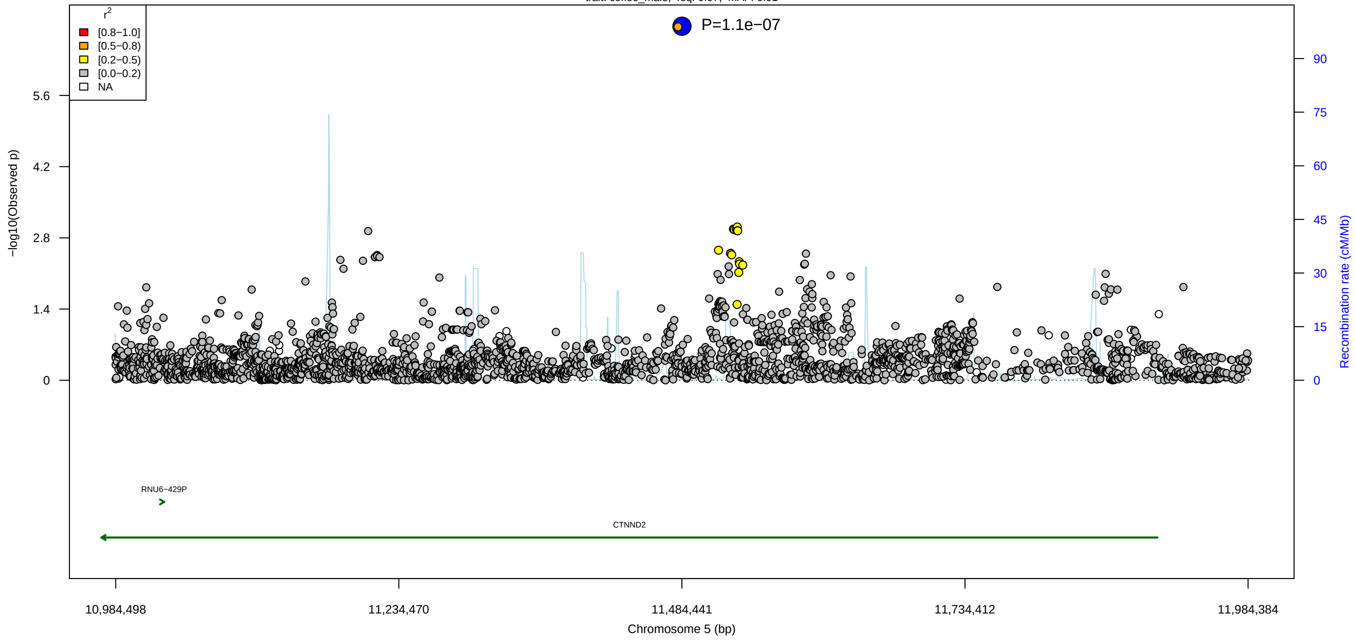

Region S11: 5:93970601:C:CA

trait: cloves_all; rsq: 0.99; MAF: 0.01

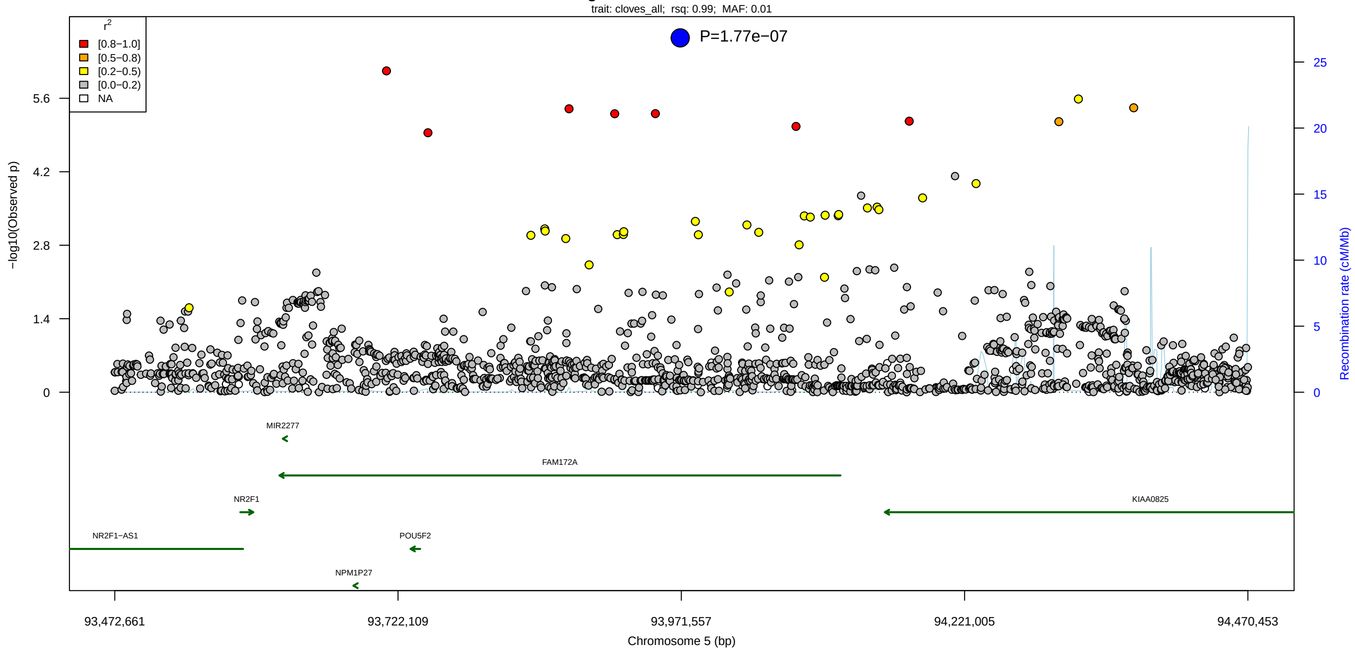

Region S12: 6:115200133:A:G

trait: orange_male; rsq: 0.95; MAF: 0.03

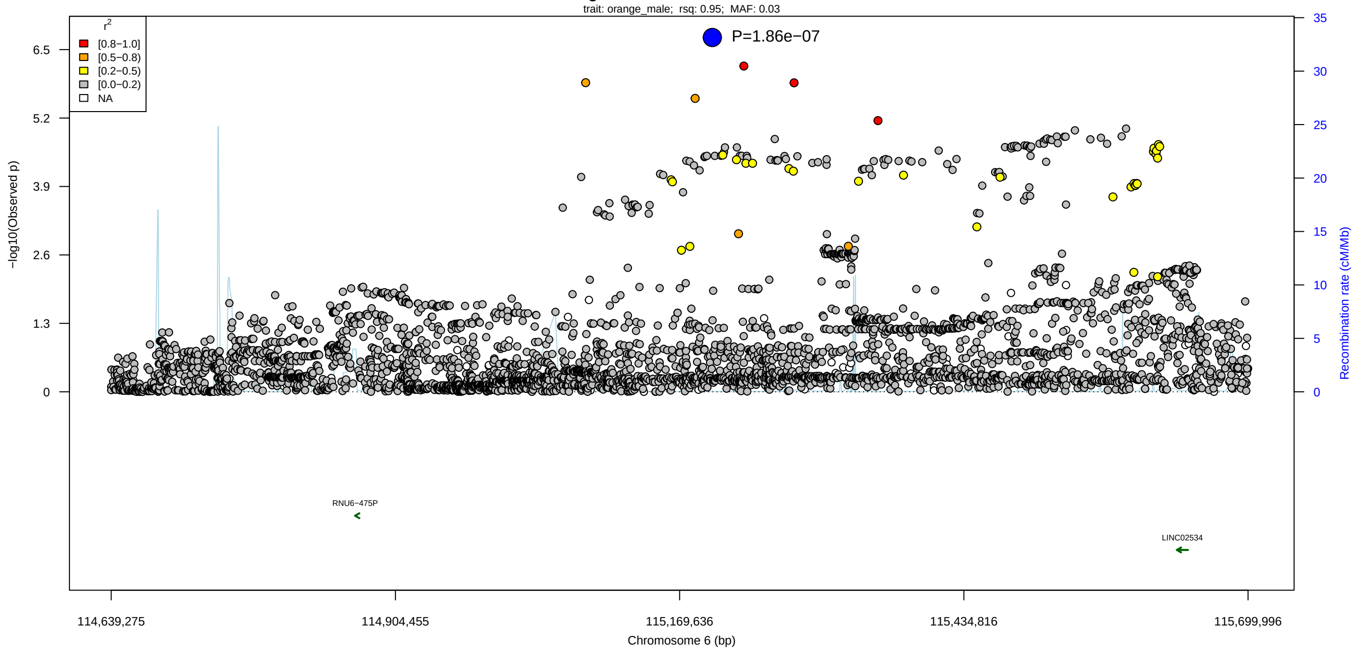

Region S13: 6:123142396:G:A

trait: leather_female; rsq: 0.96; MAF: 0.01

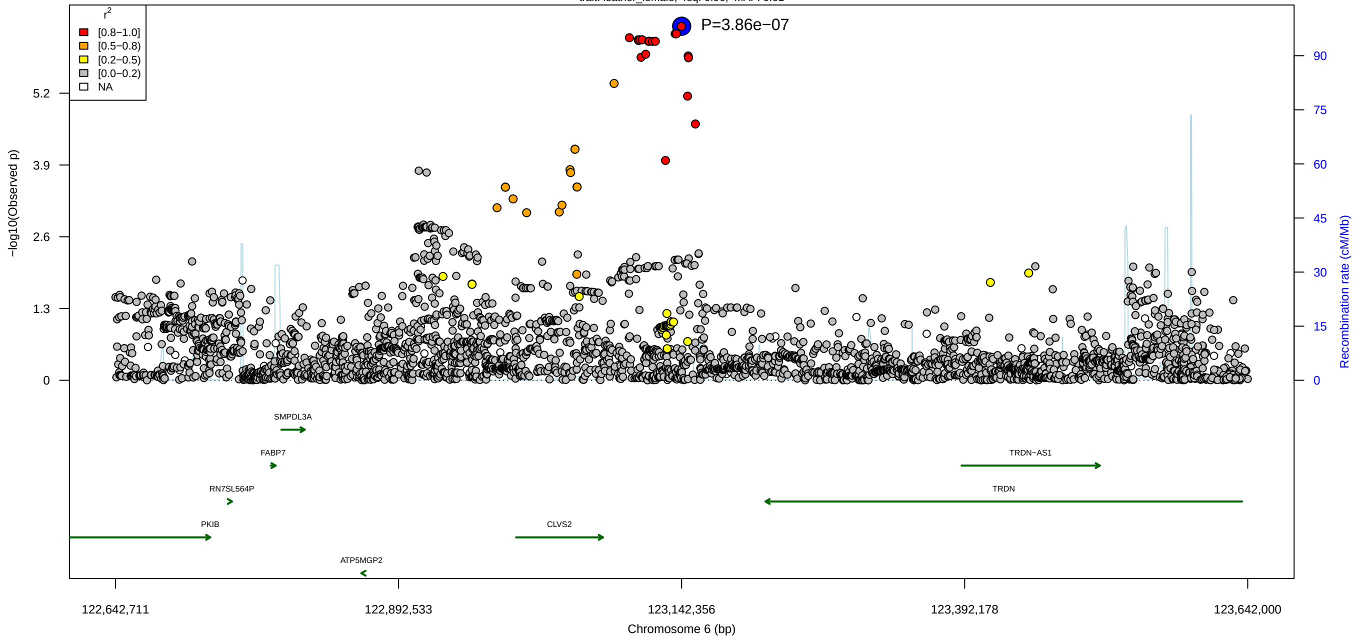

Region S14: 7:144069592:C:T

trait: lemon_all; rsq: 0.98; MAF: 0.15

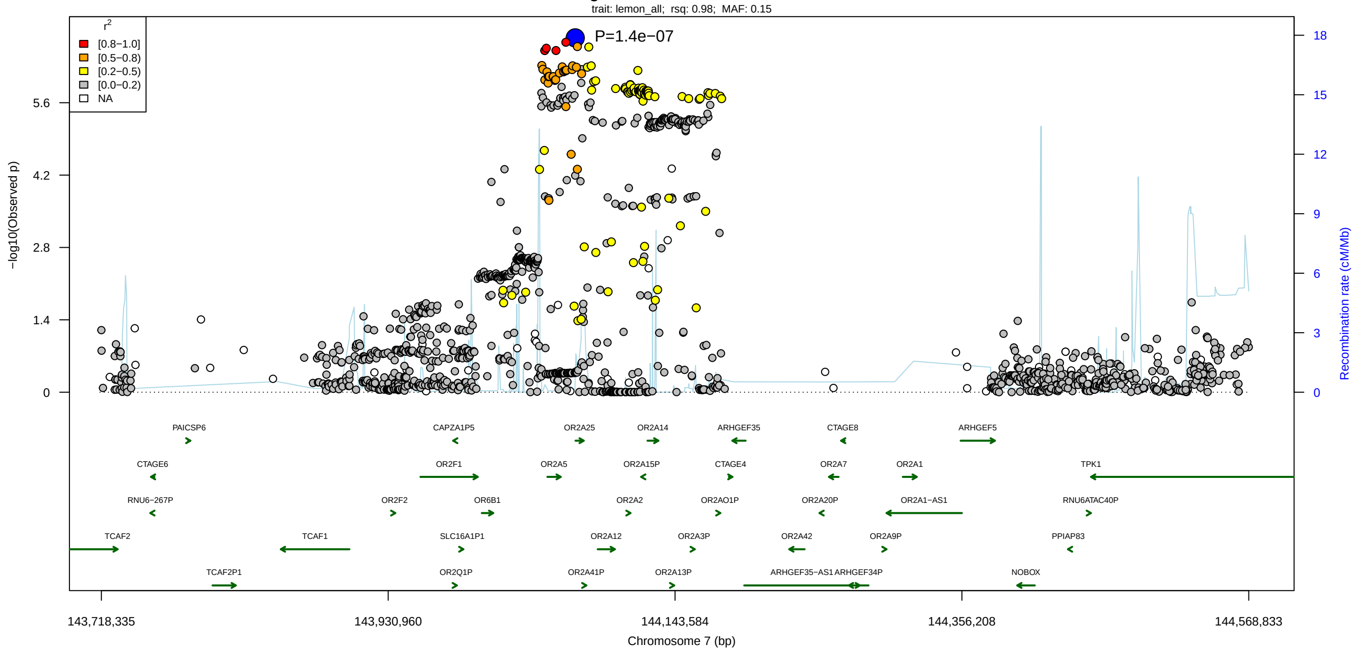

Region S15: 8:9228005:C:A

trait: peppermint_all; rsq: 0.99; MAF: 0.11

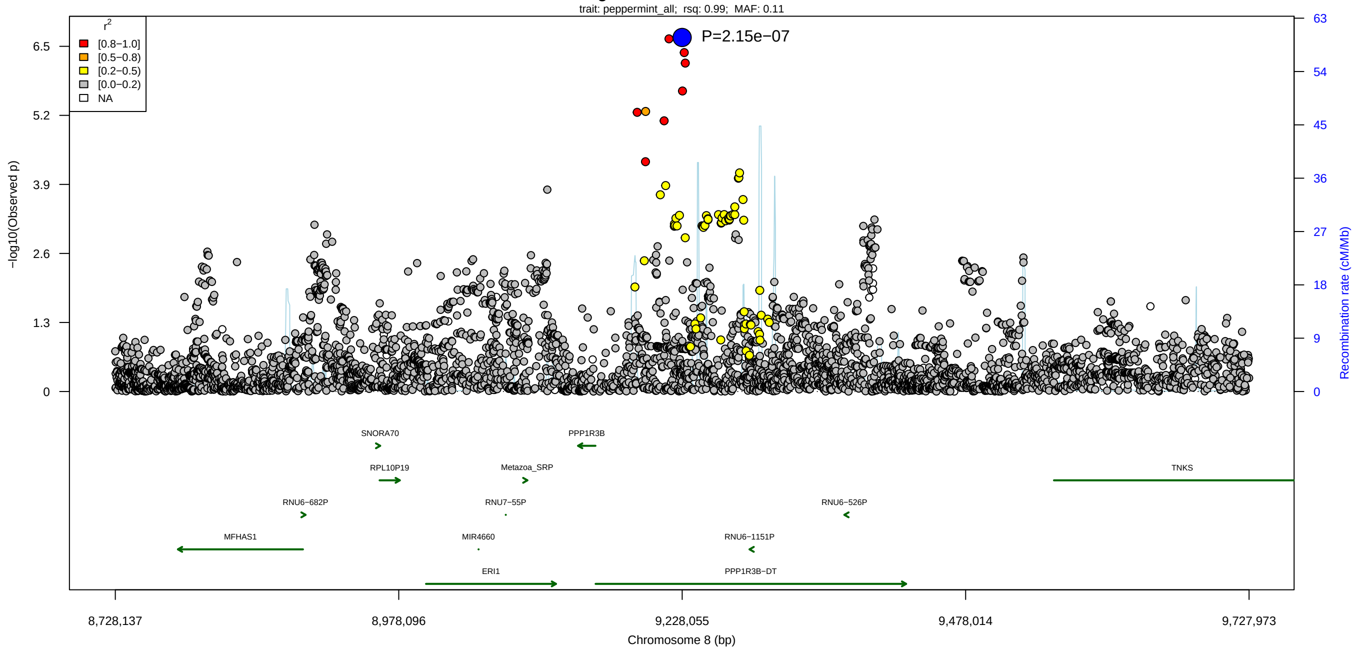

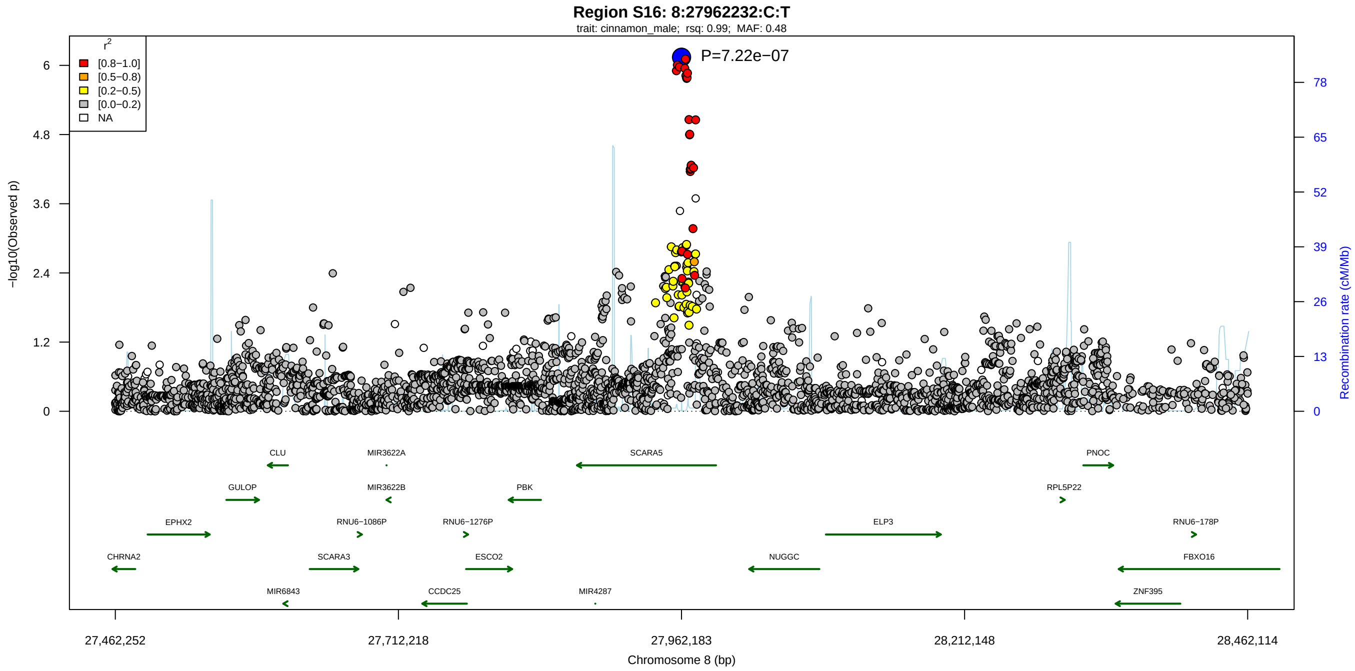
Region S16: 8:27962232:C:T
trait: cinnamon_male; rsq: 0.99; MAF: 0.48
r2
[0.8−1.0]
[0.5−0.8)
[0.2−0.5)
[0.0−0.2)
NA
P=7.22e−07
−log10(Observed p)
Recombination rate (cM/Mb)
CLU
MIR3622A
SCARA5
PNOC
GULOP
MIR3622B
PBK
RPL5P22
EPHX2
RNU6−1086P
RNU6−1276P
ELP3
RNU6−178P
CHRNA2
SCARA3
ESCO2
NUGGC
FBXO16
MIR6843
CCDC25
MIR4287
ZNF395
27,462,252
27,712,218
27,962,183
28,212,148
28,462,114
Chromosome 8 (bp)

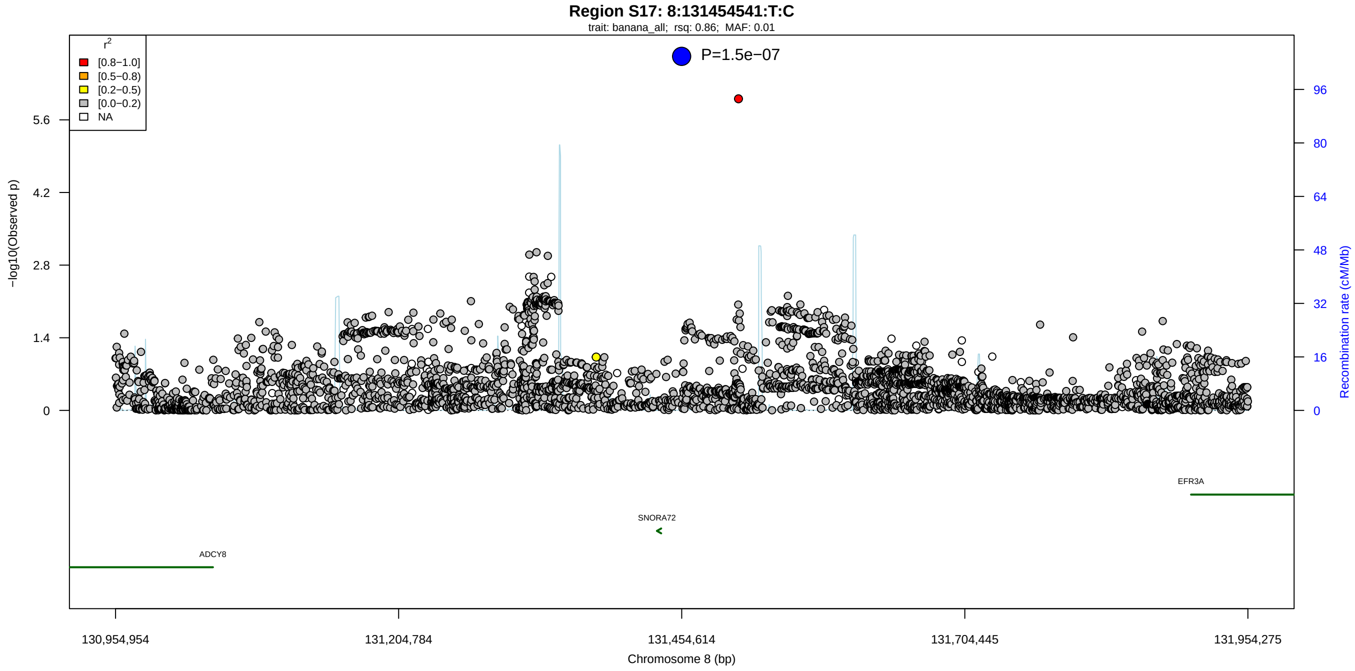
Region S17: 8:131454541:T:C
trait: banana_all; rsq: 0.86; MAF: 0.01
r2
[0.8−1.0]
[0.5−0.8)
[0.2−0.5)
[0.0−0.2)
NA
P=1.5e−07
−log10(Observed p)
Recombination rate (cM/Mb)
EFR3A
SNORA72
ADCY8
130,954,954
131,204,784
131,454,614
131,704,445
131,954,275
Chromosome 8 (bp)

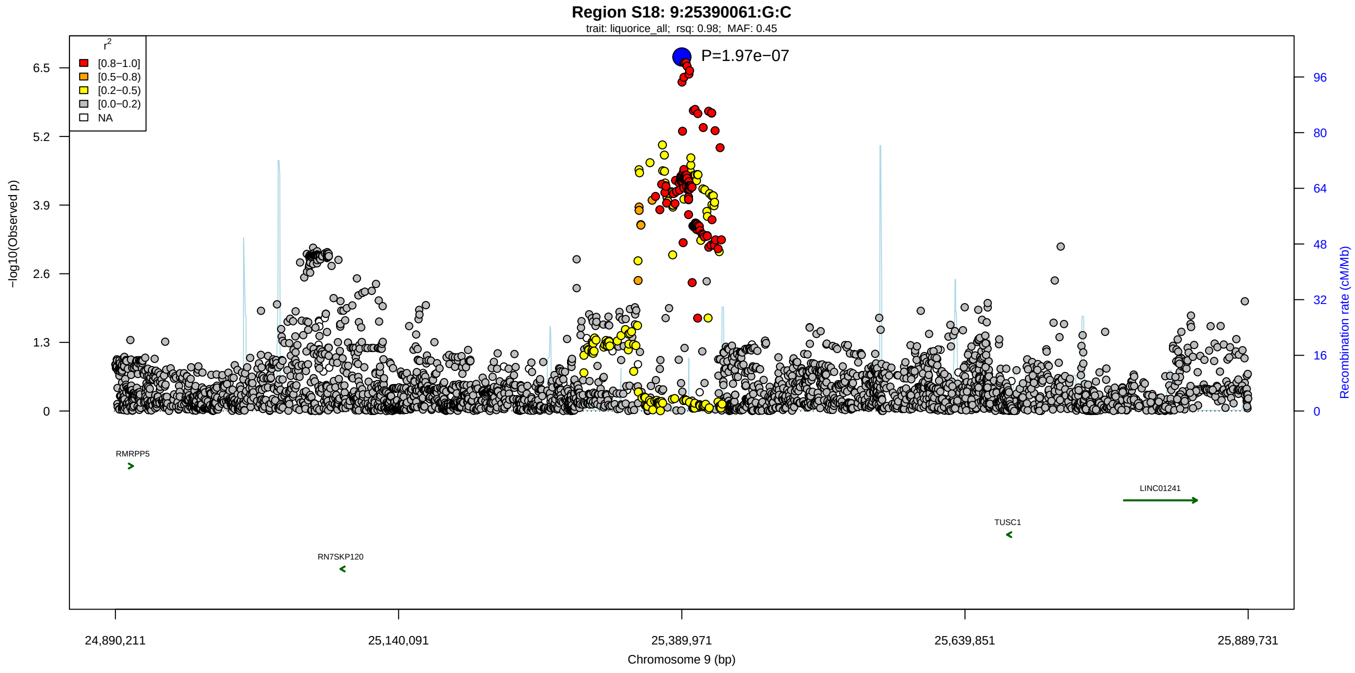
Region S18: 9:25390061:G:C
trait: liquorice_all; rsq: 0.98; MAF: 0.45
r2
[0.8−1.0]
[0.5−0.8)
[0.2−0.5)
[0.0−0.2)
NA
P=1.97e−07
−log10(Observed p)
Recombination rate (cM/Mb)
RMRPP5
LINC01241
TUSC1
RN7SKP120
24,890,211
25,140,091
25,389,971
25,639,851
25,889,731
Chromosome 9 (bp)

Region S19: 9:99769266:G:A

trait: banana_female; rsq: 0.9; MAF: 0.02

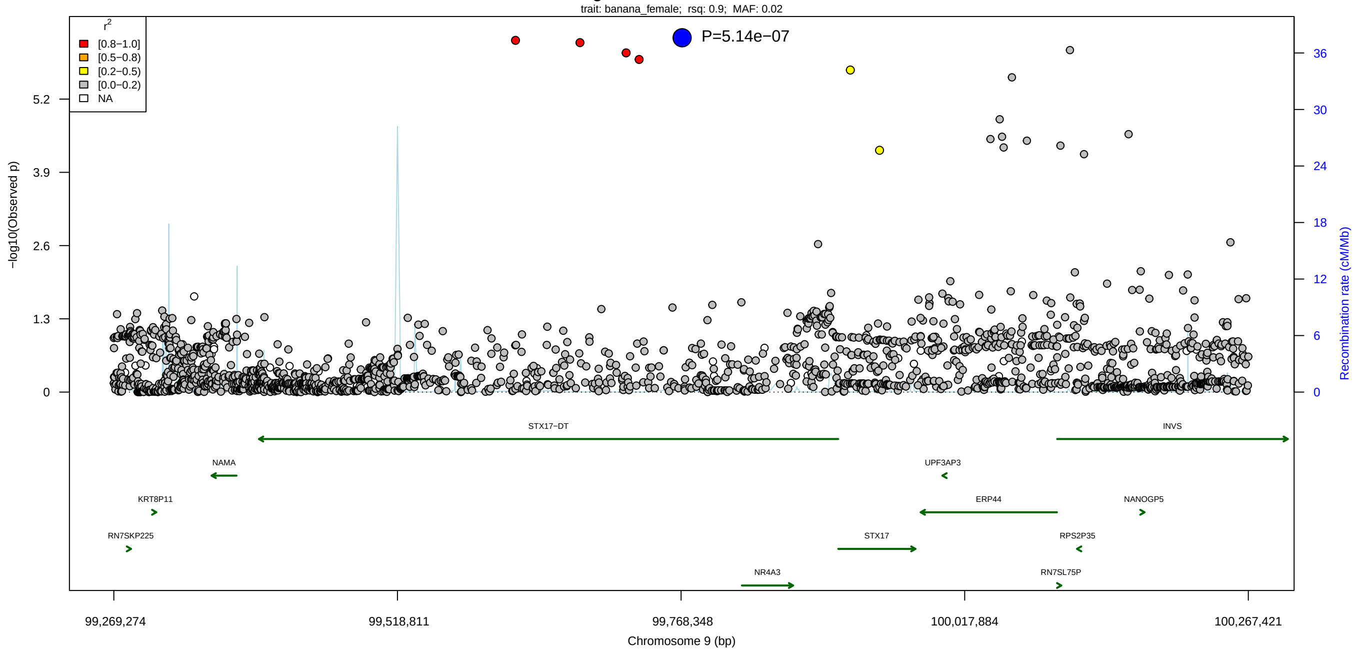

Region S20: 10:27299438:C:T

trait: cinnamon_all; rsq: 0.96; MAF: 0.02

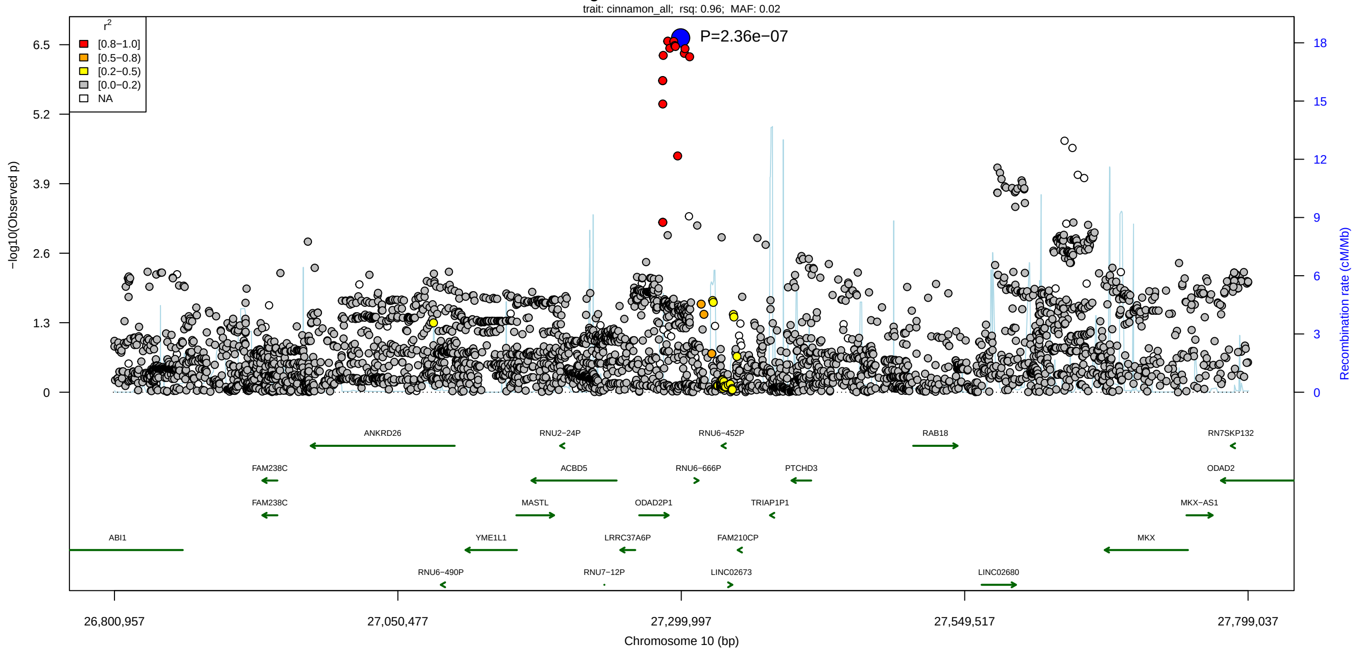

Region S21: 10:43551296:G:A

trait: leather_male; rsq: 0.85; MAF: 0.02

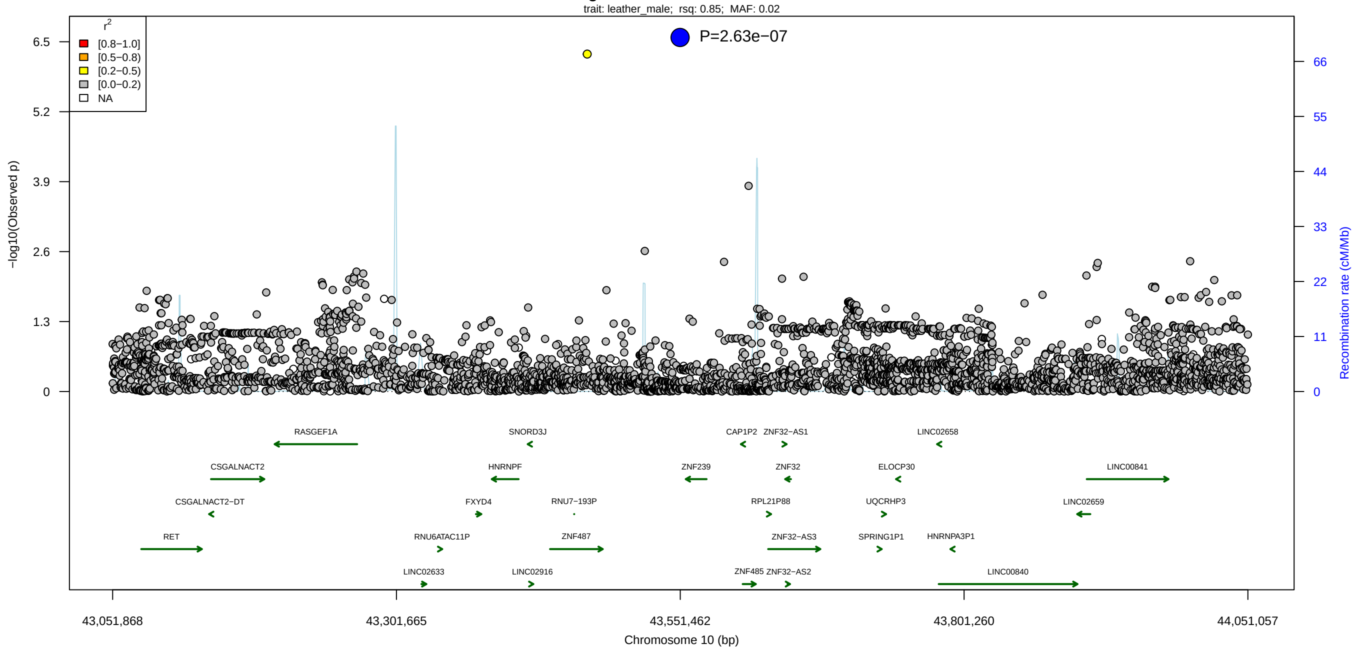

Region S22: 10:106493647:A:G

trait: cinnamon_all; rsq: 0.97; MAF: 0.48

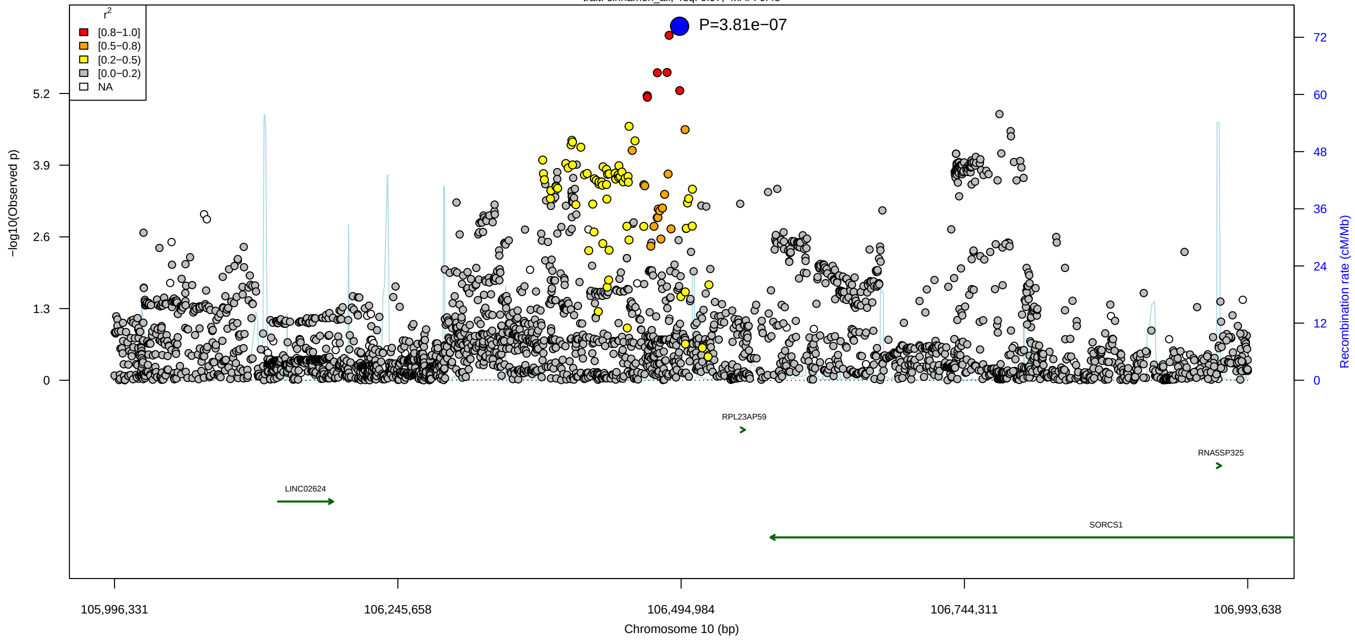

Region S23: 11:69873331:C:T

trait: cinnamon_male; rsq: 0.92; MAF: 0.31

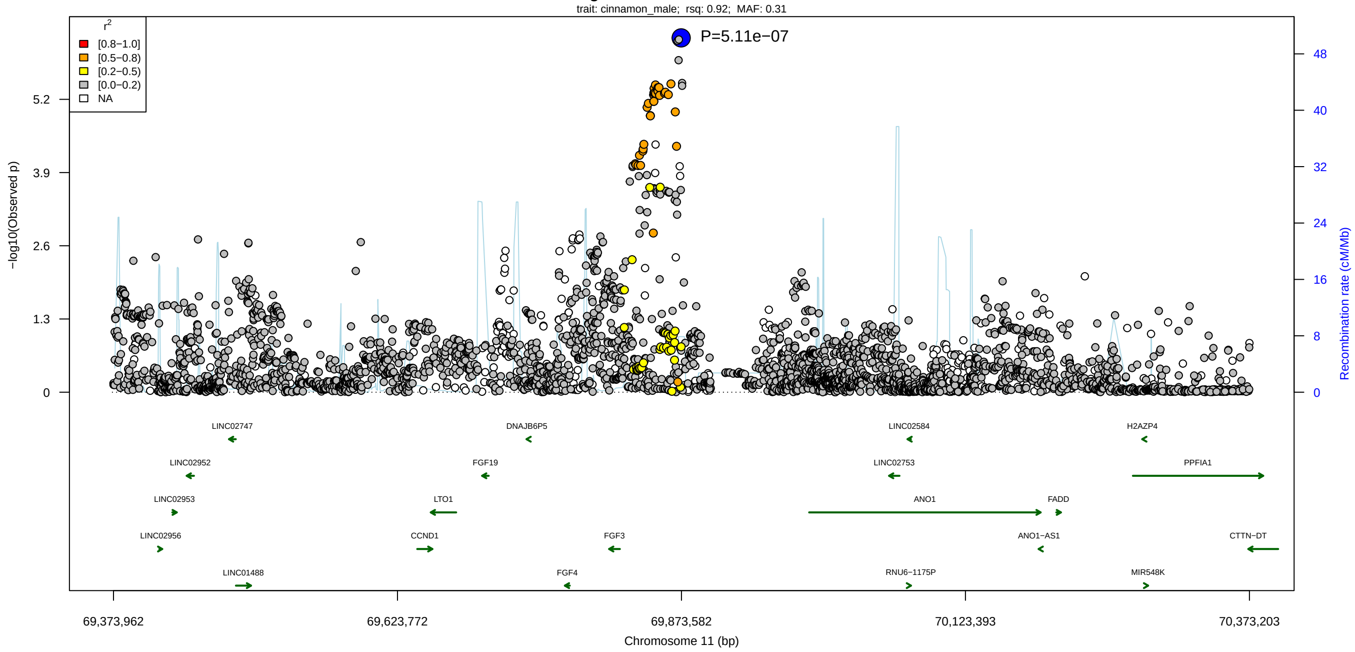

Region S24: 11:80068171:AT:A

trait: leather_male; rsq: 0.99; MAF: 0.17

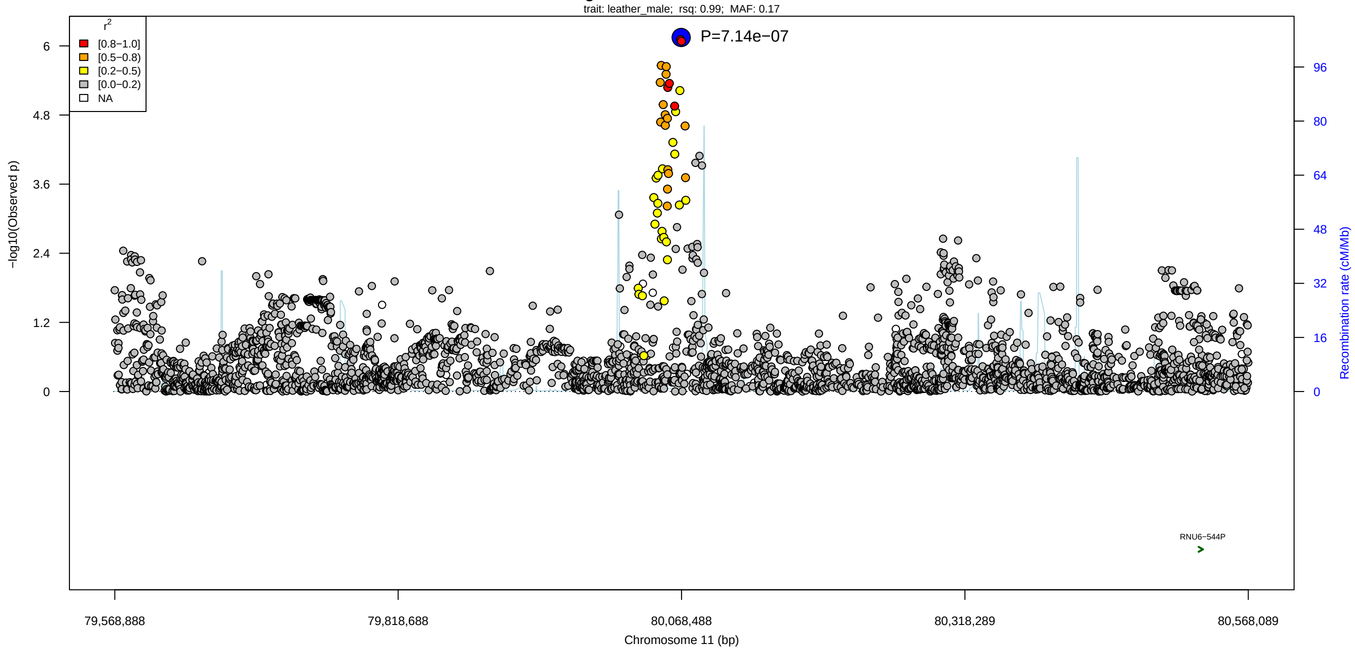

Region S25: 11:109527399:T:C

trait: liquorice_female; rsq: 0.88; MAF: 0.08

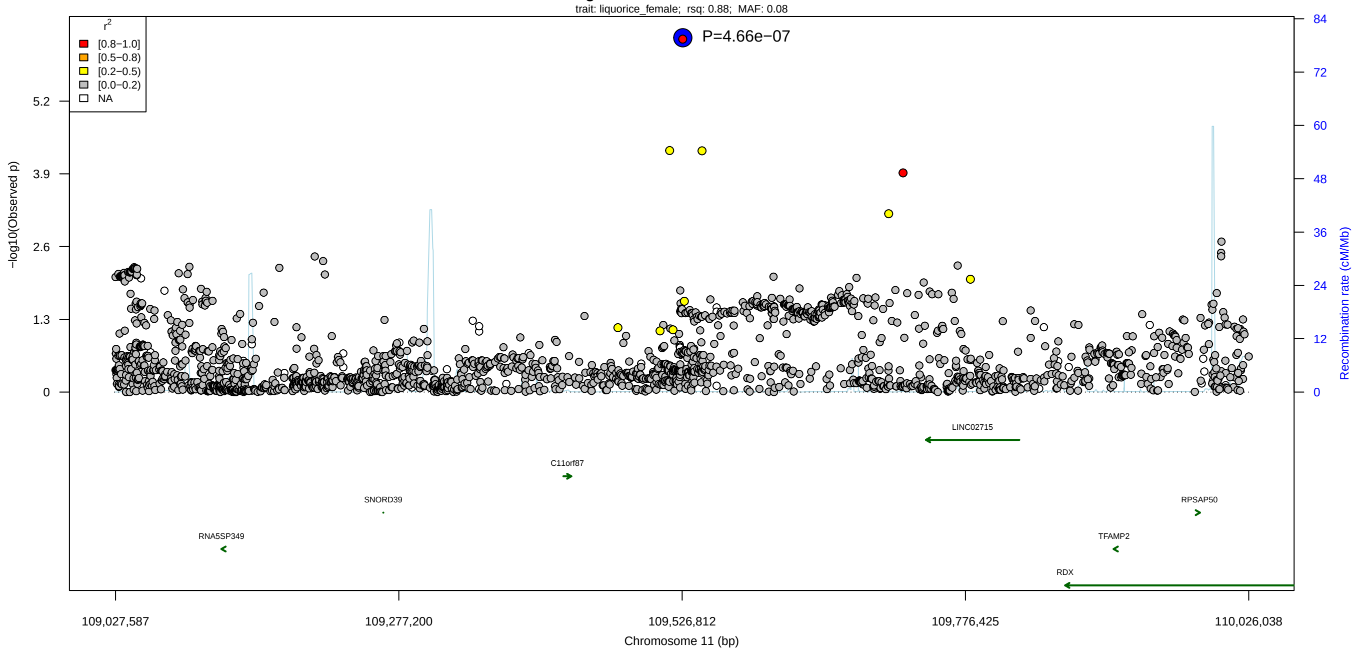

Region S26: 12:55330672:C:T

trait: liquorice_all; rsq: 0.98; MAF: 0.19

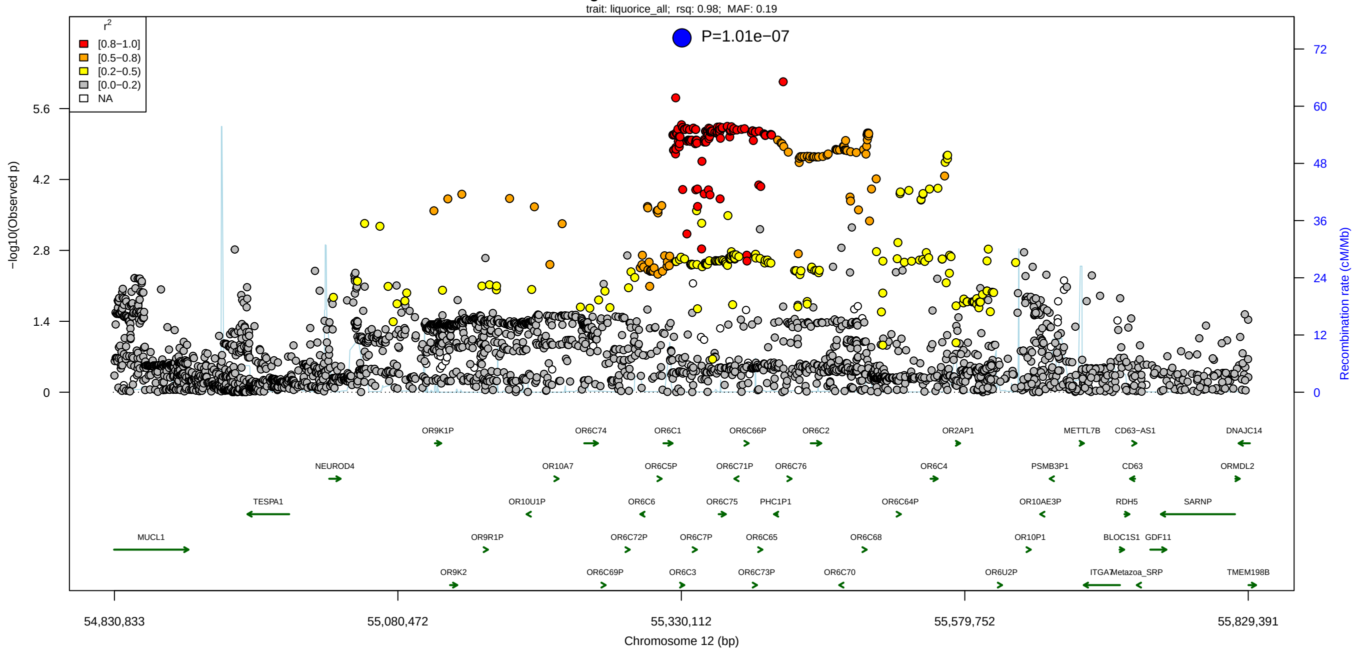

Region S27: 13:25769605:G:C

trait: orange_female; rsq: 0.95; MAF: 0.03

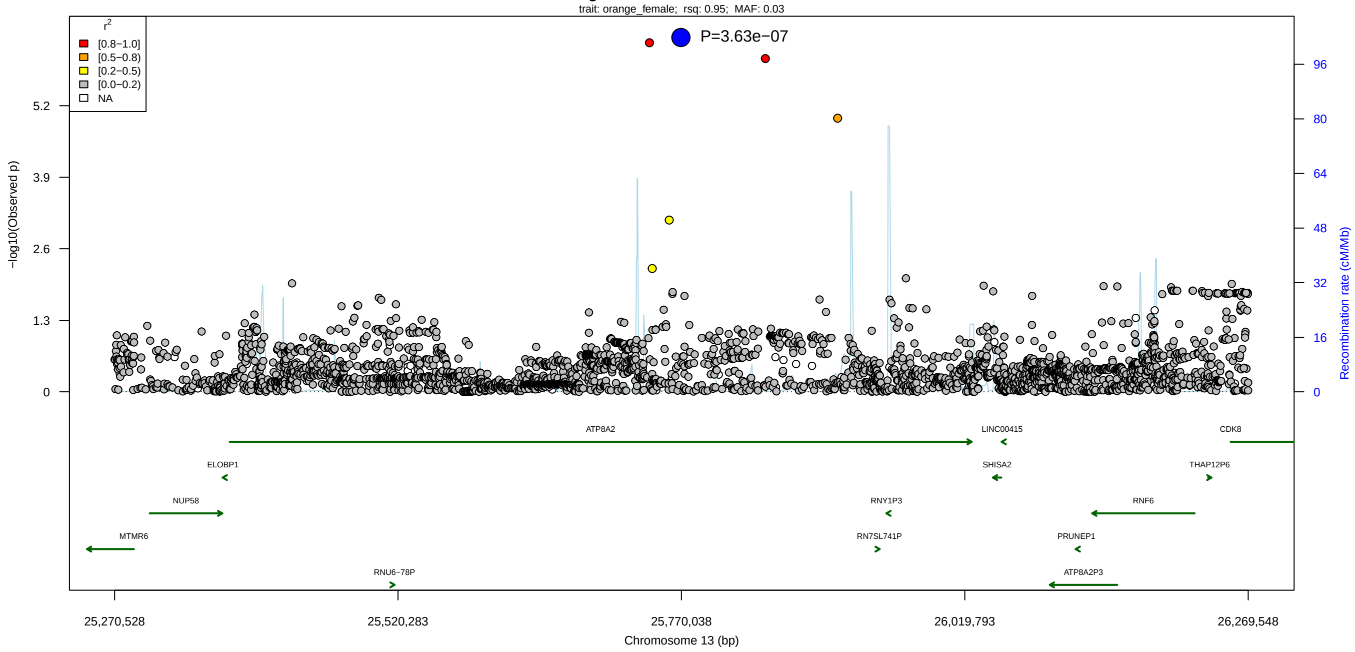

Region S28: 13:63734703:A:T

trait: pineapple_all; rsq: 0.97; MAF: 0.04

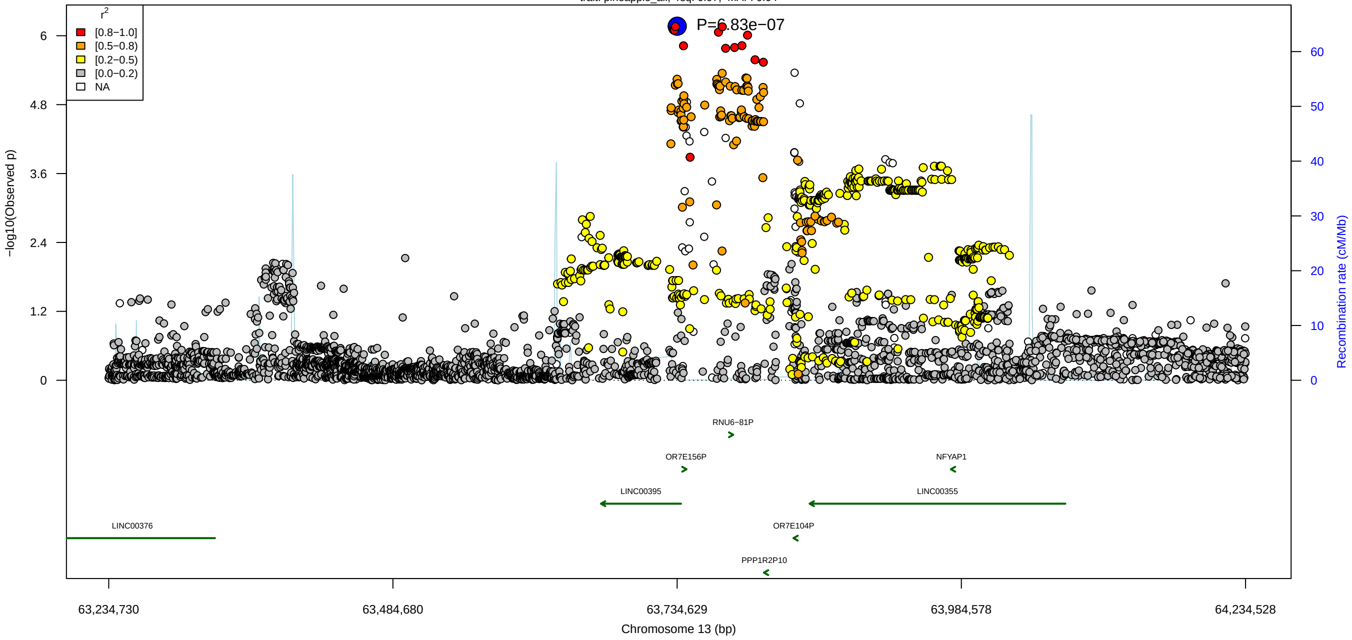

Region S29: 14:39079650:G:C

trait: liquorice_male; rsq: 0.92; MAF: 0.01

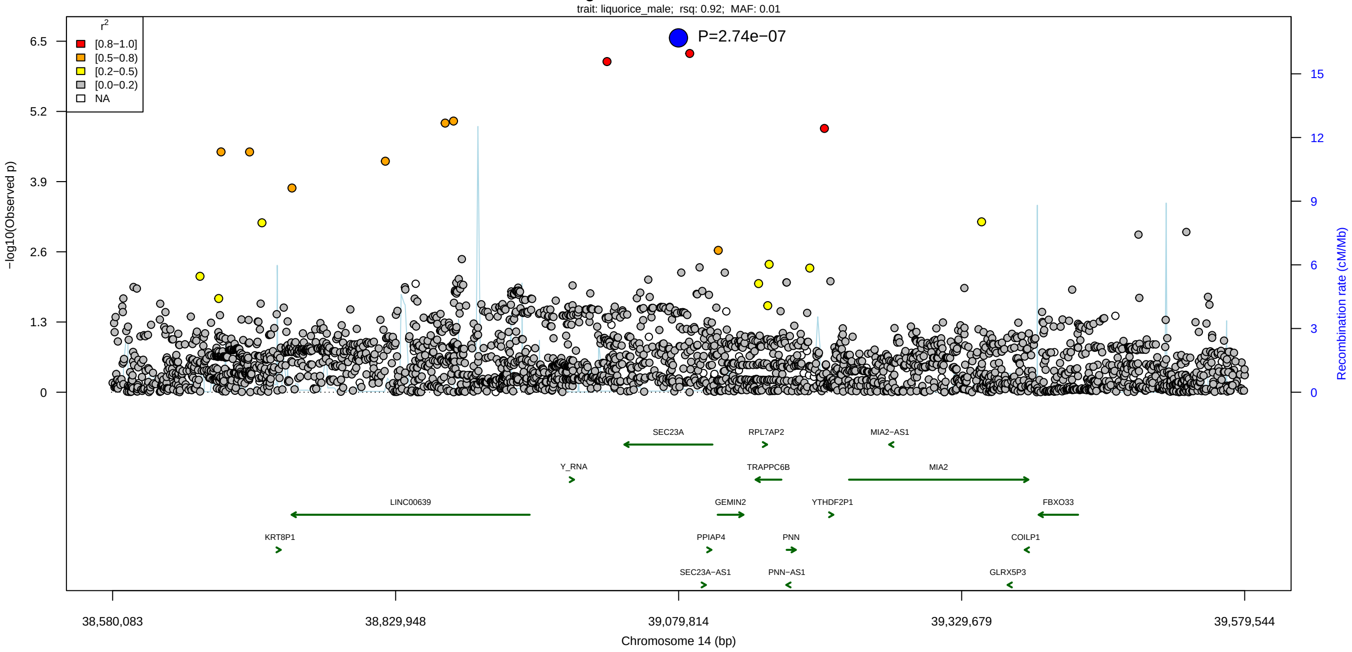

Region S30: 14:63068624:AT:A

trait: cinnamon_all; rsq: 0.99; MAF: 0.26

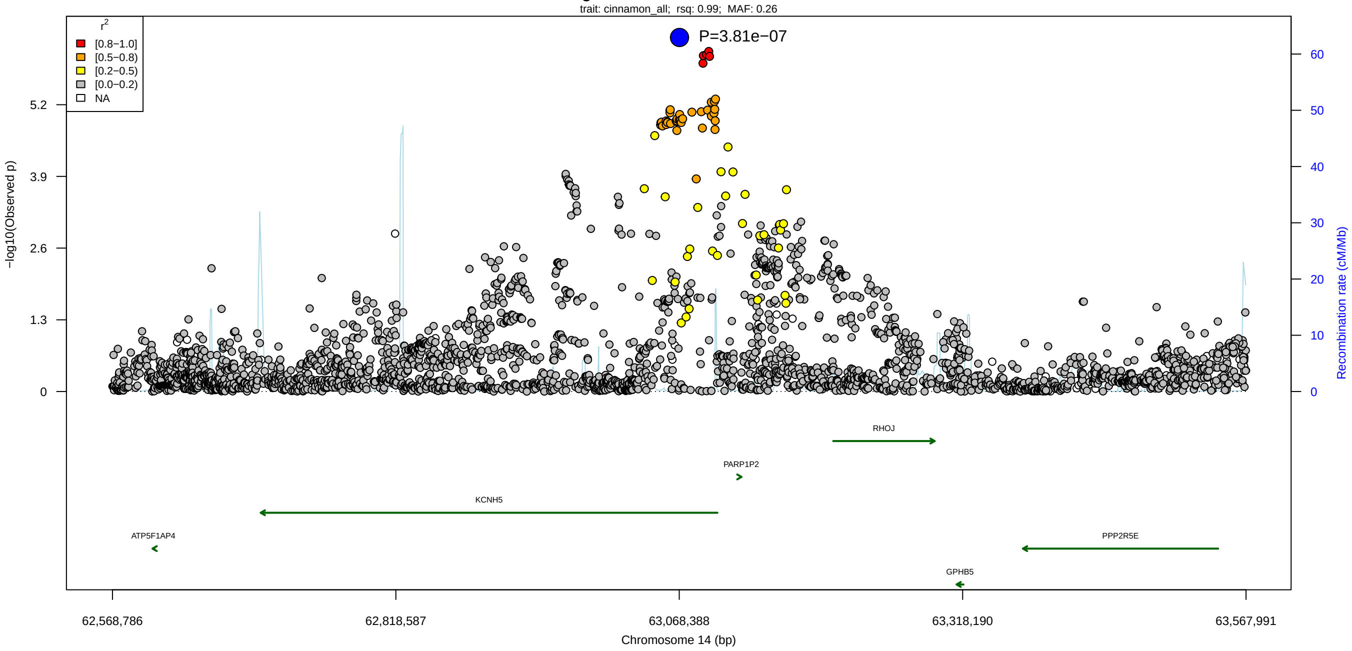

Region S31: 14:73587517:G:A

trait: fish_all; rsq: 0.97; MAF: 0.29

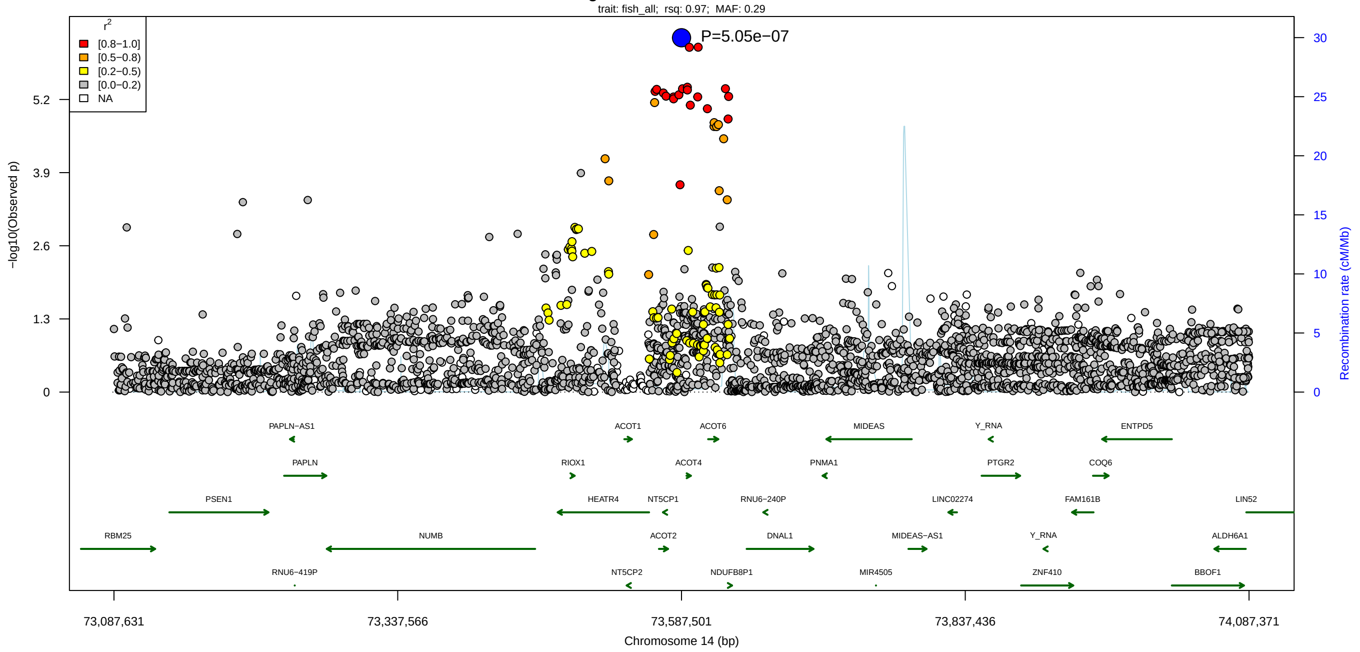

Region S32: 18:12279273:C:G

trait: pineapple_all; rsq: 0.94; MAF: 0.04

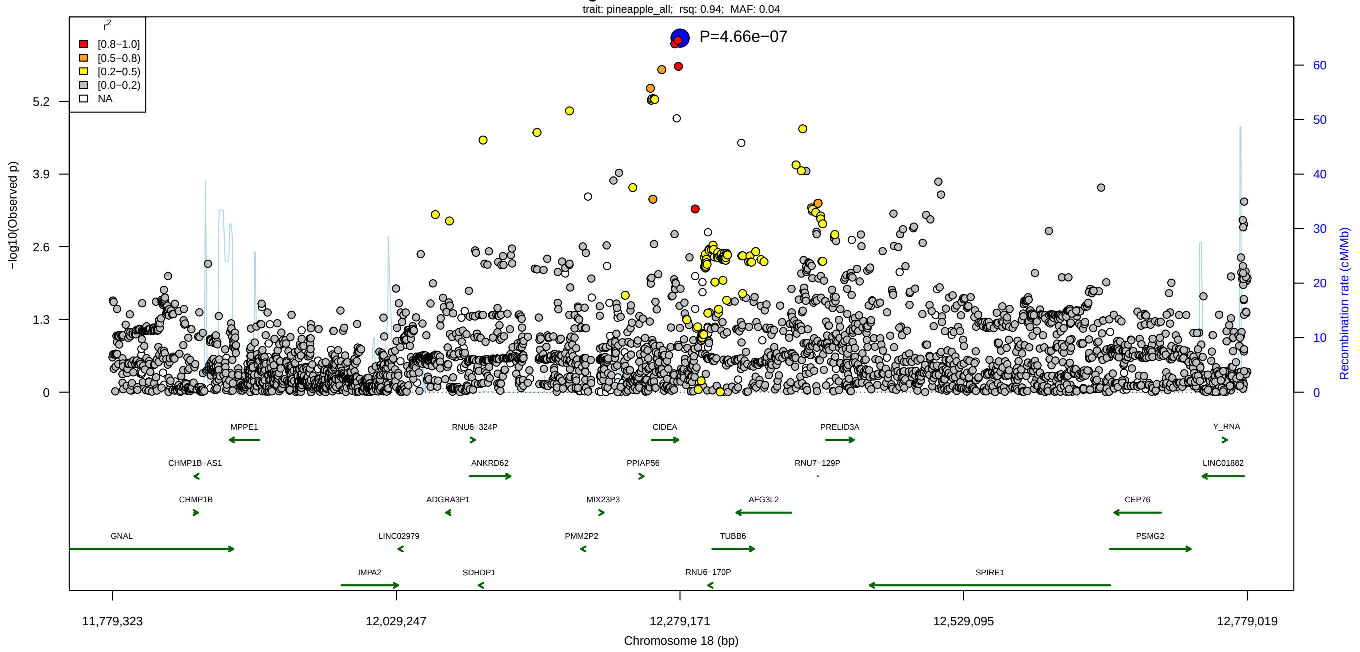

Region S33: 19:17280108:G:C

trait: SCORE_female; rsq: 0.89; MAF: 0.48

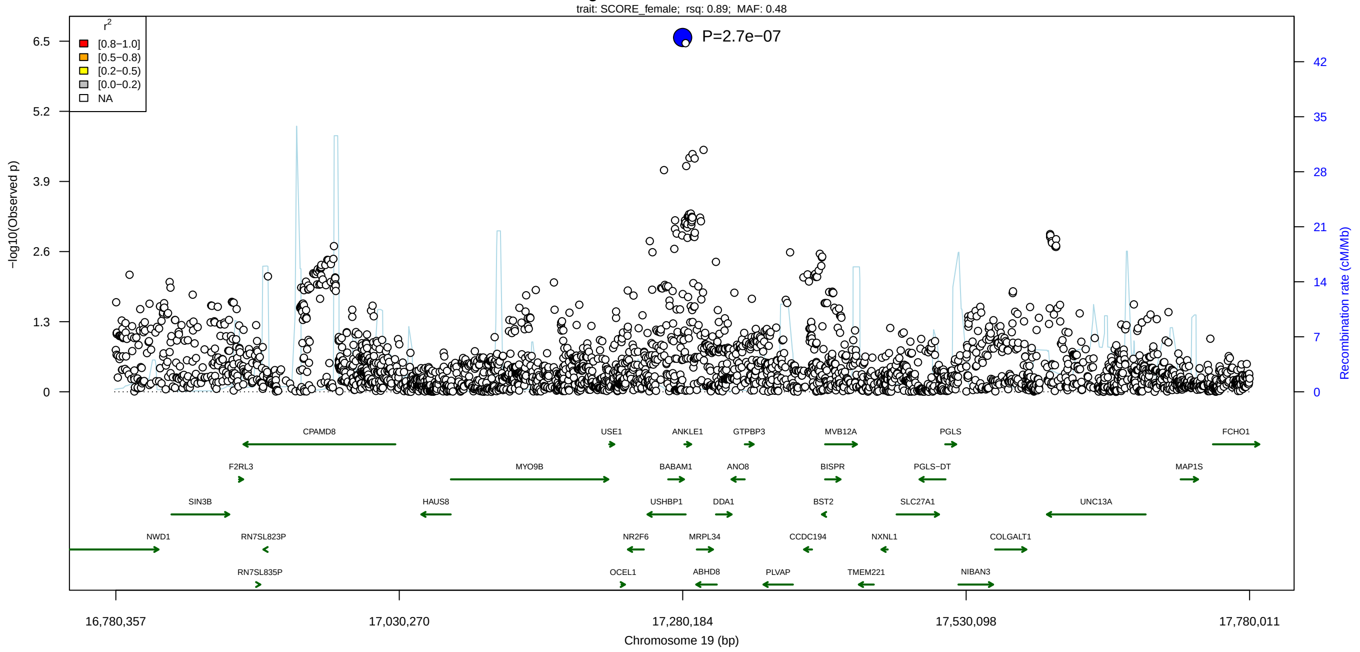

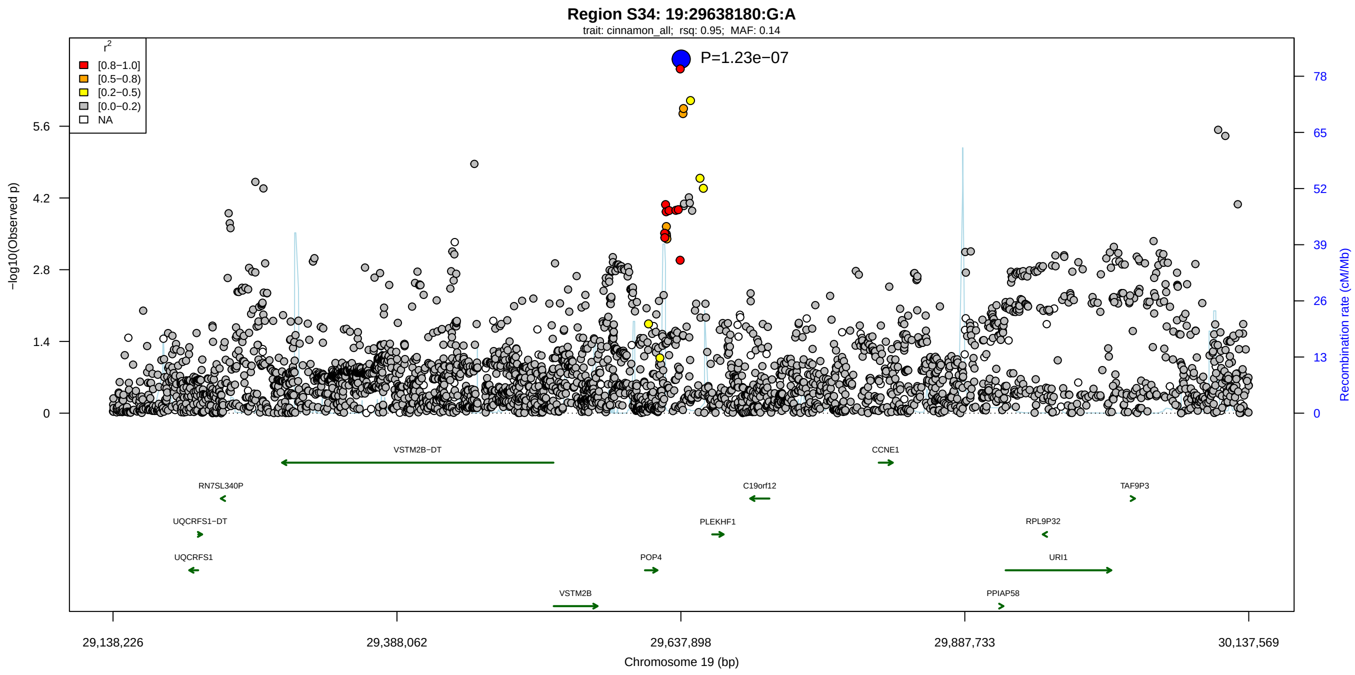

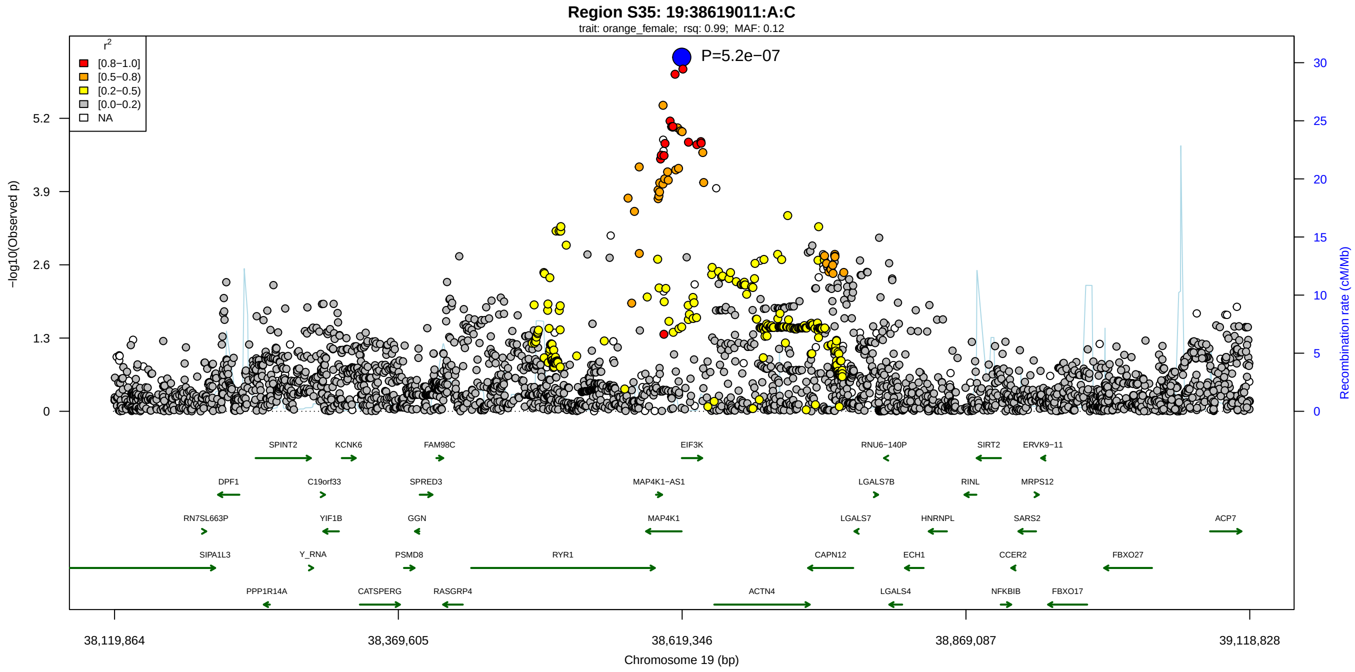

Supplementary Figure 7: Regional association plots of identified loci with suggestive significance. Data is shown for the best associated phenotype for each locus. The two-sided p-value of the association is shown. Strength of LD with the index variant is represented by colour.

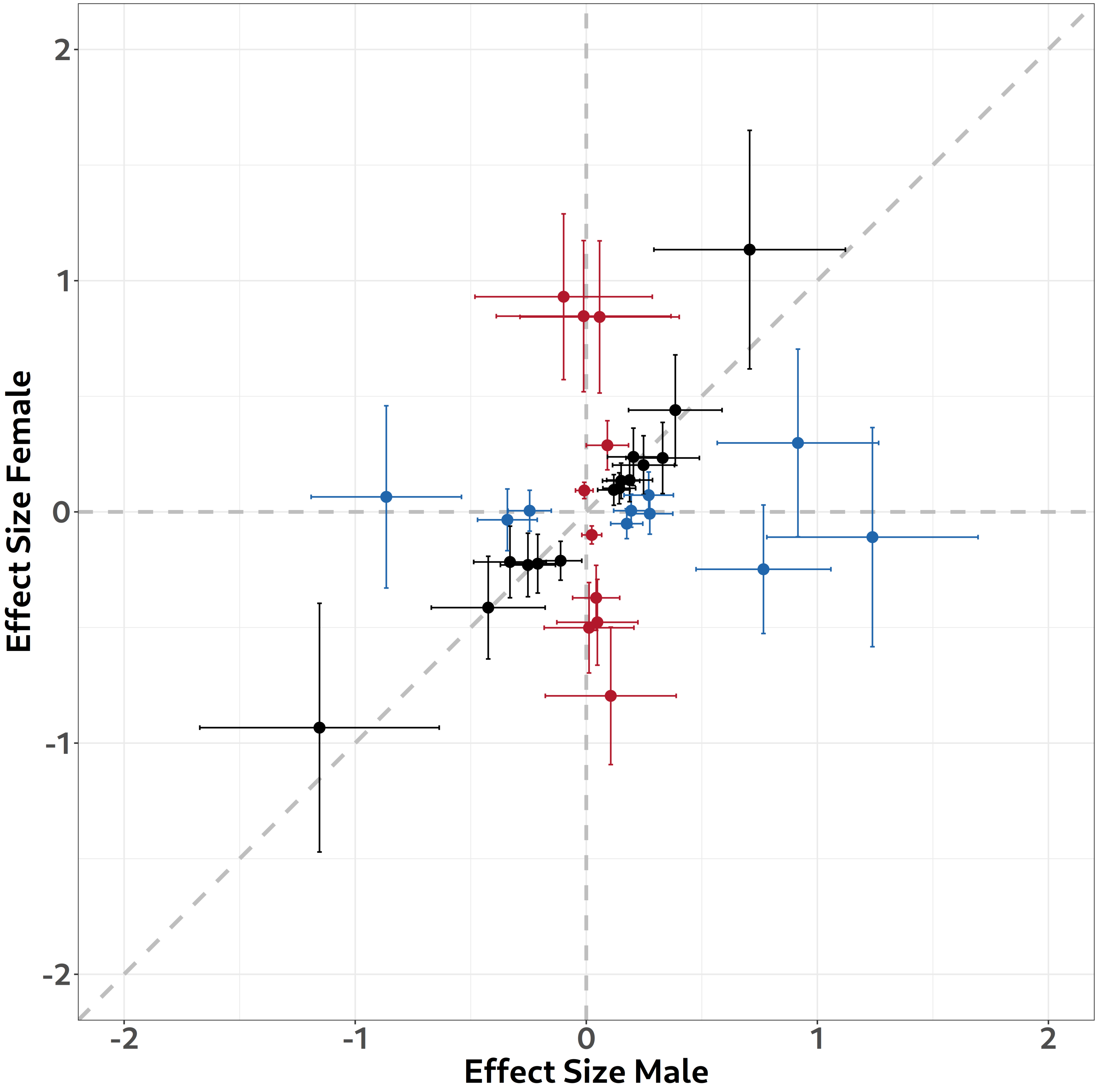

Supplementary Figure 8: SNP-by-sex interaction analysis for index variants of suggestive loci. Comparison of effect sizes (β coefficient of additive genetic effects) and respective 95% confidence limits between males (x-axis) and females (y-axis) are displayed. 20 variants achieved significance (red = higher effect size in females, blue = higher effect size in males).

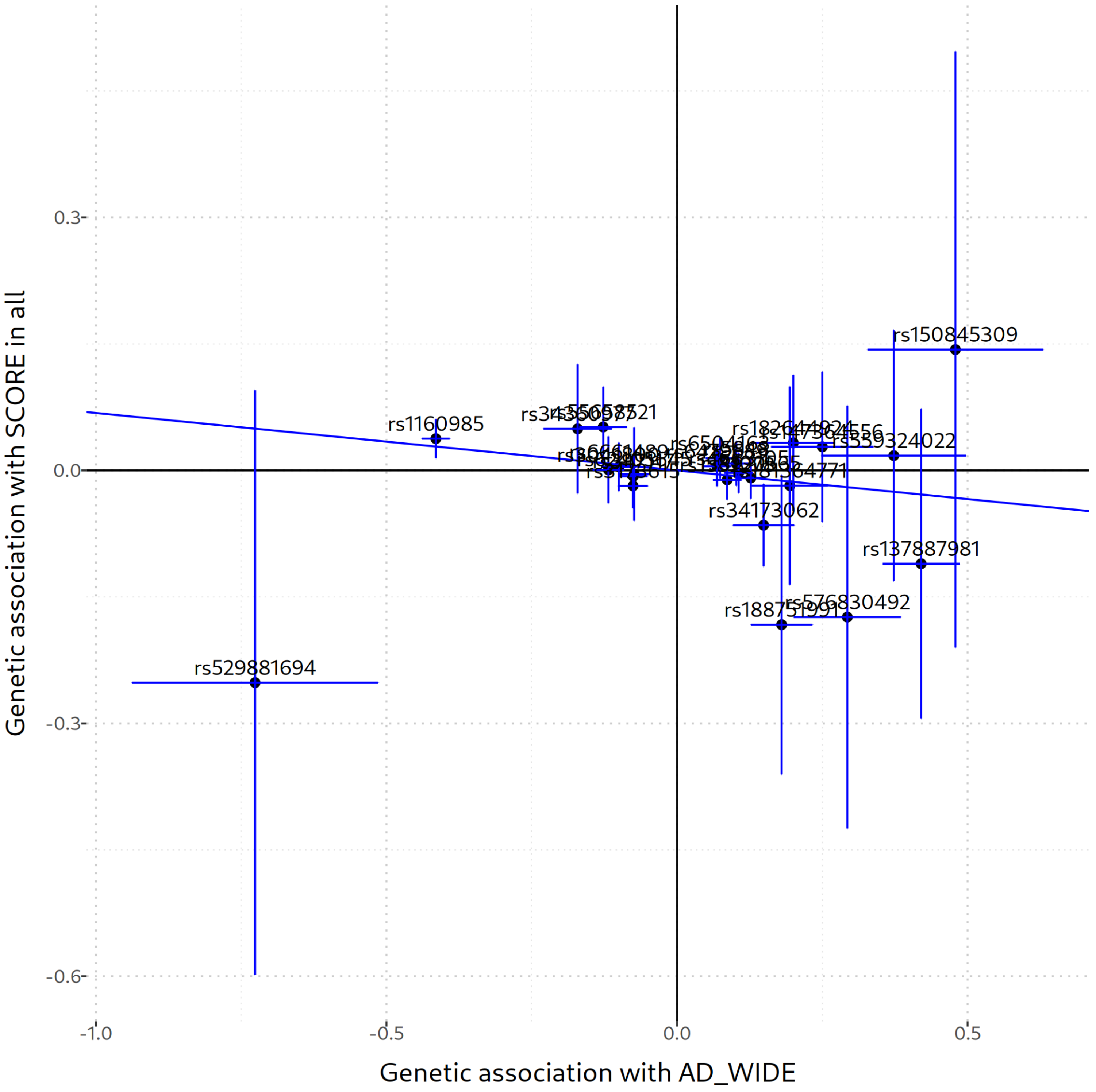

Supplementary Figure 9: Beta-beta plot of SNP association with Alzheimer's disease (wide definition) and score in the overall analysis. For each SNP the rsID is given. The bars indicate the 95% confidence intervals for the exposure and outcome. The blue line indicates the IVW estimate (β_{IVW} = -0.07, p-value (adj.) = 0.013).

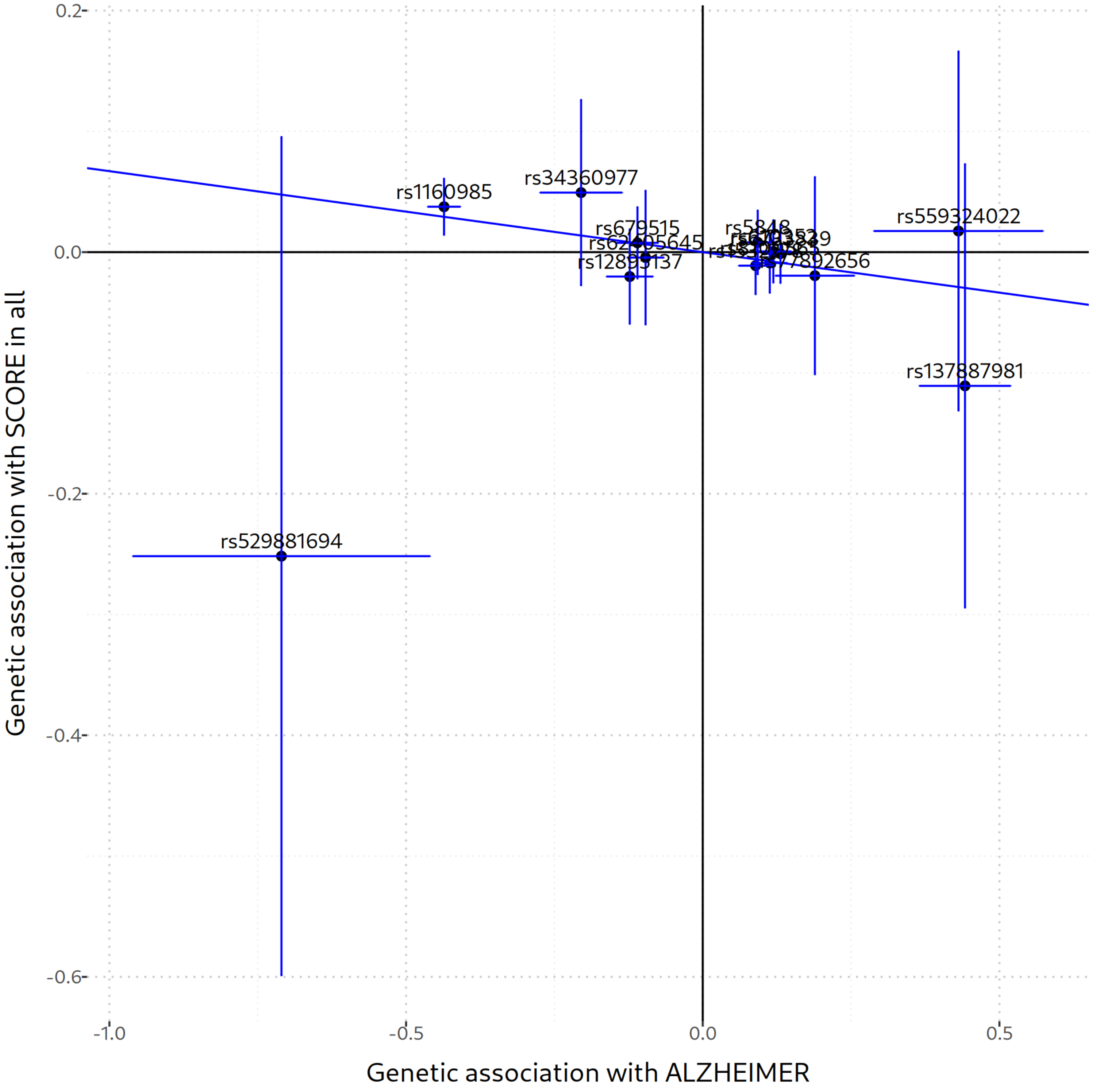

Supplementary Figure 10: Beta-beta plot of SNP association with Alzheimer's disease (standard definition) and score in the overall analysis. For each SNP the rsID is given. The bars indicate the 95% confidence intervals for the exposure and outcome. The blue line indicates the IVW estimate (β_{IVW} = -0.07, p-value (adj.) = 0.009).

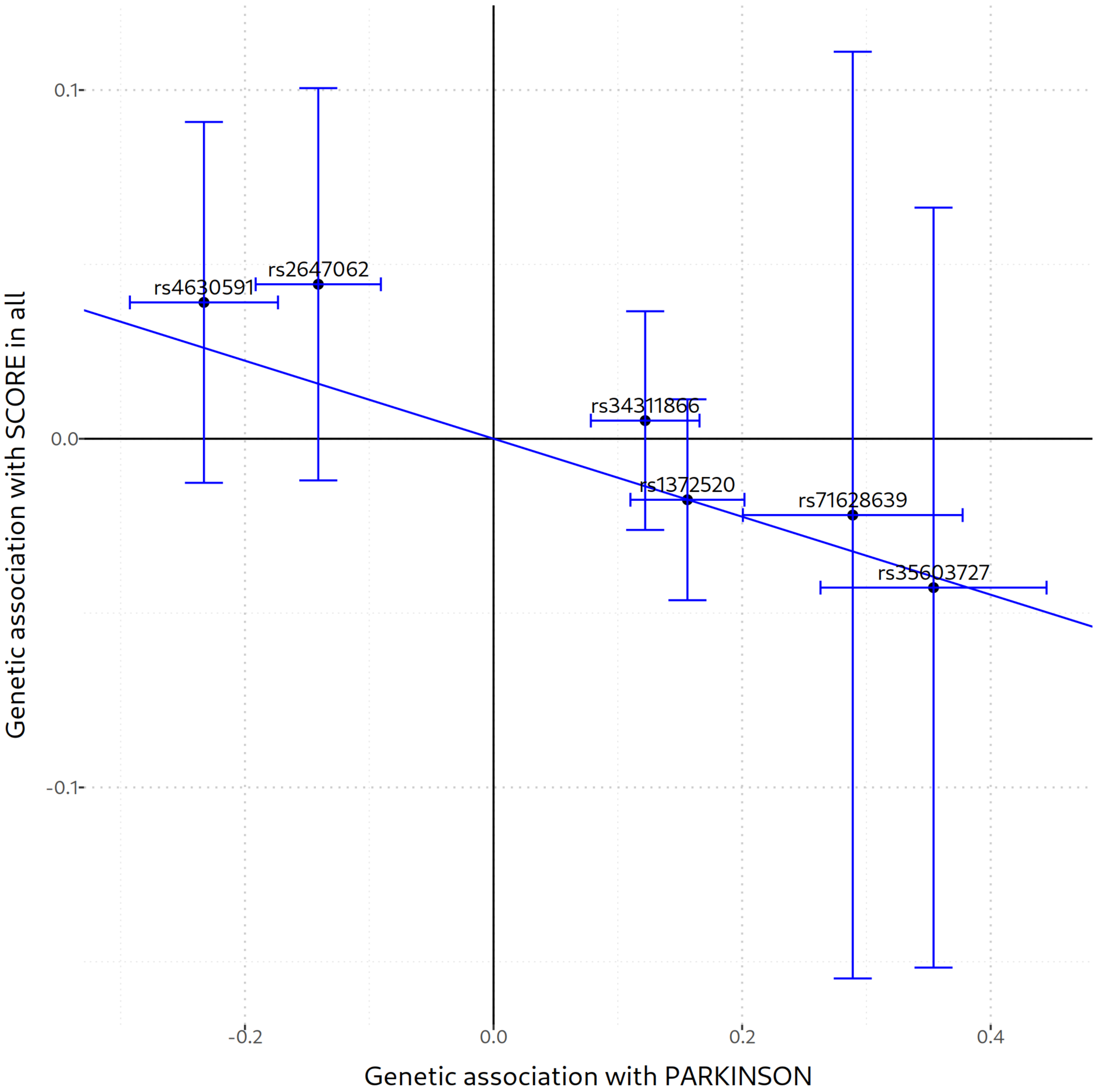

Supplementary Figure 11: Beta-beta plot of SNP association with Parkinson's disease and score in the overall analysis. For each SNP the rsID is given. The bars indicate the 95% confidence intervals for the exposure and outcome. The blue line indicates the IVW estimate (β_{IVW} = -0.11, p-value (adj.) = 0.042).

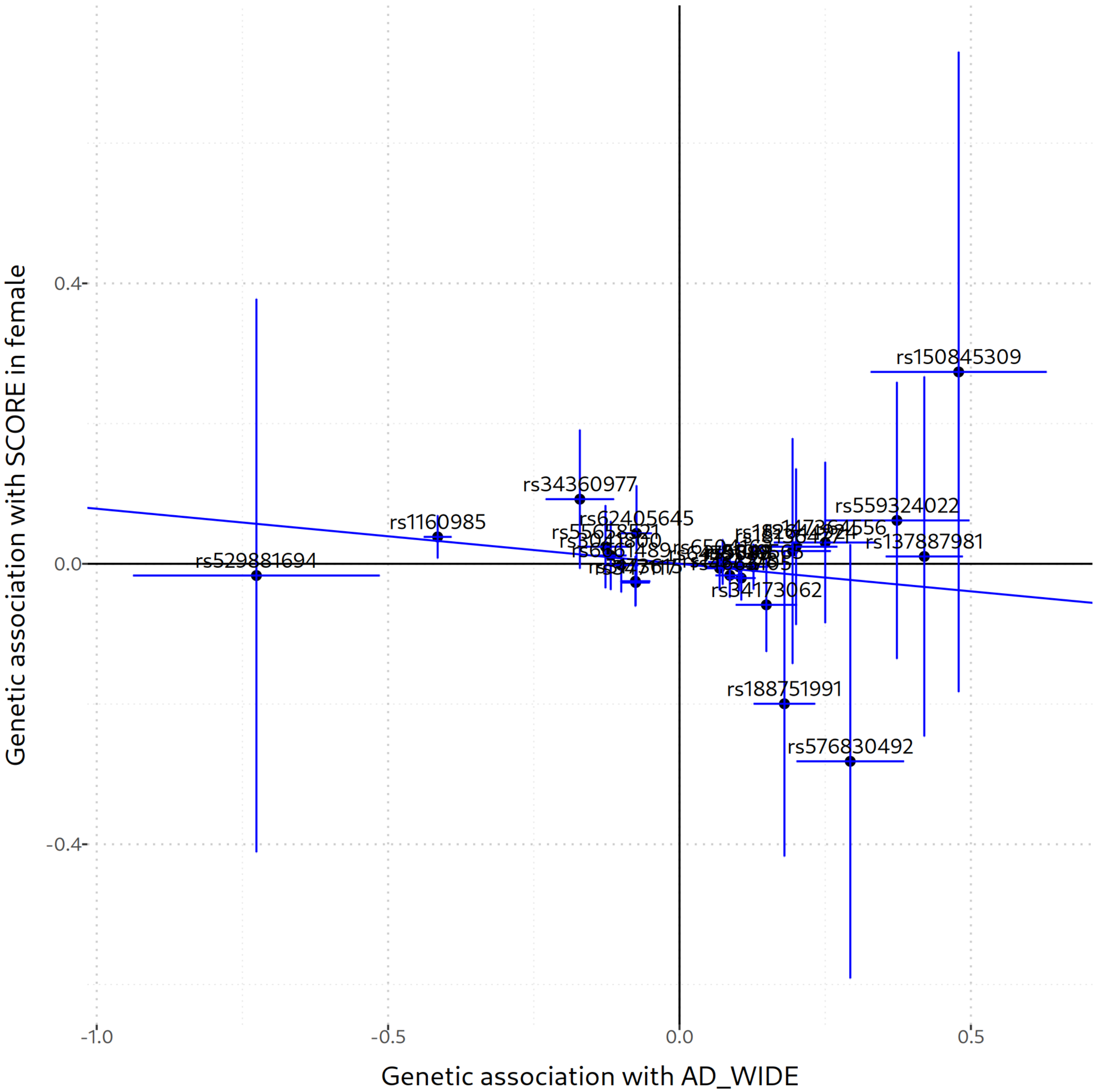

Supplementary Figure 12: Beta-beta plot of SNP association with Alzheimer's disease (wide definition) and score in the analysis of females. For each SNP the rsID is given. The bars indicate the 95% confidence intervals for the exposure and outcome. The blue line indicates the IVW estimate (β_{IVW} = -0.08, p-value (adj.) = 0.014).

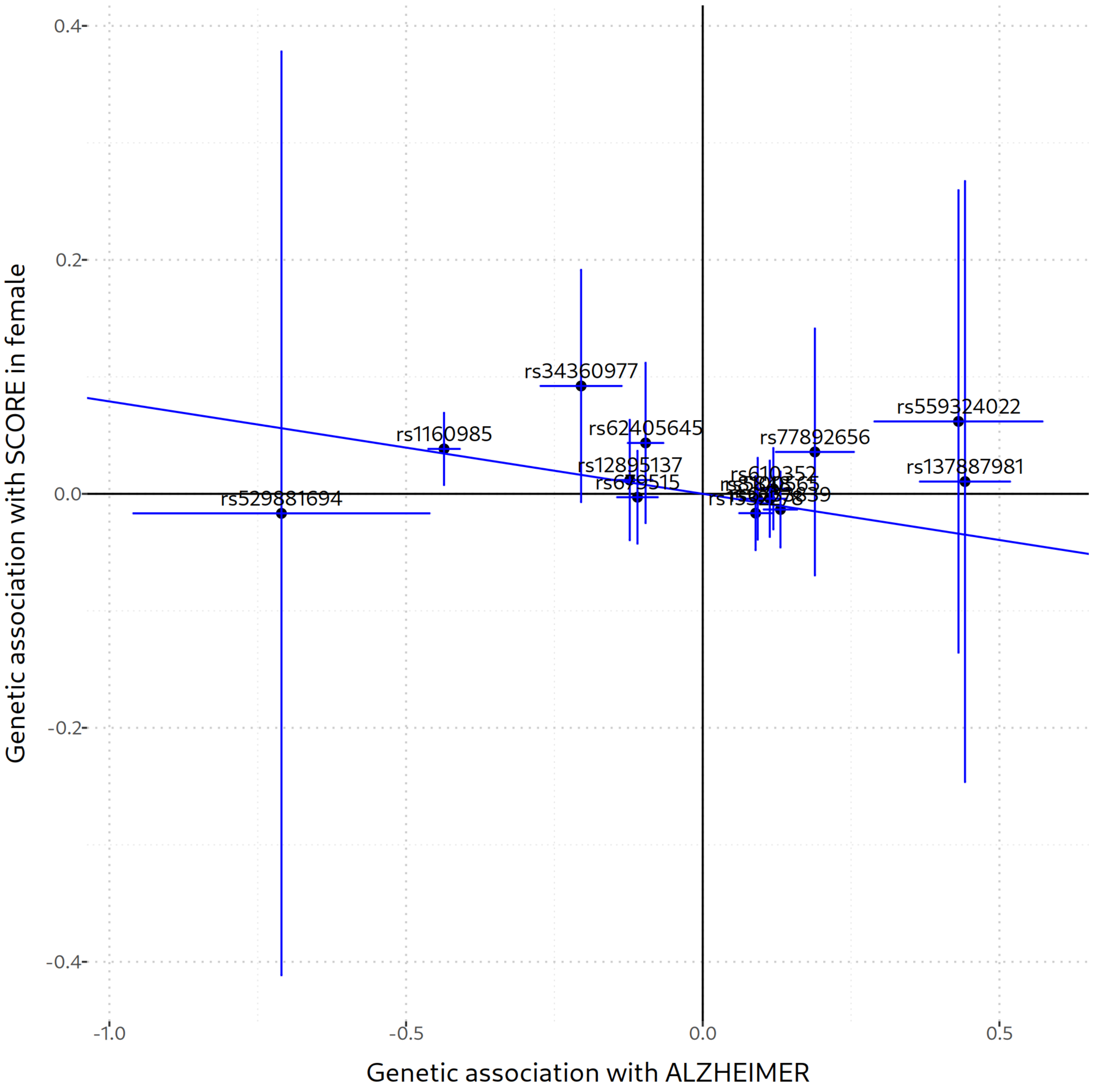

Supplementary Figure 13: Beta-beta plot of SNP association with Alzheimer's disease (standard definition) and score in the analysis of females. For each SNP the rsID is given. The bars indicate the 95% confidence intervals for the exposure and outcome. The blue line indicates the IVW estimate (β_{IVW} = -0.08, p-value (adj.) = 0.014).

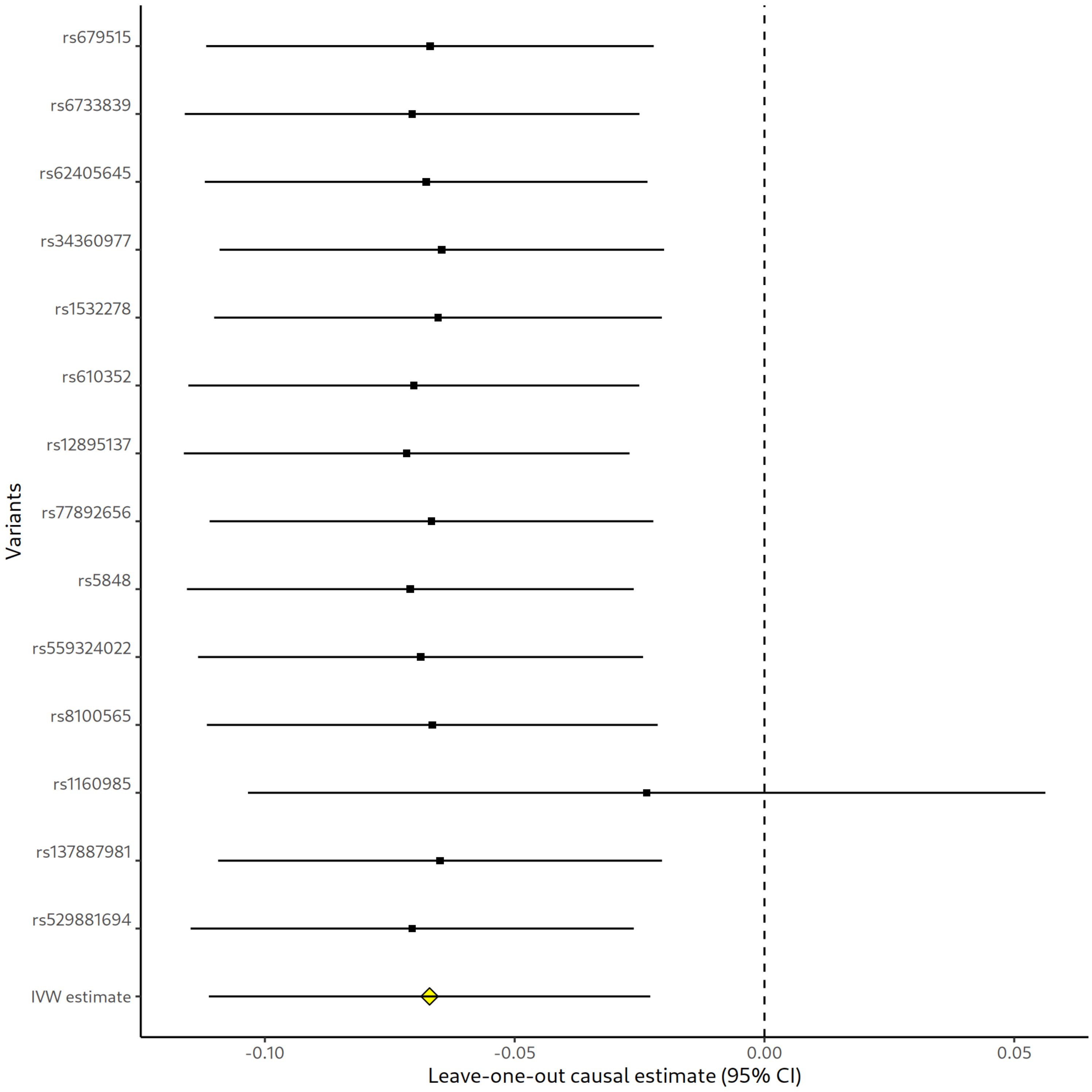

Supplementary Figure 14: Leave-one-out analysis of the causal effect of Alzheimer's disease (standard definition) on the identification score in the overall analysis. The SNP indicated at the y-axis is the one that is left out. Leaving out the SNP rs1160985 would result in a non-significant IVW estimate, i.e. the causal effect is driven by this variant.

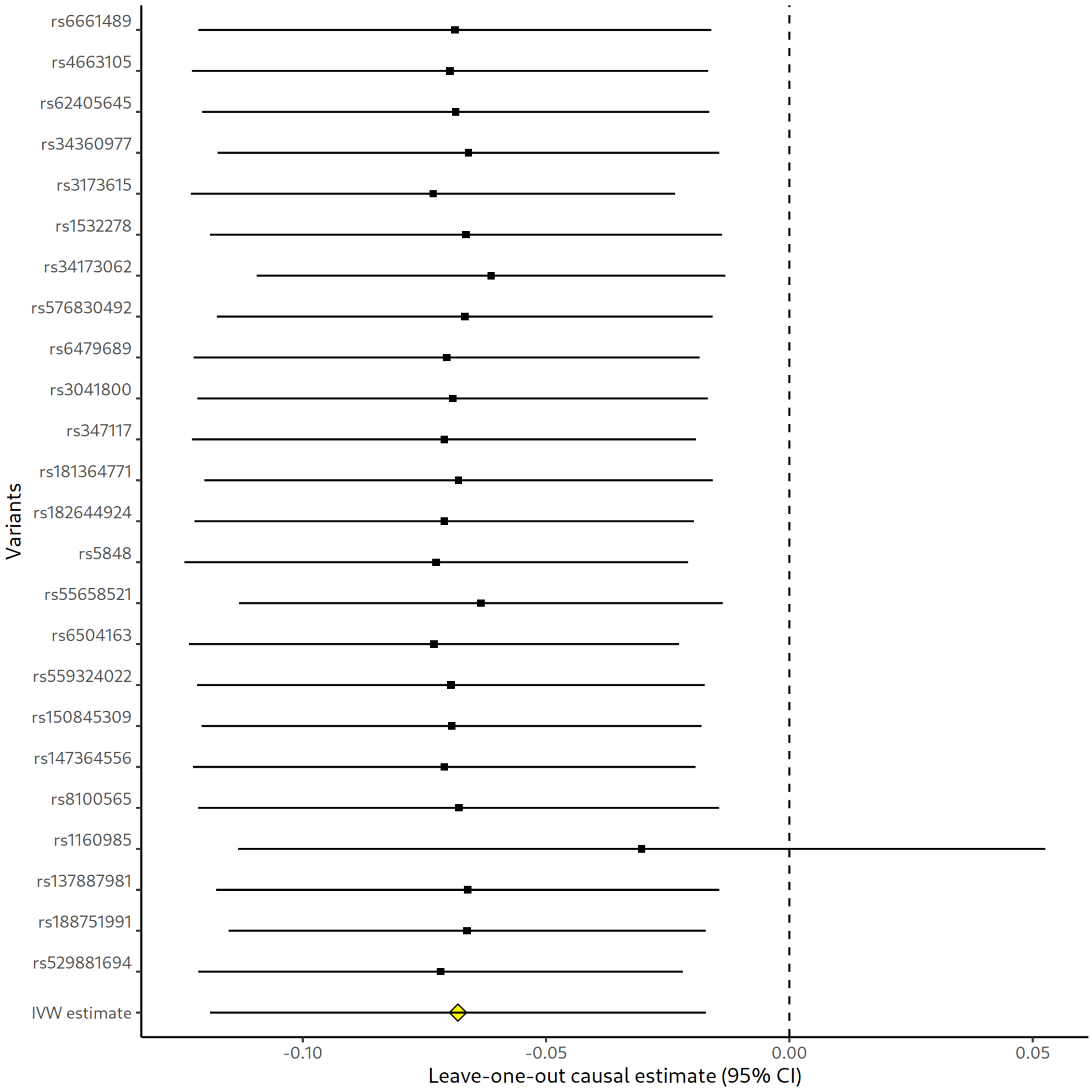

Supplementary Figure 15: Leave-one-out analysis of the causal effect of Alzheimer's disease (wide definition) on the identification score in the overall analysis. The SNP indicated at the y-axis is the one that is left out. Leaving out the SNP rs1160985 would result in a non-significant IVW estimate, i.e. the causal effect is driven by this variant.

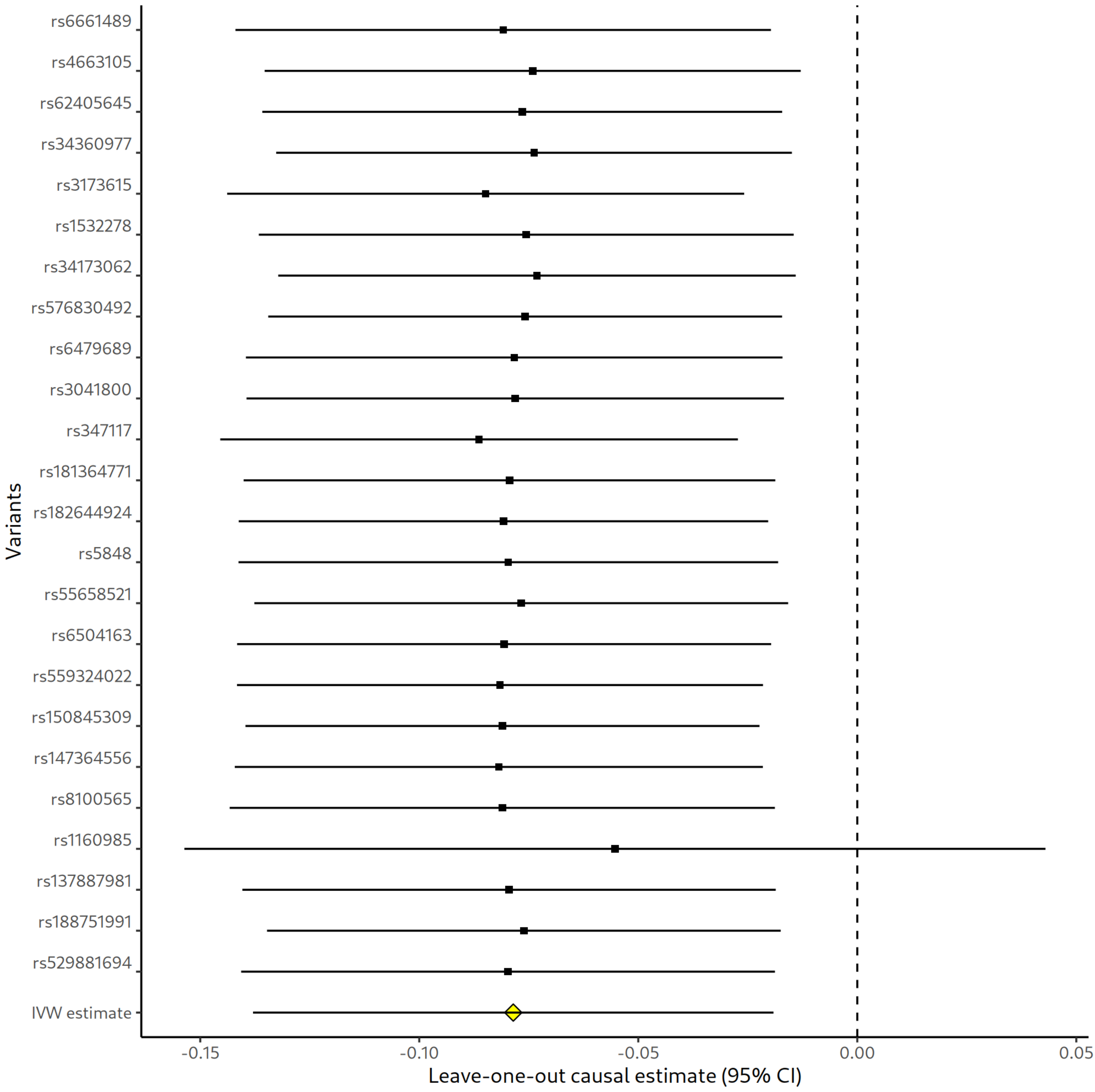

Supplementary Figure 16: Leave-one-out analysis of the causal effect of Alzheimer's disease (wide definition) on the identification score in females. The SNP indicated at the y-axis is the one that is left out. Leaving out the SNP rs1160985 would result in a non-significant IVW estimate, i.e. the causal effect is driven by this variant.

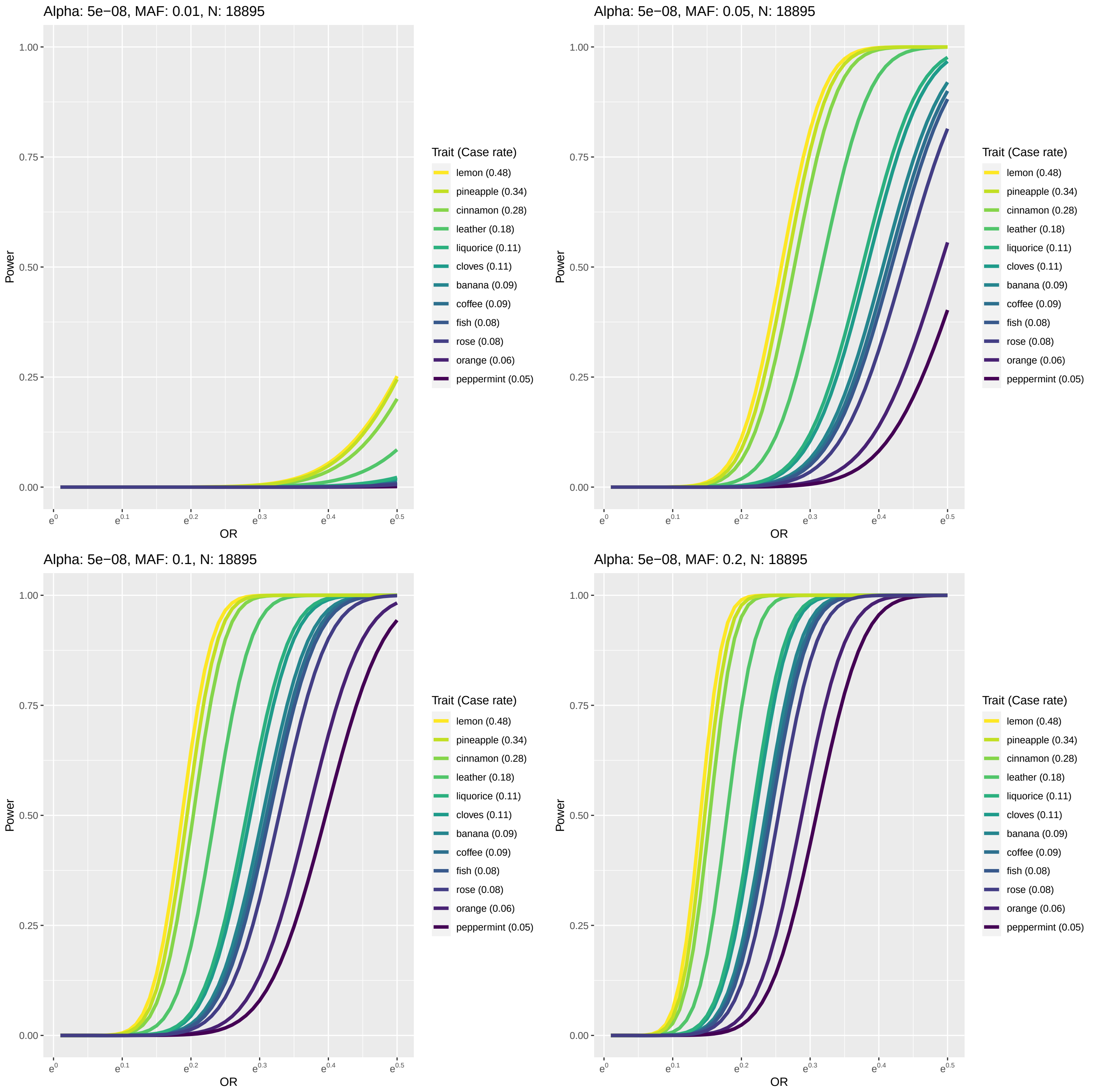

Supplementary Figure 17: Power analysis of individual odour detection rates. Power was calculated for different minor allele frequencies (MAF) at different odds ratios (OR) assuming an additive genetic model and genome-wide significance threshold. Case rates were determined for each stick based on misidentification frequency across all participating studies. Traits are ordered with decreasing case rate.

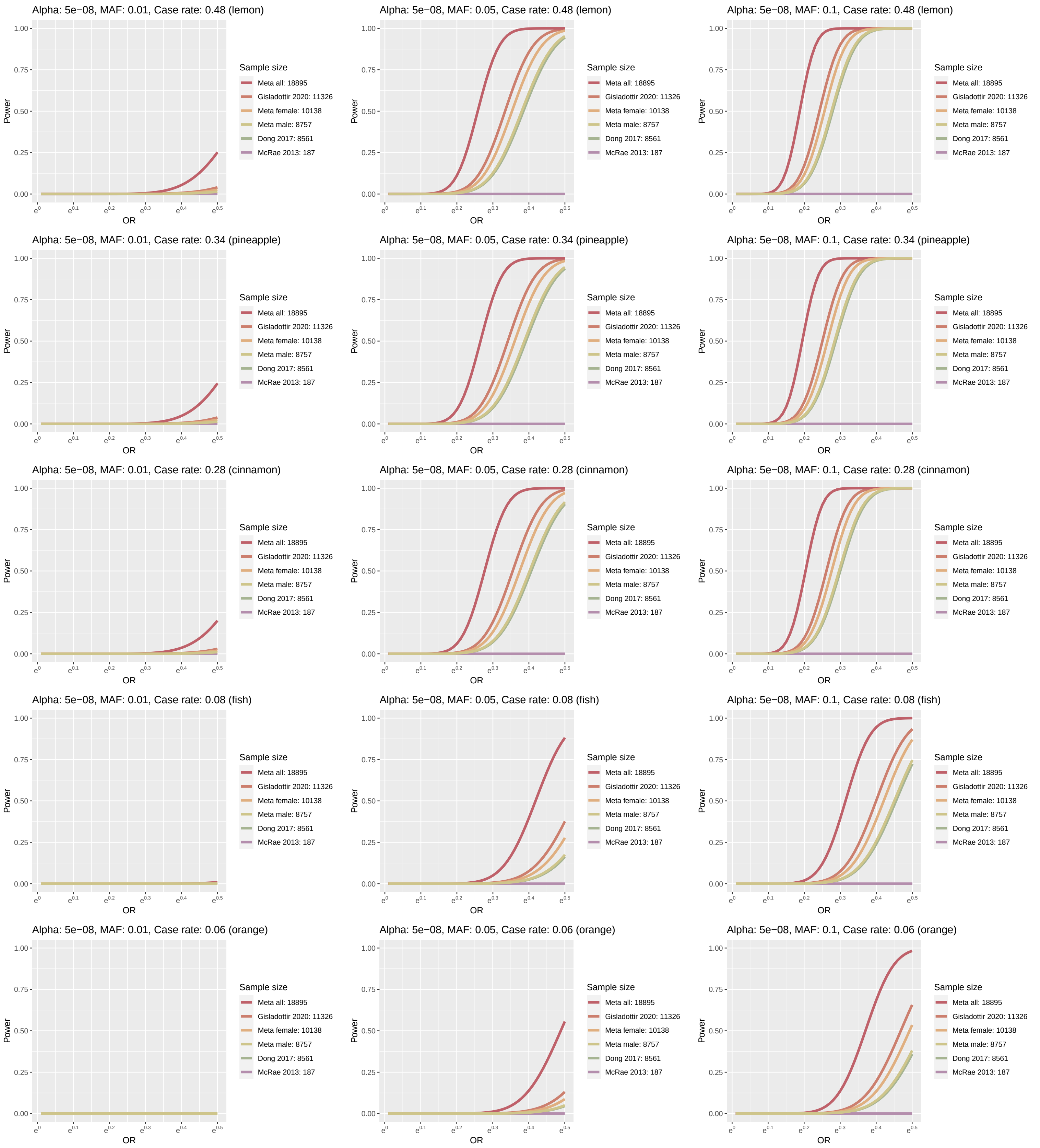

Supplementary Figure 18: Power analysis for different sample sizes. Power was calculated for different minor allele frequencies (MAF) at different odds ratios (OR) assuming an additive genetic model and genome-wide significance threshold. Case rates were determined across all participating studies for odours with genome-wide significant variants. Maximal possible sample size was assumed for each study. The entry 'Meta' refers to the sample size of the present study.

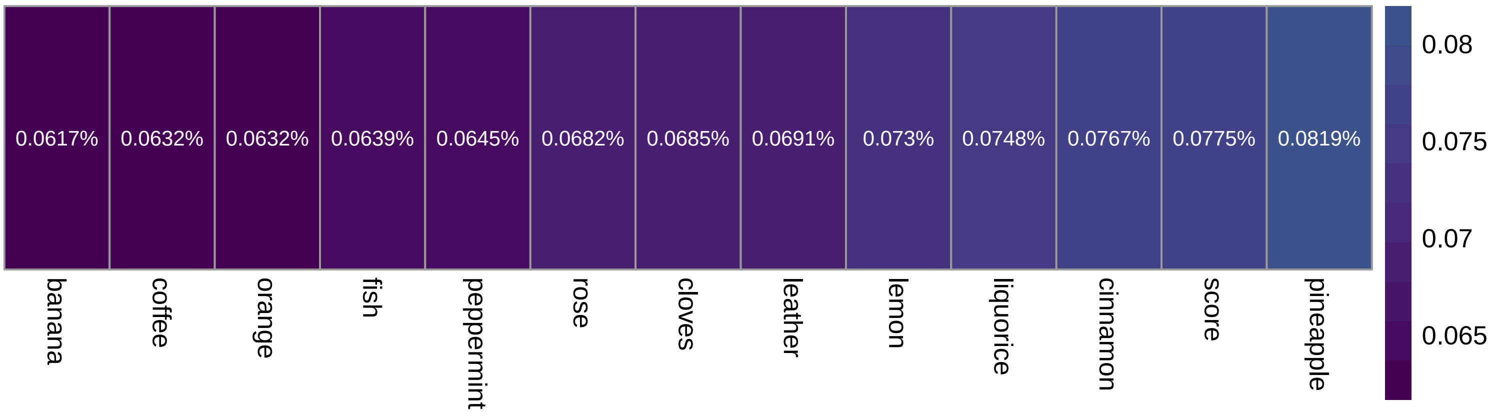

Supplementary Figure 19: Percentage of quality-controlled variants with high heterogeneity. The fraction of variants with $I^2 \geq 85\%$ is shown in percent for the overall analysis of each trait (x-axis). Quality control was applied without filtering for heterogeneity. Traits are sorted from left to right by increasing fraction of heterogeneous variants.

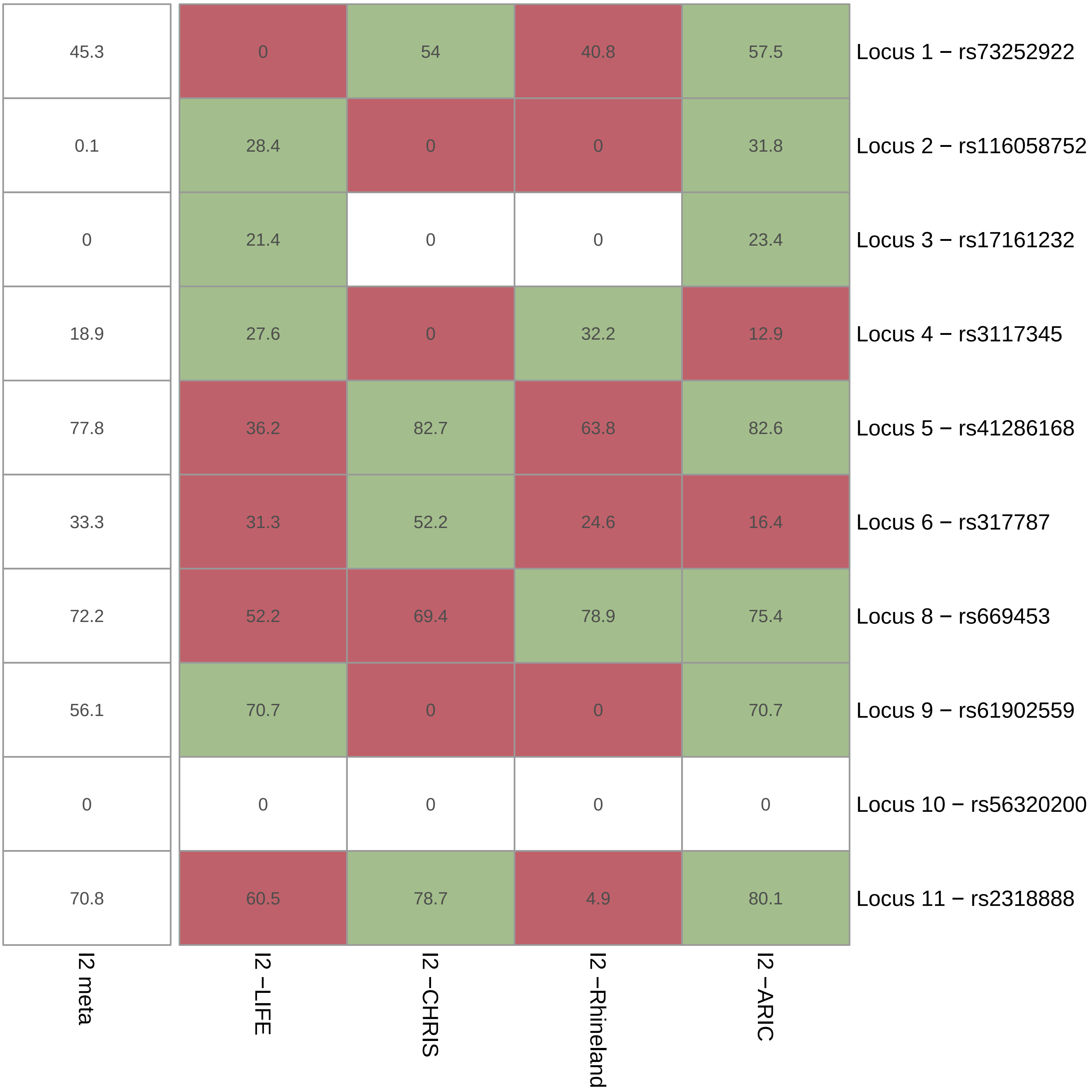

Supplementary Figure 20: Sensitivity analysis for drivers of heterogeneity for index variants of independent genome-wide significant loci. I^2 was recalculated based on the statistics of the best associated phenotype by leaving out the study specified on the x-axis. The y-axis lists the locus and variant. Red colour indicates a reduced heterogeneity when leaving out the study, whereas green indicates an increased heterogeneity compared to the original meta-analysis. White indicates unchanged heterogeneity. I^2 values are given in percent.
