# Supplementary material for: Genome-wide association meta-analysis of human olfactory identification discovers sex-specific and sex-differential genetic variants
Source: Nat Commun. 2025 Jul 1;16:5434. doi: 10.1038/s41467-025-61330-y (PMC12219263; doi:10.1038/s41467-025-61330-y)
Supplement: Supplementary file 2 — Description of Additional Supplementary Files [file 41467_2025_61330_MOESM2_ESM.pdf]

Description of Additional Supplementary Files

Supplementary Data 1: Study characteristics and used software for the four studies used in this meta-analysis.

Supplementary Data 2: Number of correct and incorrect odour identifications and median identification score per participating study.

Supplementary Data 3: Number of SNPs and λ values per trait-subgroup combination before and after QC.

Supplementary Data 4: Heritability estimates and LD Score regression intercepts for olfactory traits.

Supplementary Data 5: Results from MR-MEGA regression for index-variants of genome-wide significant loci.

Supplementary Data 6: Annotation results of 99% credible sets.

Supplementary Data 7: Genes considered for candidate selection. For each locus, all genes within a +/-250kb window around variants included in the respective credible set were used in the search for candidate genes.

Supplementary Data 8: Odds ratios for index variants harmonized for effect direction.

Supplementary Data 9: Comparisons between sexes.

Supplementary Data 10: Annotation of EREs and AREs for loci with sex-differential effects.

Supplementary Data 11: Results from colocalization analysis between odour identification and xQTLs from blood and brain.

Supplementary Data 12: Genetic correlation between coffee identification in overall analysis and intake of coffee or tea.

Supplementary Data 13: Conditional analysis of sex-wise index variants for loci with support for independent male and female signals.

Supplementary Data 14: Identified components of the 16 item 'Sniffin' Sticks' screening test.

Supplementary Data 15: Loci with suggestive association with olfactory misidentification.

Supplementary Data 16: Effect size comparisons between sexes for suggestive loci.

Supplementary Data 17: Annotation of index variants of suggestive loci.

Supplementary Data 18: Instrumental variants used in the first MR of sex hormones on odour perception.

Supplementary Data 19: Inverse-variance weighted (IVW) two-sample Mendelian randomization (MR) estimates of hormones on odour perception.

Supplementary Data 20: Instrumental variants used in the MR of neurodegenerative diseases on the odour identification score.

Supplementary Data 21: Inverse-variance weighted (IVW) two-sample Mendelian randomization (MR) estimates of neurodegenerative diseases on odour identification score.

Supplementary Data 22: Estimates of other two-sample MR analyses for neurodegenerative diseases on the odour identification score.

Supplementary Data 23: Instrumental variants used in the MR of odour perception on neurodegenerative diseases.

Supplementary Data 24: Results for the MR of odour perception on neurodegenerative diseases.

Supplementary Data 25: Genetic correlation between the odour identification phenotypes and common complex diseases.

Supplementary Data 26: LD-score regression intercepts based on summary statistics from participating studies.

Supplementary Data 27: Colocalization results for MR of sexual hormones on olfactory identification.
