# Supplementary material for: Genome-wide association meta-analysis of human olfactory identification discovers sex-specific and sex-differential genetic variants
Source: Nat Commun. 2025 Jul 1;16:5434. doi: 10.1038/s41467-025-61330-y (PMC12219263; doi:10.1038/s41467-025-61330-y)
Supplement: Supplementary file 4 — Reporting Summary [file 41467_2025_61330_MOESM4_ESM.pdf]

nature portfolio

Corresponding author(s): Franz Förster, Markus Scholz, Christian Fuchsberger

Last updated by author(s): Jun 11, 2025

# Reporting Summary

Nature Portfolio wishes to improve the reproducibility of the work that we publish. This form provides structure for consistency and transparency in reporting. For further information on Nature Portfolio policies, see our Editorial Policies and the Editorial Policy Checklist.

## Statistics

For all statistical analyses, confirm that the following items are present in the figure legend, table legend, main text, or Methods section.

| n/a | Confirmed | |
|---|---|---|
| ☐ | ☒ | The exact sample size ($n$) for each experimental group/condition, given as a discrete number and unit of measurement |
| ☐ | ☒ | A statement on whether measurements were taken from distinct samples or whether the same sample was measured repeatedly |
| ☐ | ☒ | The statistical test(s) used AND whether they are one- or two-sided<br>*Only common tests should be described solely by name; describe more complex techniques in the Methods section.* |
| ☐ | ☒ | A description of all covariates tested |
| ☐ | ☒ | A description of any assumptions or corrections, such as tests of normality and adjustment for multiple comparisons |
| ☐ | ☒ | A full description of the statistical parameters including central tendency (e.g. means) or other basic estimates (e.g. regression coefficient) AND variation (e.g. standard deviation) or associated estimates of uncertainty (e.g. confidence intervals) |
| ☐ | ☒ | For null hypothesis testing, the test statistic (e.g. $F$, $t$, $r$) with confidence intervals, effect sizes, degrees of freedom and $P$ value noted<br>*Give P values as exact values whenever suitable.* |
| ☐ | ☒ | For Bayesian analysis, information on the choice of priors and Markov chain Monte Carlo settings |
| ☒ | ☐ | For hierarchical and complex designs, identification of the appropriate level for tests and full reporting of outcomes |
| ☐ | ☒ | Estimates of effect sizes (e.g. Cohen's $d$, Pearson's $r$), indicating how they were calculated |

*Our web collection on statistics for biologists contains articles on many of the points above.*

## Software and code

Policy information about availability of computer code

| | |
|---|---|
| Data collection | R v4.4.1 |
| Data analysis | R v4.4.1, Plink 2.0, SAIGE, regenie, METAL, GCTA v1.92.0b3, LDSC v1.0.1, VCF-liftover, SMR v1.3.1, MR-MEGA v.0.2<br><br>All analysis scripts are available on Zenodo (https://doi.org/10.5281/zenodo.15606619).<br><br>Rpackages:<br>AnnotationDbi v1.64.1, Biobase v2.62.0, BiocFileCache v2.10.1, BiocGenerics v0.48.1, BiocIO v1.12.0, BiocParallel v1.36.0, Biostrings v2.70.2, CMplot v4.5.1, DBI v1.2.3, DEoptimR v1.1-3, DelayedArray v0.28.0, Formula v1.2-5, GenomeInfoDb v1.38.6, GenomeInfoDbData v1.2.11, GenomicAlignments v1.38.2, GenomicRanges v1.54.1, Hmisc v5.1-1, IRanges v2.36.0, KEGGREST v1.42.0, KernSmooth v2.23-22, MASS v7.3-60.0.1, Matrix v1.6-5, MatrixGenerics v1.14.0, MatrixModels v0.5-3, MendelianRandomization v0.10.0, R.methodsS3 v1.8.2, R.oo v1.26.0, R.utils v2.12.3, R6 v2.5.1, RColorBrewer v1.1-3, RCurl v1.98-1.14, RSQLite v2.3.5, Rcpp v1.0.12, Rsamtools v2.14.0, S4Arrays v1.2.1, S4Vectors v0.40.2, SparseArray v1.2.4, SparseM v1.81, SummarizedExperiment v1.32.0, UpSetR v1.4.0, WriteXLS v6.5.0, XML v3.99-0.16.1, XVector v0.42.0, abind v1.4-5, arrangements v1.1.9, backports v1.5.0, base64enc v0.1-3, biomaRt v2.58.2, bit v4.0.5, bit64 v4.0.5, bitops v1.0-7, blob v1.2.4, brio v1.1.5, broom v1.0.5, caTools v1.18.2, cachem v1.0.8, car v3.1-2, carData v3.0-5, cellranger v1.1.0, checkmate v2.3.1, cli v3.6.2, cluster v2.1.6, codetools v0.2-19, coloc v5.2.3, colorspace v2.1-0, compiler v4.4.2, corrplot v0.92, crayon v1.5.2, curl v5.2.0, data.table v1.14.8, data.table (qtl coloc) v1.16.2, dbplyr v2.4.0, digest v0.6.35, doParallel v1.0.17, dplyr v1.1.4, eulerr v7.0.0, evaluate v0.24.0, fansi v1.0.6, fastmap v1.2.0, fdrtool v1.2.17, filelock v1.0.3, foreach v1.5.2, foreign v0.8-86, forestplot v3.1.3, formatR v1.14, generics v0.1.3, genpwr v1.0.4, ggmagnify v0.2.0, ggplot2 v3.4.4, ggpubr v0.6.0, ggrepel v0.9.5, ggsignif v0.6.4, glmnet v4.1-8, glue v1.7.0, gmp v0.7-4, gplots v3.1.3.1, gridExtra v2.3, gtable v0.3.4, gtools v3.9.5, gtx v0.0.8, hms v1.1.3, htmlTable v2.4.2, htmltools v0.5.7, htmlwidgets v1.6.4, httr v1.4.7, irlba v2.3.5.1, iterators v1.0.14, iterpc v0.4.2, jsonlite v1.8.8, knitr v1.45, lattice v0.22-5, lazyeval v0.2.2, lemon v0.4.9, lifecycle v1.0.4, magrittr |

v2.0.3, mathjaxr v1.6-0, matrixStats v1.3.0, memoise v2.0.1, metadat v1.2-0, metafor v4.6-0, mixsqp v0.3-54, munsell v0.5.1, nleqslv v3.3.5, nlme v3.1-164, nnet v7.3-19, numDeriv v2016.8-1.1, openxlsx v4.2.5.2, pheatmap v1.0.12, pillar v1.9.0, pkgconfig v2.0.3, plotly v4.10.4, plyr v1.8.9, png v0.1-8, prettyunits v1.2.0, progress v1.2.3, purrr v1.0.2, quantreg v5.97, rJava v0.9-13, rappdirs v0.3.3, readxl v1.4.3, reshape v0.8.9, reshape2 v1.4.4, restfulr v0.0.15, rjson v0.2.21, rlang v1.1.3, rmarkdown v2.25, robustbase v0.99-2, rpart v4.1.23, rstatix v0.7.2, rstudioapi v0.15.0, rtracklayer v1.62.0, scales v1.3.0, shape v1.4.6, splines v4.4.2, stringi v1.7.12, stringr v1.5.1, survival v3.5-8, susieR v0.12.35, testthat v3.2.1, tibble v3.2.1, tidyr v1.3.1, tidyselect v1.2.0, toolboxH v0.2.15, tools v4.4.2, utf8 v1.2.4, vctrs v0.6.5, viridis v0.6.5, viridisLite v0.4.2, withr v3.0.0, xfun v0.42, xlsx v0.6.5, xlsxjars v0.6.1, xml2 v1.3.6, yaml v2.3.8, zip v2.3.1, zlibbioc v1.48.0

For manuscripts utilizing custom algorithms or software that are central to the research but not yet described in published literature, software must be made available to editors and reviewers. We strongly encourage code deposition in a community repository (e.g. GitHub). See the Nature Portfolio guidelines for submitting code & software for further information.

## Data

Policy information about availability of data

All manuscripts must include a data availability statement. This statement should provide the following information, where applicable:

- Accession codes, unique identifiers, or web links for publicly available datasets
- A description of any restrictions on data availability
- For clinical datasets or third party data, please ensure that the statement adheres to our policy

Summary statistics for this study have been deposited in the Leipzig Health Atlas under accession code 8VK7H50F6P-6 (https://www.health-atlas.de/assays/88). Further data are provided in the Supplementary Data file. The raw individual level data are protected and are not publicly available due to data privacy laws but can be requested from individual studies. Data of the Rhineland study can be requested by researchers in accordance with the Rhineland Study's Data Use and Access Policy (RS-DUAC@dzne.de). Access to CHRIS data can be provided for research purposes upon request to the CHRIS Access Committee (access.request.biomedicine@eurac.edu). Data of the LIFE-Adult-Study are available based on written project agreements and data transfer agreements according to the centre's data use and access policies. ARIC data from visit 1 to visit 5 are available through the Biologic Specimen and Data Repository Information Coordinating Center (BioLINCC). Data that are not yet available through BioLINCC are available upon request through the ARIC Coordinating Center at the University of North Carolina. Data sets used in the study are Ensembl 2018 (http://www.ensembl.org/index.html), CADD (https://cadd.gs.washington.edu/download), GTEx V8 (https://gtexportal.org/home/protectedDataAccess), pan-UKBB (https://pan.ukbb.broadinstitute.org/downloads/) FinnGen (https://www.finngen.fi/en/access_results) and publicly available summary statistics for sex hormones (https://www.ebi.ac.uk/gwas/publications/32042192), xQTLs (https://yanglab.westlake.edu.cn/software/smr/#DataResource) as well as intake of coffee and tea (https://yanglab.westlake.edu.cn/pub_data.html).

## Research involving human participants, their data, or biological material

Policy information about studies with human participants or human data. See also policy information about sex, gender (identity/presentation), and sexual orientation and race, ethnicity and racism.

| | |
|---|---|
| Reporting on sex and gender | Assigned biological sex was considered in the study design. We provided sex-specific and sex-combined analysis results. We also performed genetic sex-interaction analyses. |
| Reporting on race, ethnicity, or other socially relevant groupings | Genetic ancestry of study participants was collected according to self-reported ancestries. Only individuals with European ancestry were included in this study, since sample sizes of other ethnicities were too small to be analysed. |
| Population characteristics | We performed a meta-analysis of four studies. Study-wise mean age ranged from 45 to 76 years, the fraction of smokers varied between 6% and 20%. Sex ratios were balanced across all studies (51%-56% females). |
| Recruitment | Different recruitment modalities of participating studies are provided as short study descriptions or specific publications of the respective cohorts. |
| Ethics oversight | All participating studies were approved by respective ethics committees, namely the ethics committee of the University of Bonn, Medical Faculty (reference ID: 338/15), the Ethics Committee of the Health Authority of the Autonomous Province of Bolzano (Südtiroler Sanitätsbetrieb/Azienda Sanitaria dell'Alto Adige; protocol No. 21/2011, 19 April 2011), the ethics committee of the University of Leipzig, Germany (Reg. No. for LIFE-Adult: 263-2009-14122009 and 201/17-ek), and each institution of the ARIC study has Institutional Review Board approval. |

Note that full information on the approval of the study protocol must also be provided in the manuscript.

# Field-specific reporting

Please select the one below that is the best fit for your research. If you are not sure, read the appropriate sections before making your selection.

[x] Life sciences [ ] Behavioural & social sciences [ ] Ecological, evolutionary & environmental sciences

For a reference copy of the document with all sections, see nature.com/documents/nr-reporting-summary-flat.pdf

# Life sciences study design

All studies must disclose on these points even when the disclosure is negative.

| | |
|---|---|
| Sample size | Due to the hypothesis-free nature of genome-wide association studies, we used the maximum number of available sample sizes for the traits considered in this study. Sample size was determined on a per-variant basis by counting all individuals where the genetic variant successfully |

| | |
|---|---|
| | passed quality metrics. |
| Data exclusions | Exclusion of subjects was performed on a per-study basis, primarily based on missing pheno- or genotype information or low genotyping quality. Study-wise exclusion procedures are provided as supplement. |
| Replication | No experimental replication was performed. We conducted a full meta-analysis summarising all available evidence. |
| Randomization | Not applicable. We mostly used data from non-randomized observational studies. Covariate effects were considered during regression analysis. |
| Blinding | Blinding was not meaningful in our analysis context, as no group comparisons were performed. |

# Reporting for specific materials, systems and methods

We require information from authors about some types of materials, experimental systems and methods used in many studies. Here, indicate whether each material, system or method listed is relevant to your study. If you are not sure if a list item applies to your research, read the appropriate section before selecting a response.

## Materials & experimental systems

| n/a | Involved in the study |
|---|---|
| ☒ | ☐ Antibodies |
| ☒ | ☐ Eukaryotic cell lines |
| ☒ | ☐ Palaeontology and archaeology |
| ☒ | ☐ Animals and other organisms |
| ☒ | ☐ Clinical data |
| ☒ | ☐ Dual use research of concern |
| ☒ | ☐ Plants |

## Methods

| n/a | Involved in the study |
|---|---|
| ☒ | ☐ ChIP-seq |
| ☒ | ☐ Flow cytometry |
| ☒ | ☐ MRI-based neuroimaging |

## Plants

| | |
|---|---|
| Seed stocks | NA |
| Novel plant genotypes | NA |
| Authentication | NA |
